# Acute anti-obesity treatment with celastrol reduces body weight, cerebral inflammation and metabolic imbalances in mice

**DOI:** 10.1186/s10020-026-01530-4

**Published:** 2026-07-01

**Authors:** Adriana Ferreiro, Maya Holgado, Raquel González-Alday, Sara González-Soto, Lidia M. Fernandez-Sevilla, Ángeles Vicente, Pilar López-Larrubia, Blanca Lizarbe

**Affiliations:** 1 Institute for Biomedical Research Sols-Morreale (IIBM), Spanish National Research Council-Autonomous University of Madrid, c/Arturo Duperier 4, Madrid, 28029 Spain; 2 Department of Cell Biology and Histology, Faculty of Medicine, UCM, Madrid, 28040 Spain; 3 Department of Basic Health Sciences, Faculty of Health Sciences, University Rey Juan Carlos, Alcorcón, 28922 Spain; 4 Stem Cells, Immunity and Cancer Group, Health Research Institute Hospital, 12 de Octubre (I+12), Madrid, Spain; 5 Biomedical Research Networking Centre On Rare Diseases (CIBERER), Institute of Health Carlos III, Madrid, Spain; 6 Department of Biochemistry, Autonomous University of Madrid, Madrid, Spain

**Keywords:** Brain, Celastrol, Magnetic Resonance Imaging, Magnetic Resonance Spectroscopy, Inflammation, Obesity, Mouse, Metabolism

## Abstract

**Background:**

The global rise in obesity is predominantly driven by energy dense foods consumption and sedentary lifestyles that contribute to a growing burden of metabolic and neuroinflammatory comorbidities. Obesity is linked to a chronic low-grade inflammatory profile, as well as to a localized neuroendocrine imbalance and inflammatory response in the brain, including regions regulating energy homeostasis, reward and motivational centers. Anti-obesity medications that reduce body weight are being extensively used across the world, and the specific cerebral mechanisms underlying its action are yet to be clarified.

**Methods:**

We investigated the cerebral and systemic effects inherent to obesity development and treatment with celastrol, an anti-obesity and anti-inflammatory agent, in a murine model of diet-induced obesity (DIO) using a multimodal approach. We characterized obesity progression and celastrol acute treatment by comparing body weight (BW), food intake, changes in brain microstructure by in vivo magnetic resonance imaging (MRI) and ex vivo by immunofluorescence (IF), investigated its metabolic rearrangements using ^1^H high-resolution magic angle spinning spectroscopy and draw the hormonal profiles between DIO and control animals, with or without treatment.

**Results:**

Our findings indicate that obesity induces detectable neuroinflammation, evident through diffusion MRI alterations and increased glial activation, with quantifiable morphological changes. Treatment resulted in significant BW reduction, diffusion MRI signal changes, particularly in the hypothalamus, a decrease in glial activation, a regularization of cerebral osmolyte concentrations, decreased cellular proliferation and astrocytic metabolism markers, and anti-inflammatory cytokine changes.

**Conclusions:**

These results support the role of celastrol as an anti-obesity treatment, with anti-inflammatory effects in the hypothalamus and associated cerebral metabolic rearrangements, and prove MRI techniques as valid tools to characterize its effects.

**Supplementary Information:**

The online version contains supplementary material available at 10.1186/s10020-026-01530-4.

## Background

Consumption of energy dense foods, such as high-fat and sugar diets (HFHS), combined with sedentary lifestyles, are among the most important environmental factors predisposing to obesity. During obesity, the accumulation of elevated fat stores triggers a low-grade systemic inflammation, characterized by an abnormal cytokine production and the activation of a network of inflammatory signaling pathways (Wellen and Hotamisligil 2005), which boost the development of the obesity-related diseases (Hotamisligil 2006). Particularly, inflammation affects multiple organs including, pancreas, liver, cardiovascular system and the brain, eventually leading to the disruption of global metabolic homeostasis (Uranga and Keller 2019).

HFHS feeding in rodents is an extensively used model to investigate its onset and development (De Moura E Dias et al. 2021; Nilsson et al. 2012). In the last decade, several studies using animal models of diet-induced obesity (DIO) revealed the activation of a localized inflammatory response in the hypothalamus (Hyp) after short term HF feeding that induces a defective control of energy homeostasis and development of leptin and insulin resistance (Thaler et al. 2012; Valdearcos et al. 2017a; Zhang et al. 2008). During fat-rich diets consumption, long-chain saturated fatty acids (SFAs) cross the blood–brain barrier and bind to the pro-opiomelanocortin neurons in the arcuate nucleus (ARC) of the Hyp (Posey et al. 2009). This binding triggers the activation of inflammatory signaling cascades, prompting the expression of pro-inflammatory genes. In this circumstances, glial cells experience morphological, physiological and functional modifications that enable an inflammatory process against the accumulation of SFAs (García-Cáceres et al. 2019; Ramalho et al. 2018; Valdearcos et al. 2014, 2017). Particularly, astrocytes develop a reactive phenotype in the ARC detected immunohistochemically 24 h after HF intake (Buckman et al. 2015; Horvath et al. 2010; Thaler et al. 2012), release inflammatory cytokines(Gupta et al. 2012), and, in response to high leptin levels, trigger microvascular remodeling within the Hyp (Gruber et al. 2021; Yi et al. 2012). Interestingly, few studies have reported that is the carbohydrate component of fat-rich diets which initiates such inflammatory cascade, including microglial activation and angiogenesis (Gao et al. 2017).

In mammals, the homeostatic system interacts with motivational and rewarding behaviors via the mesocorticolimbic complex (MC) and reward centers (RC), respectively (Ferrario et al. 2016), cerebral structures that participate in the control of food intake and can exert relevant roles in favoring obesity development. MC structures include abundant dopamine projections from the ventral tegmental area to the prefrontal cortex, amygdala, hippocampus (Hipp), nucleus accumbens (NAc) and infralimbic area (ILA), correlating potential appetite stimuli to associated rewards, thus creating motivational connections (Berridge 2009). RCs include regions from the orbitofrontal cortex, amygdala and NAc, and grant food with its pleasurable properties (Saper et al. 2002). Interestingly, some of the implicated MC and RC regions also express inflammatory signals during obesity development (Cazettes et al. 2011).

Celastrol, a pentacyclic triterpene extracted from the roots of the *Tripterygium Wilfordi* plant, was first revealed as a promising anti-obesity agent in 2013, by Kim and colleagues (Kim et al. 2013); results that were confirmed later on by Weisberg and coworkers (Weisberg et al. 2016). In those works, authors showed that at doses of 1 or 3 mg/kg, celastrol decreased BW and blood glucose values and improved insulin sensitivity on leptin receptor-deficient (db/db) mice. Further studies at lower doses (0.1 mg/kg) demonstrated that celastrol administration could reduce BW up to 45% in long term wild type DIO animals, decreasing food intake and improving glucose homeostasis, but that such dose was ineffective in leptin-deficient (ob/ob) and db/db mouse (Liu et al. 2015). Interestingly, authors proved that such effects were linked to celastrol-induced enhanced leptin sensitivity and reduced hypothalamic endoplasmic reticulum stress. Other studies have recapitulated those findings, reporting that at higher doses peripheral mechanisms of action exist (Ma et al. 2015), and that celastrol-induced weight loss is hypophagia driven and age-dependently mediated by functional leptin signaling (Pfuhlmann et al. 2018). More recent works have proposed additional mechanisms, including inhibition of inflammatory signaling, modulation of lipid metabolism, and effects on gut microbiota (Feng et al. 2019; Seyfried and Hankir 2019; Xu et al. 2021). Overall, although celastrol consistently shows metabolic benefits in obese mice, the exact mechanisms underlying its effects remain incompletely understood, and may vary according to dose, model, and treatment duration (Saito et al. 2019).

A variety of neuroimaging methods have shown that obesity is associated with brain inflammation, alterations in the cerebral microstructure, metabolism, and function. Among them, magnetic resonance imaging (MRI) techniques, such as diffusion MRI (dMRI) have provided evidence of cerebral inflammation during high-fat diet (HFD) feeding in mice, and on patients with obesity (Le Bihan 2013). dMRI, and particularly diffusion tensor imaging (DTI) methods are widely used in clinics and in basic research and have revealed important cerebral alterations during obesity (Lizarbe et al. 2020a). For example, it has been described that obese patients and mice depict higher diffusivity in particular areas of the brain, as compared to non-obese individuals, which has been proposed to be a consequence of vasogenic edema related to obesity-induced blood brain barrier permeability changes (Cheung et al. 2009; Thomas et al. 2019) but the underlying mechanisms have not been elucidated. Notably, other MRI techniques, such as T_2_-weighted imaging or magnetization transfer imaging (MTI) have also been used to reveal changes in brain microstructure in the context of obesity-induced brain changes (Rosenbaum et al. 2022). On the other hand, magnetic resonance spectroscopy (MRS) techniques, such as high-resolution magic angle spinning (HRMAS), are sensitive enough to detect diet-induced metabolic changes in small regions of the mouse brain, such as the hypothalamus (Campillo et al. 2022; Frost et al. 2014). Interestingly, the cerebral metabolic changes induced by anti-obesity medications administration are not yet completely understood. In this sense, the implementation of imaging and spectroscopic techniques is endowed to provide vital information on the cerebral changes underlying obesity development and treatment.

On these grounds, we designed an experimental setup in which we administered either HFHS or low-fat low sugar (LFLS) diets to male and female animals and followed the development of obesity and its effects on the brain by MRI as well as the plasma concentrations of the main peptides and hormones involved in appetite and energy balance, assessed via ELISA. Subsequently, we treated the animals with celastrol and the effects on the brain were assessed using MRI, regional neurochemical profiles using ^1^H HRMAS, glial markers by quantitative IF and ELISA analyses.

Using this methodology, we tested the hypothesis that celastrol induces detectable brain changes in DIO mice observable in vivo using DTI parameters and magnetization transfer ratio (MTR) and focusing on four regions involved in both homeostatic and non-homeostatic control of food intake: the Hyp, the Hipp, the NAc, and the ILA. Additionally, we postulated that these changes would be corroborated ex vivo using IF, ^1^H HRMAS and ELISA techniques.

## Methods

### Experimental design

All animal procedures were performed at the Institute for Biomedical Research Sols-Morreale (Reg. No. ES280790000288) and were approved by the institutional Animal Welfare Ethics Committee and by the competent regional authority (Comunidad de Madrid), under animal protocol approval number PROEX 150.5/22, in accordance with European and Spanish regulations on the protection of animals used for scientific purposes (Directive 2010/63/EU; Spanish RD 53/2013). C57BL/6 mice, bred and housed in our institutional animal facility, were accommodated in groups of 2–5 animals, with 12-h/12-h light/dark cycle, controlled humidity (45–55%), temperature (21–23ºC) and ad libitum access to food and water. At nine weeks old, mice were randomly divided into two different dietary groups, LFLS (Research Diets, D12450Hi, 10 kcal% Fat) or a HFHS diet (Research Diets, D08112601i, 45 kcal% Fat with 30 kcal% Sucrose). BW and food intake were recorded weekly. Following a 20-week period of diet, we administered i.p. either celastrol (C0869, Sigma-Aldrich, Merck) diluted it in dimethyl sulfoxide (DMSO) (1%) and PBS to a final concentration of 0.04 mg/mL, or only PBS solution with 1% DMSO. Each animal received a dose of 0.25 mg/kg or vehicle solution for three consecutive days.

**Fig. 1:**
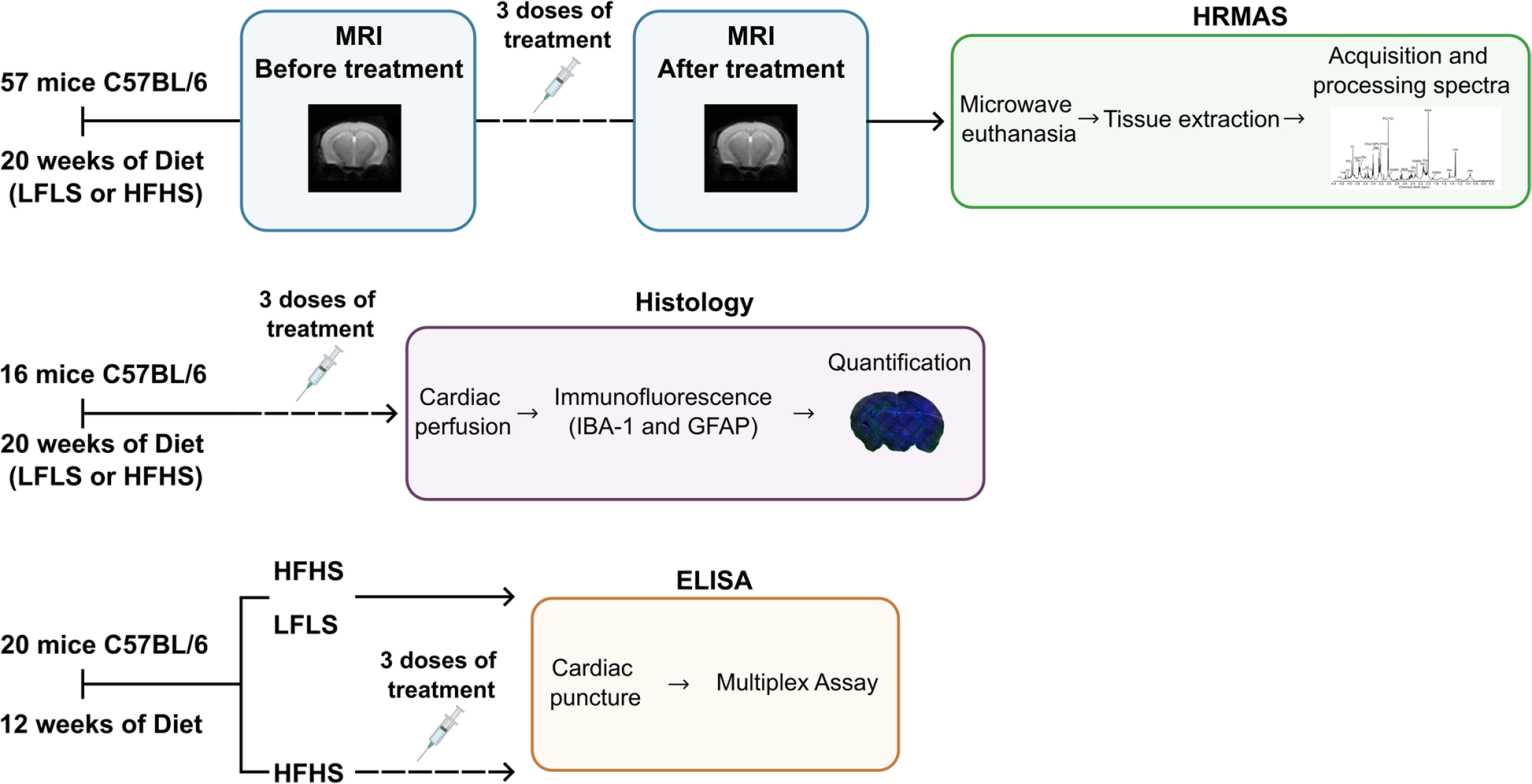
Experimental design. C57BL/6 mice were divided into three cohorts. The MRI/HRMAS cohorts (*n* = 57) received LFLS or HFHS diet for 20 weeks, underwent MRI before and after three doses of treatment, and were then processed for HRMAS. The histology cohorts (*n* = 16) received LFLS or HFHS diet for 20 weeks, followed by treatment. The ELISA cohort (*n* = 20) received LFLS or HFHS diet for 12 weeks, with HFHS mice assigned to treated or untreated groups

Experimental methods to assess the cerebral effects of diet consumption and subsequent treatment with celastrol included: MRI scans after 20 weeks of diet (“*diet* effects”) to male and female animals and post-treatment (“*treatment* effects”) to the same animal batches (i); neurochemical profiles by HRMAS of the brain regions of interest (ii); histological markers of astrogliosis and microgliosis (iii); and plasma levels of main hormones involved in appetite regulation and energy balance (iv) (Table 1). Additionally, plasma was analyzed also before any treatment (*n* = 10 LFLS and *n* = 10 HFHS, 50% females) (Fig. 1). Sample sizes were estimated based on previous studies of MRI quantification of cerebral changes during obesity (Campillo et al. 2022), with similar expected effect sizes and statistical power, and assuming that the rest of techniques exhibit at least comparable effects.

**Table 1:** Distribution of number of animals for each group depending on the diet, technique and treatment

Treatment	Celastrol				Vehicle			
Diet/Technique	MRI	Hist	HR-MAS	Elisa	MRI	Hist	HR-MAS	Elisa
HFHS	7♀ 7♂	2♀ 2♂	5♀6♂	5 ♀5♂	7 ♀7♂	2♀ 2♂	3♀3♂	5 ♀5♂
LFLS	9♀6♂	2♀ 2♂	6 ♀6♂		7♀ 7♂	2♀ 2♂	9♀7♂

### MRI acquisition

MRI scans of the mouse brain were performed on a Bruker Biospec 7 T system (Bruker Biospin, Ettlingen, DE) and the management software was Paravision 6.0.1, equipped with a ^1^H mouse head surface coil with a volume transmitter (90 mm diameter gradient insert 360mT/m). During the MRI experiments, each mouse was individually anesthetized in a methacrylate induction box with isoflurane (2% 1 L/min O_2_) and sustained throughout the acquisition using a nose mask with isoflurane (1% 1 L/min O_2_). The anesthetized animals were placed on a bed equipped with a circulating warm water bath to maintain their body temperature at approximately 37 °C. The head of each mouse was fixed using a tooth-bar and ear-bars. Throughout the experiment, the body temperature and respiration of the animals were continuously monitored. Localization of the regions of interest (ROI), was achieved by acquiring axial T_2_-weighted anatomical images, using a rapid acquisition with relaxation enhancement sequence with the following parameters: FOV = 21 × 21 mm^2^, repetition time (TR) = 2500 ms, echo time (TE) = 27 ms, RARE factor = 8, number of averages (Av) = 1, 5 slices in an axial orientation, slice thickness = 1.25 mm.

DTI data were acquired using a Stejskal–Tanner sequence (TR = 3000 ms, TE = 32.56 ms, gradient separation (Δ) = 20 ms, gradient duration (δ) = 4 ms, FOV = 21 × 21 mm^2^, slice thickness of 1.25 mm, diffusion gradients in 15 uniformly distributed directions, b = 400 smm^−2^ and 1800 smm^−2^, and 3 b = 0 smm^−2^). Two sets of MTI (TR = 2500 ms, TE = 9.8 ms, and Av = 1) were acquired, either with an MT pulse applied (MT ON, *N* = 50 train of radio frequency pulses, power = 5.5 µT, offset = 1500 Hz) or without (MT OFF), and the corresponding MTR calculated as the normalized subtraction of the corresponding signal intensities.

### MRI processing

MRI data were processed using a software based on Python, to obtain the mean diffusivity (MD), fractional anisotropy (FA), axial diffusivity (AD), radial diffusivity (RD), and MTR maps, for the Pre- and Post*-treatment* datasets, respectively, with Dipy (Garyfallidis et al. 2014). The pre-processing pipeline encompassed the use of the Patch2self (Fadnavis et al. 2020) noise reduction filter for DTI and the adaptive soft coefficient matching (ASCM) filter (Coupé P. et al. 2012) for MTR. The Hyp, Hipp, NAc and ILA areas were manually delineated using ImageJ (U. S. National Institutes of Health, Bethesda, Maryland, USA, https://imagej.nih.gov/ij/) with a standard mouse atlas as a reference http://www.brain-map.org. Pixel values of each parametric map were automatically filtered to remove extreme outliers (1st/3rd quartile ± 1.5*interquartile range) and regions close to cerebrospinal fluid (CSF) from the ventricles (MD > 1300 μm^2^/s) and MTR < 0. From the remaining pixels, mean values were calculated for each region.

### HRMAS

Immediately after the MRI sessions, a group of animals was euthanized under the effects of anesthesia, using a high-power microwave (TMW-4012C 5 kW, Muromachi Kikai Co. Ltd., Japan). This method applies focused microwave irradiation to the brain and preserves in vivo metabolic state of the cerebral tissue by rapid denaturation of enzymes responsible for protein dephosphorylation (O’Callaghan and Sriram 2004). After euthanasia, brains were extracted from the skull, and tissue samples were collected from the same regions selected in MRI analysis: Hyp, Hipp, ILA and NAc and stored immediately in liquid nitrogen, to be preserved at −80 °C to prevent deterioration.

HRMAS experiments were performed on an 11.7 T Bruker Avance Neo vertical system (Bruker Biospin, Ettlingen, DE) operating at a proton frequency of 500.13 MHz and Topspin 4.1.4 software. The preparation of the sample consisted of introducing 10–15 mg of tissue into a zirconium rotor (diameter of 4 mm), 50 µl of D_2_O was added and then hermetically sealed. The measurements were performed at a constant temperature of 277 K with a spinning rate of 5000 Hz. For each sample, two spectra were acquired using a Carr-Purcell-Meiboom-Gill sequence (128 scans, a relaxation delay of 2 s, a water suppression pulse of 2 s, 32 K data points and two different TE of 36 ms and 144 ms, to account for both the metabolites with strong J-coupling, and also obtain cleaner baselines (Oz and Tkáč 2011)).

The data was processed using LCModel, a software designed for the quantification of metabolites (Provencher 2001). To achieve this, LCModel fits each spectrum as a linear combination of their correspondent values from a homemade database. Such database was created by acquiring the spectra of individual metabolite from the brain. For each metabolite, the software calculates its concentration, the estimated percentage standard deviation (%SD), and concentrations normalized to total creatine (PCr + Cr) content. The data base is composed of the following metabolites and macromolecules: alanine (Ala), aspartate (Asp), choline (Cho), creatine (Cr), GABA, glucose (Glc), glutamine (Gln), glutamate (Glu), glycine (Gly), glycerylphosphorylcholine (GPC), glutathione (GSH), lactate (Lac), leucine (Leu), myo-inositol (mI), N-acetyl-aspartate (NAA), N-acetylaspartylglutamate (NAAG), phosphocholine (PCh), phosphocreatine (PCr), phenylalanine (Phe), taurine (Tau), the lipids including Lip13a, Lip09, Lip20, macromolecules such as MM09, MM20, MM12 and the sums Cho + GPC + PCh, NAA + NAAG, Cr + PCr, Glu + Gln, MM14 + Lip13a + Lip13b + MM12, MM09 + Lip09 and MM20 + Lip20, among others. Only metabolites with a %SD less than 30% were included in the statistics analysis.

### Histology

A group of mice were transcardially perfused with phosphate-buffered saline (PBS) and paraformaldehyde (PFA). Their brains were removed and placed in 4% PFA for 24 h, followed by the immersion in a 30% sucrose solution for 48 h, and then fixed in OCT (Tissue-Tek, Miles, Elkhart, In., EEUU) to be subsequently stored at −80ºC to ensure cryopreservation. Frozen coronal sections were obtained using a cryostat (Shandon Cryotome E; Thermofisher Scientific Inc, Waltham, Massachusetts, USA) at a thickness of 7 μm and mounted on glass slides.

Coronal sections were first treated with a solution of PBS (3% H_2_O_2_ and 10% methanol) and then blocked in PBS with 10% normal donkey serum (0.1% Gly, 0.02% Triton-X). The sections were then incubated overnight at 4ºC with primary antibodies for Iba-1 (DAKO,1:500) and GFAP (Merck Millipore, 1:600) to label microglia and astrocytes respectively. Following PBS washes, the sections were incubated with the specific secondary antibodies for Iba-1 (Alexa Fluor 594) and GFAP 1 (Alexa Fluor 488) (Thermo Fisher Scientific, 1:300). Hoechst 33,258 (Molecular Probes, 24 Invitrogen) was used for nuclear staining. The procedure followed is detailed in (Fernández-Sevilla et al. 2022) and (Fernández‐Sevilla et al. 2020). Slides were mounted with Prolong Gold (Life Technologies) and images were acquired using a Nikom Eclipse Ci fluorescence microscopy with a Nikon Digital Sight DS-U3 camera and Nis-Elements D Viewer software.

For the acquisition of images, a 20 × objective was used for the Hyp and Hipp, and a 10 × objective for the ILA and NAc. For each animal, a total of 21 images were captured from the Hyp (3 images from the ARC, 2 from the ventromedial nucleus (VMN), 2 from the paraventricular nucleus (PVH) per slice), 12 images from the Hipp (4 per slice), 12 images from the NAc (4 per slice) and 9 images from the ILA (per slice), with each animal having 3 slices.

Image analysis was conducted using ImageJ. The quantification process involved each image containing the entire quadrant, or a specific area of interest, excluding bubbles or non-brain tissue regions. In each image, we measured the area fraction occupied by cells (%OA), the number of cells (C/A), average size, perimeter, circularity and solidity. For the area fraction occupied by cells, the values from all images were added and then divided by the number of images for each animal and brain region. The count of cells per image was normalized by dividing by the total area of that image. Then, the normalized values were added and divided by the number of images.

### ELISA

Blood samples were collected after 12 weeks of either HFHS or LFLS feeding. An additional group, only feeding with a HFHS diet, were treated with either celastrol or a vehicle solution for three consecutive days. Plasma concentrations of C-Peptide, ghrelin, glucagon-like peptide-1 (GLP-1), interleukin-6 (IL-6), glucagon, insulin, leptin, peptide YY (PYY) and tumour necrosis factor-α (TNFα) were measured using the MILLIPLEX Mouse Metabolic Hormone Expanded Panel kit (Millipore MMHMAG-44 K). For each animal, 0.5–0.8 mL of blood was collected by cardiac puncture while the animals were anesthetized. Following blood collection, all mice were euthanized under anaesthesia by cervical dislocation. The experimental procedures for sample collection, storage, preparation of reagents for immunoassay and immunoassay procedure were performed following the instructions of the MMHMAG-44 K mouse panel protocols.

To determine the concentrations of each hormone in the samples, we utilized standard curves fitted using a four-parameter logistic curve-fitting algorithm (O’Connell et al. 1993). Hormone levels were expressed in pg/mL. To assess the statistical variation among replicates, we utilized the coefficient of variation (%CV). The mean values were calculated only when the %CV was below 10%.

### Data analysis

All data and statistical analysis were performed using R (R Core Team 2021, 2025) and considering brain regions as biologically independent measures, and thus tested separately. BW and food intake changes during treatment were assessed building corresponding linear mixed effects (lme) models, with time (pre, 24 h, 48 h and 72 h), diet (HFHS or LFLS), sex (male or female) and type of treatment (vehicle or celastrol) as main predictors, and all interactions considered. This was achieved using the *lmer* function of the lme4 package (Bates et al. 2015), with subsequent Type II Wald chi-square tests and post-hoc contrasts using *emmeans* (Lenth 2017), and corrected for multiple comparisons by false discovery rate adjustment (FDR). MRI variables were analyzed in two steps. First, values from the pre-treatment were checked for corresponding BW, *diet*, *sex* and *diet:sex* interaction dependance, using a linear model with the *lm* function, followed Type-II or Type-III ANOVA tests (for non-significant or significant interactions, respectively). Next, for those MRI pre-treatment parameters that showed significant effects of either *diet* or *diet:sex*, further tests were performed on the *post-treatment* condition, adding to the linear model the type of treatment, the double *(treatment: diet*, *treatment:sex*) and triple (*treatment:diet:sex*) interactions as predictors. When significant interactions were found, post-hoc differences at each level with FDR correction were performed. IF-derived variables were subjected to lme fitting, for each region independently, with diet and treatment as main factors, its interaction, and “mouse” as a random term -since data from several quadrants was included-. To further associate the cellular morphological alterations with the type of diet or treatment received, IF variables were subjected to a principal component analysis (PCA) followed by a clustering procedure using R (see Supplementary material for more methodological details).

HRMAS data was subjected to a random forest (RF) analysis using the *randomForest* function from the randomForest package (Liaw and Wiener 2001), to discriminate groups based on the metabolite profile, with the *type* of treatment as the outcome, and all metabolite ratios to PCr + Cr as predictors (including all regions and TEs). Briefly, RF is a machine learning method that uses decision trees created by using bootstrap samples of a training data, and a random feature selection in tree induction (Breiman 2001). The procedure included five different steps, including (i): a repeated fivefold cross-validation (CV) scheme, where full dataset was randomly partitioned into five approximately equal folds. Four folds (80% of the data) were used to train the model, and the remaining fold (20%) served as the validation set; (ii): all possible training–testing combinations within the 5-folds were subjected to RF analysis. Using the *tuneRF* function from the randomForest package, each *mtry* (number of variables randomly sampled at each split) was tuned to select the *mtry* value that minimized the out of bag (OOB) error. For each *mtry*, a forest with 500 trees was grown, and the configuration associated with the lowest OOB error rate was retained, resulting in an optimum *mtry* = 15. The final RF for that repetition was then refitted on the entire dataset using the selected *mtry* and 500 trees, and the OOB error from this model was recorded; (iii): this whole procedure was repeated 20 times with different initial random partitions, yielding 100 train–validation cycles in total. Across the 20 repetitions we obtained a distribution of OOB error estimates, from which the *mean OOB error* and its *variability* was extracted as measures of predictive performance and stability, resulting in OBB_mean_ ± SD of 0.26 ± 0.01 of all iterations; (iv): for each of the 20 final RF models, we extracted the *Mean Decrease in Gini index* as a global indicator of how much each metabolite contributed to node purity and classification accuracy. This produced 20 separate *importance profiles*. To identify robust biomarkers, we analyzed the stability of the importance rankings across repetitions, and metabolites that were ranked in the top 10 in at least 75% of the repetitions (i.e. in ≥ 15 out of 20 runs) were considered *stable important metabolites*, that were retained further analysis; and (v): the final RF model, using the optimized *mtry*, was tested in a 70/30 (training/testing) split, and confusion matrix assessed. Finally, ANOVA of the stable important metabolites were performed to assess the classical statistical significance of type of treatment, and the effects on those metabolites of diet, sex, and corresponding interactions, with FDR corrections for post-hoc multiple comparisons. Off note, sex was initially considered as a potential main predictor in all fitted models and was removed from them if its contribution was not significant.

## Results

### Physiological changes

**Fig. 2:**
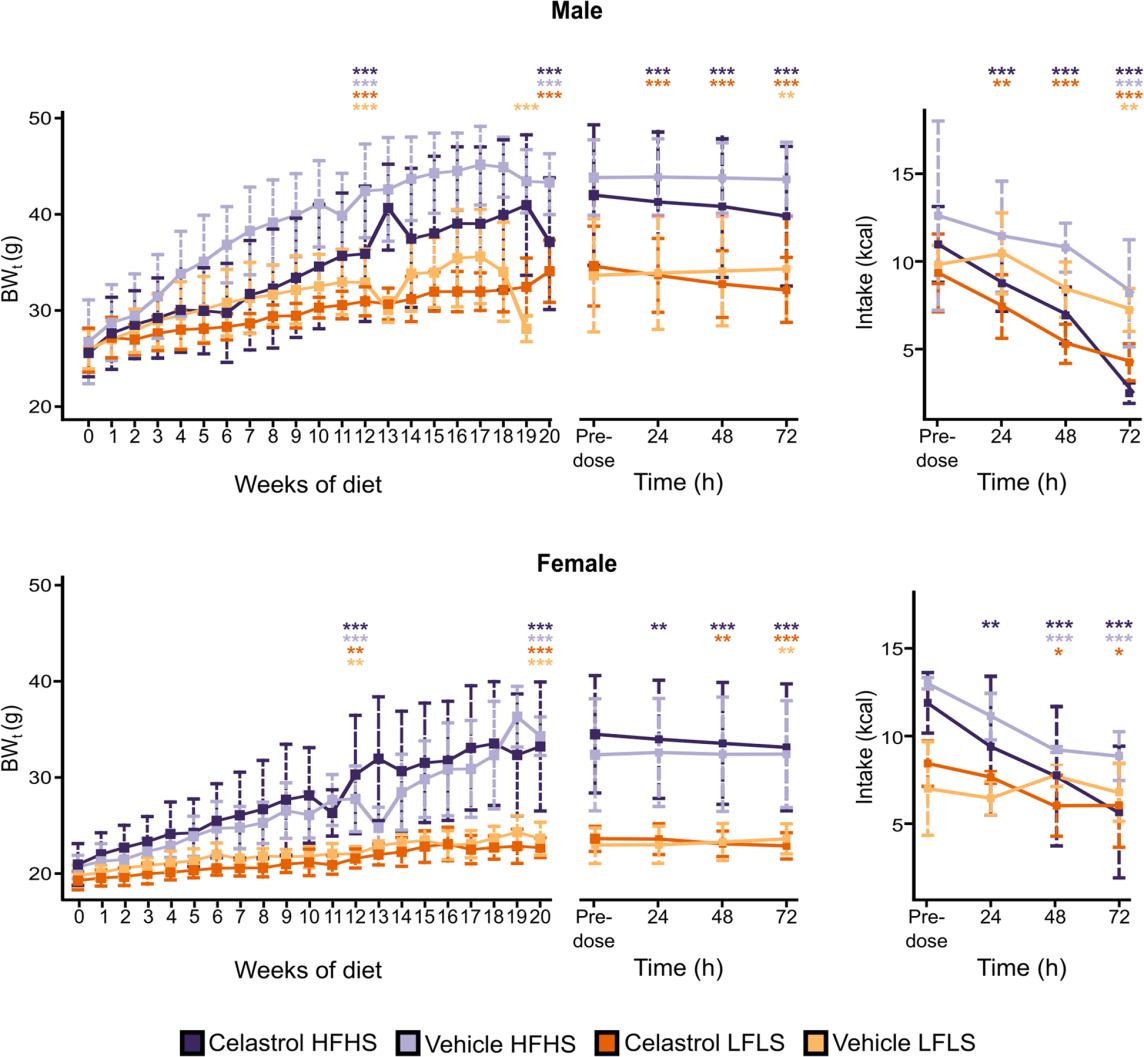
Physiological changes during the 20 weeks of diet and the following three-day treatment period with either celastrol or vehicle. Changes in BW (left) and food intake (right) during the 20 weeks of diet, at Pre-dose time point and at different time points after treatment initiation for males (top) and females (bottom), in both the HFHS (blue colors) and LFLS (orange) cohorts. Food intake was calculated as average values per cage. All values are expressed as mean ± standard deviation (*p_adj_ < 0.05, **p_adj_ < 0.01, ***p_adj_ < 0.001)

After 20 weeks of diet diversification, male and female animals fed with HFHS diet revealed higher BW and food intake values, as compared to LFLS, according to an obese phenotype (Fig. 2). BW measurements during treatment evolved differently depending on the type of diet consumed, treatment received and animal’s sex, as revealed by a significant *time:diet:treatment:sex* interaction on BW (χ = 20.3, df = 3, *p* < 0.001). To understand such differences, post-hoc time contrasts between the “Pre dose” level and the rest of the time points, per type of diet, treatment and sex, were assessed. Results indicated that HFHS and LFLS male animals administered with celastrol showed large BW decays with significantly reduced values from 24 h, while HFHS females started at 24 h, but LFLS females 48 h after the first dose, both with smaller decays that males (Fig. 1 top panels, Table 2). Animals receiving vehicle exhibited either no significant BW alterations or small increases, mainly in LFLD males (Table 2). Average intake per cage during the days of i.p. administration decreased very remarkably in all celastrol-batches, and vehicle administered showed either no changes (LFLS females) or delayed and fewer decreases, significant only for HFHS females from 48 h, and for males LFLS at 72 h (Fig. 1, bottom panels, Table 2).”

**Table 2:** Post-hoc tests after lme analysis of BW and intake changes with time. P-values are FDR adjusted

BW and Intake post-hoc tests	Contrast	BW DF	BW t-ratio	BW *p*-value	Intake DF	Intake t-ratio	Intake *p*-value	
HFHS Vehicle Females	12–0 weeks	916	8.7	< 0.0001				
	20–0 weeks	916	12.4	< 0.0001				
	24 h- Pre Dose	150	1.8	> 0.05	138	−0.4	> 0.05	
	48 h- Pre Dose	150	2.1	> 0.05	138	−1.5	> 0.05	
	72 h– Pre Dose	150	1.9	> 0.05	138	−3.0	0.0083	
HFHS Celastrol Females	12–0 weeks	916	11.5	< 0.0001				
	20–0 weeks	916	15	< 0.0001				
	24 h- Pre Dose	150	−2.9	0.0048	138	−4.0	0.0001	
	48 h- Pre Dose	150	−6.6	< 0.0001	138	−7.5	< 0.0001	
	72 h– Pre Dose	150	−9.8	< 0.0001	138	−10.8	< 0.0001	
LFLS Vehicle Females	12–0 weeks	916	2.7	< 0.0001				
	20–0 weeks	916	4.7	0.0070				
	24 h- Pre Dose	150	0.0	> 0.05	138	−1.4	> 0.05	
	48 h- Pre Dose	150	0.7	> 0.05	138	−2.3	> 0.05	
	72 h– Pre Dose	150	1.9	> 0.05	138	−0.3	> 0.05	
LFLS Celastrol Females	12–0 weeks	916	3.2	< 0.0001				
	20–0 weeks	916	4.3	0.0015				
	24 h- Pre Dose	150	−0.3	> 0.05	138	−0.3	> 0.05	
	48 h- Pre Dose	150	−3.2	0.0028	138	−4.3	< 0.0001	
	72 h– Pre Dose	150	−4.5	< 0.0001	138	−5.0	< 0.0001	
HFHS Vehicle Males	12–0 weeks	916	19.2	< 0.0001				
	20–0 weeks	916	18.8	< 0.0001				
	24 h- Pre Dose	150	1.1	> 0.05	138	−2.7	0.0069	
	48 h- Pre Dose	150	0.2	> 0.05	138	−4.2	0.0001	
	72 h– Pre Dose	150	0.2	> 0.05	138	−4.7	< 0.0001	
HFHS Celastrol Males	12–0 weeks	916	12.6	< 0.0001				
	20–0 weeks	916	14.2	< 0.0001				
	24 h- Pre Dose	150	−3.7	0.0003	138	−4.0	0.0001	
	48 h- Pre Dose	150	−6.2	< 0.0001	138	−6.8	< 0.0001	
	72 h– Pre Dose	150	−11.5	< 0.0001	138	−11.7	< 0.0001	
LFLS Vehicle Males	12–0 weeks	916	8.5	< 0.0001				
	19–0 weeks	916	4.6	< 0.0001				
	24 h- Pre Dose	150	1.3	> 0.05	138	0.6	> 0.05	
	48 h- Pre Dose	150	2.3	0.0312	138	−2.6	0.0166	
	72 h– Pre Dose	150	3.5	0.0016	138	−4.3	0.0001	
LFLS Celastrol Males	12–0 weeks	916	5.7	< 0.0001				
	20–0 weeks	916	8.6	< 0.0001				
	24 h- Pre Dose	150	−5.1	< 0.0001	138	−4.7	< 0.0001	
	48 h- Pre Dose	150	−9.8	< 0.0001	138	−7.5	< 0.0001	
	72 h– Pre Dose	150	−13.1	< 0.0001	138	−9.6	< 0.0001	

### HFHS diet and celastrol effects on brain MRI parameters

**Fig. 3:**
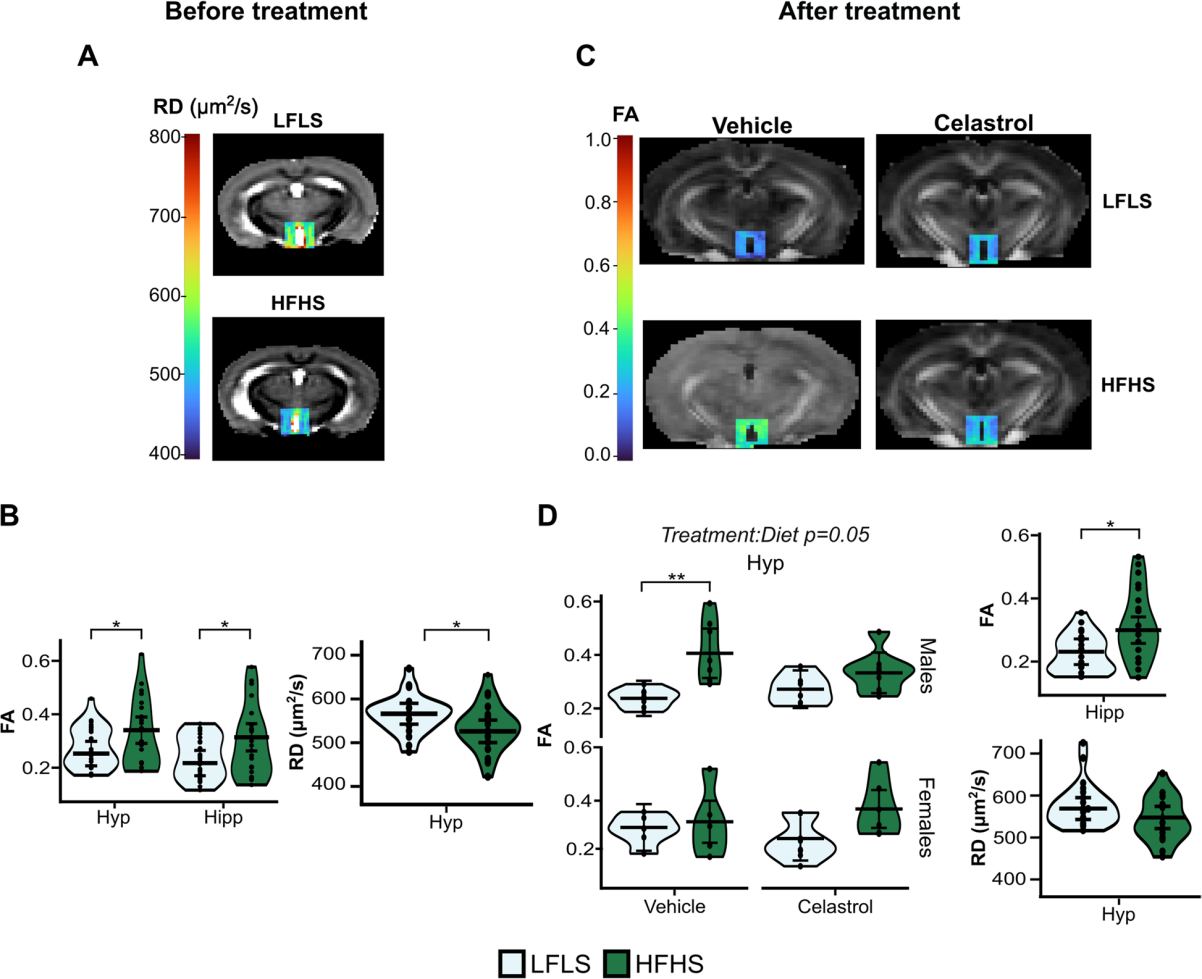
DTI brain changes with diet and treatment. **A**: Parametric maps of RD before treatment of the brain slice containing the hypothalamus of representative LFLS (top) and HFHS (bottom) mice. **B**: Violin plots plus values from individual animal quantification of the regional MRI parameters that showed significant differences between diet groups, namely FA in the Hyp and Hip (left panels) and RD in the Hyp (right panel), with HFHS mice depicted in green, and LFLS in light blue. Data include both sexes, as no significant sex effect was observed. The horizontal bars and confident intervals represent corresponding mean ± CI values of the linear model estimated by the *emmeans* function. **C**. Parametric maps of FA after treatment of the brain slice containing the hypothalamus of representative LFLS (top) and HFHS (bottom) mice, under vehicle (left) or celastrol (right) administration. **D**: Violin plots plus individual representation of the regional parameters that showed significant differences either between diet or treatment groups, including FA in the Hyp (left panels), FA in the Hipp (top right; including both sexes) and RD in the Hyp (bottom right; including both sexes). RD after treatment did not show further significances between diet groups. Results from HFHS mice are depicted in green, and LFLS in light blue. The horizontal bars and confident intervals represent the corresponding mean ± CI values of the linear model estimated by the *emmeans* function (*p_adj_ < 0.05, ** p_adj_ < 0.01, *** p_adj_ < 0.001)

Analysis of the brain DTI parameters of HFHS or LFLS mice, namely MD, AD, RD, FA and MTR, revealed significantly higher FA on obese mice, both in the Hyp (F = 5.2, *p* < 0.05), and Hipp (F = 6.1, *p* < 0.05), and lower RD in the Hyp (F = 4.1, *p* = 0.05) (Fig. 3B–C). After treatment, hypothalamic FA remained significantly higher on HFHS male mice when treated with vehicle, and not celastrol (significant *treatment:diet:sex* interaction, df = 1, F = 4.1, *p* = 0.05) and post-hoc significance on vehicle male HFHS Vs LFLS (df = 23, t = −3.2, p_adj_ < 0.05) (Fig. 3C and D, left panel), while RD showed no further differences between dies, regardless of treatment (Fig. 3D, bottom right). In the hippocampus, no treatment effect was reported, and FA values continued significantly elevated on obese mice (F = 4.3, *p* < 0.05) (Fig. 3D, top right).

### HFHS diet and celastrol effects on astrocytes and microglia

**Fig. 4:**
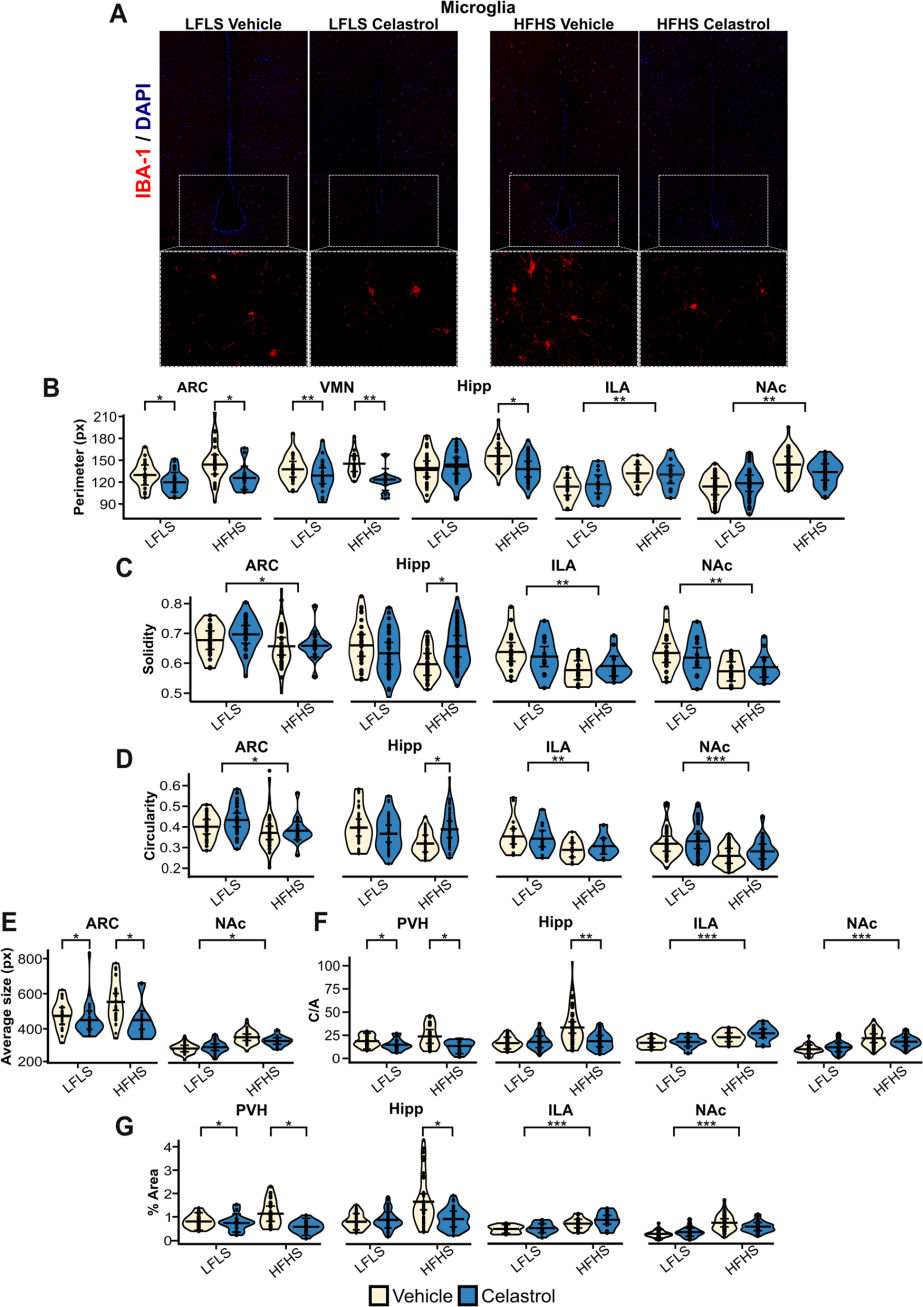
HFHS diet and celastrol effects on microglia. **A**: Representative immunofluorescence images of the Hyp, including ARC, PVH and VMN, from a LFLS vehicle and LFLS celastrol mice (left), and HFHS vehicle and HFHS celastrol mice (right) (top panels) and images of the ARC using a 40 × objective (bottom panels). IBA-1 staining (red) was used to label microglia, and cell nuclei were stained with DAPI (blue). **B-G**: Violin plots with superimposed individual data points, each representing the average values per quadrant, showing measurements of Perimeter, Solidity, Circularity, Average Size, counts per area (C/A), and percentage of occupied area (%A) from vehicle- (yellow) and celastrol-treated (blue) mice. Average Size and Perimeter are expressed in pixels (px). Data from both sexes are depicted together, as no significant sex effect was detected. The horizontal bars and confident intervals represent corresponding mean ± CI values of the linear model estimated by the *emmeans* function (*p_adj_ < 0.05, ** p_adj_ < 0.01, *** p_adj_ < 0.001)

IF images of the hypothalamic region of diet-induced obese mice showed enlarged and more abundant microglia, as compared to non-obese animals (Fig. 4A), with similar trends observed in the hippocampus IF images (supplementary material). The ILA and NAc regions were altered by diet, but celastrol did not withdraw such effects (Supplementary material). Statistical testing of the morphological descriptors perimeter, solidity, circularity, average size, %A and C/A yielded corresponding significant changes with diet and treatment, in several regions (Figs. 4B to G, Table 3). Results included consistent increases in microglial perimeters and sizes during HFHS feeding, that decreased after treatment, while circularity and solidity were decreased during obesity. The PCA + clustering analysis confirmed such behavior by identifying the HFHS vehicle group with maximum values of such variables, consistently across the hypothalamic nuclei and hippocampus (Supplementary material).

**Table 3:** Mean values of the descriptive morphological microglial parameters in the four experimental groups and statistical tests in all regions investigated (lme followed by wald tests)

Variable	Region	LFLS_Cel_	LFLS_Veh_	HFHS_Cel_	HFHS_Veh_	χ_diet_	χ_treat_	χ_inter_	p_diet_	p_treat_	p_inter_
Perimeter	ARC	120.30	130.72	125.56	146.07	2.83	4.91		0.09	0.03	
	PVN	148.11	134.36	139.24	145.76	0.09	0.00		> 0.05	> 0.05	
	VMN	129.03	137.82	122.25	145.62	0.23	6.22		> 0.05	0.01	
	Hipp	141.32	138.46	137.85	155.67	0.47	0.42	5.07	> 0.05	> 0.05	0.02
	ILA	116.05	110.99	128.24	131.15	8.78	0.02		0.003	> 0.05	
	NAc	115.39	114.15	133.21	143.59	21.76	0.76		0.000	> 0.05	
Solidity	ARC	0.70	0.68	0.66	0.65	4.07	0.67		0.044	> 0.05	
	PVN	0.62	0.62	0.63	0.61	0.12	0.13		> 0.05	> 0.05	
	VMN	0.65	0.62	0.67	0.61	0.02	3.71		> 0.05	0.05	
	Hipp	0.63	0.65	0.65	0.58	1.01	1.23	6.80	> 0.05	> 0.05	0.01
	ILA	0.62	0.64	0.59	0.57	10.01	0.00		0.002	> 0.05	
	NAc	0.60	0.59	0.56	0.53	9.17	0.80		0.002	> 0.05	
Circ	ARC	0.43	0.40	0.38	0.37	6.63	2.15		0.010	> 0.05	
	PVN	0.35	0.35	0.35	0.33	0.22	0.19		> 0.05	> 0.05	
	VMN	0.39	0.35	0.39	0.34	0.08	3.29		> 0.05	> 0.05	
	Hipp	0.36	0.38	0.38	0.31	0.66	1.19	6.84	> 0.05	> 0.05	0.01
	ILA	0.34	0.36	0.31	0.29	9.25	0.07		0.002	> 0.05	
	NAc	0.33	0.32	0.28	0.26	11.35	1.09		0.001	> 0.05	
Average Size	ARC	452.28	477.17	433.80	557.50	1.16	3.84		> 0.05	0.05	
	PVN	498.42	438.08	477.50	502.78	0.60	0.05		> 0.05	> 0.05	
	VMN	446.07	464.46	408.79	502.33	0.07	2.96		> 0.05	> 0.05	
	Hipp	480.96	480.51	488.30	491.81	0.13	0.00	0.01	> 0.05	> 0.05	> 0.05
	ILA	301.49	306.51	326.93	326.62	2.44	0.06		> 0.05	> 0.05	
	NAc	279.30	271.84	314.23	335.61	29.99	0.58		0.000	> 0.05	
C/A	ARC	16.93	19.72	20.73	31.81	1.87	2.38		> 0.05	> 0.05	
	PVN	14.91	18.58	12.81	23.61	0.34	4.08		> 0.05	0.04	
	VMN	14.67	16.77	11.86	23.67	0.44	2.87		> 0.05	0.09	
	Hipp	17.66	16.61	18.53	33.18	0.03	0.12	8.19	> 0.05	> 0.05	0.00
	ILA	16.39	15.15	24.61	20.92	12.52	1.62		0.000	> 0.05	
	NAc	12.14	9.80	17.95	21.75	15.97	0.13		0.000	> 0.05	
%A	ARC	0.77	0.95	0.88	1.84	1.77	2.96		> 0.05	> 0.05	
	PVN	0.76	0.81	0.58	1.14	0.41	3.93		> 0.05	0.04	
	VMN	0.65	0.76	0.48	1.20	0.68	3.49		> 0.05	> 0.05	
	Hipp	0.86	0.80	0.90	1.63	0.03	0.12	6.68	> 0.05	> 0.05	0.01
	ILA	0.51	0.46	0.83	0.69	12.16	1.44		0.000	> 0.05	
	NAc	0.35	0.27	0.58	0.74	18.20	0.30		0.000	> 0.05	

**Fig. 5:**
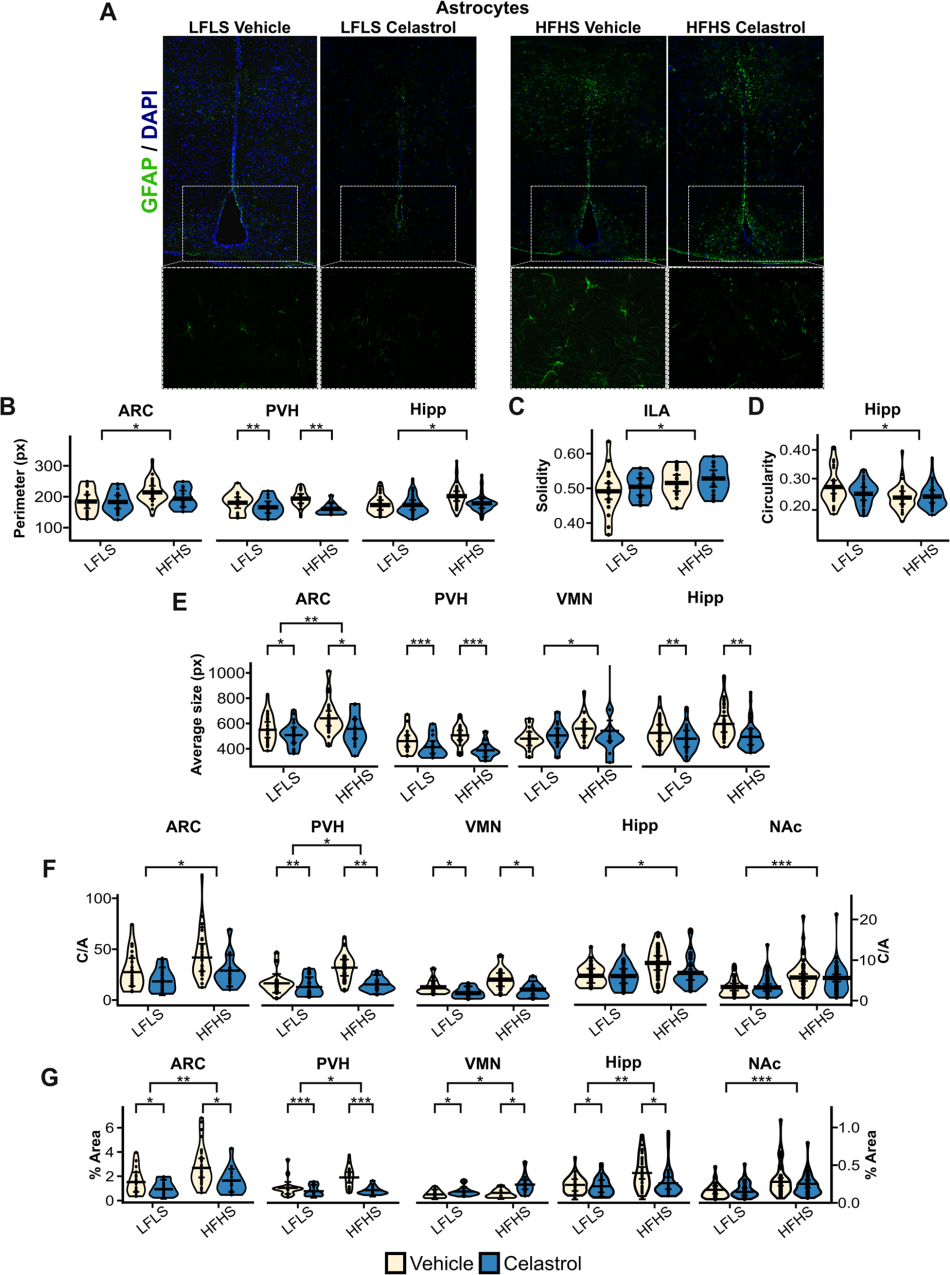
HFHS diet and celastrol effects on astrocytes. **A**: Representative immunofluorescence images of the Hyp, including ARC, PVH and VMN, from a LFLS vehicle and LFLS celastrol mice (left), and HFHS vehicle and HFHS celastrol mice (right) (top panels) and images of the ARC using a 40 × objective (bottom panels). GFAP staining (green) was used to label astrocytes, and cell nuclei were stained with DAPI (blue). **B-G**: Violin plots with superimposed individual data points, each representing the average values per quadrant, showing measurements of Perimeter, Solidity, Circularity, Average Size, counts per area (C/A), and percentage of occupied area (%A) from vehicle- (yellow) and celastrol-treated (blue) mice. Average Size and Perimeter are expressed in pixels (px). Data from both sexes are included in the same violin panels, since no significant sex effects were detected. The horizontal bars and confident intervals represent corresponding mean ± CI values of the linear model estimated by the *emmeans* function (*p_adj_ < 0.05, ** p_adj_ < 0.01, *** p_adj_ < 0.001)

Hypothalamic astrocytes showed enlarged perimeters and higher percentage of area occupied with HFHS, that tended to decrease after treatment (Fig. 5A), with comparable effects on the hippocampus (supplementary material). In the NAc and ILA, diet effects were present, but celastrol did not reverse them (supplementary material). Statistical testing confirmed such tendencies (Table 4, Figs. 5B-G) and PCA + clustering analysis pointed to high perimeter, high % of occupied area but low circularity and solidity as descriptors of the HFHS-vehicle clustering group in astrocytes (supplementary material).

**Table 4:** Mean values of the descriptive morphological astrocytes’ parameters in the four experimental groups and statistical tests in all regions investigated (lme followed by wald tests)

Variable	Region	LFLS_Cel_	LFLS_Veh_	HFHS_Cel_	HFHS_Veh_	χdiet	χ_treat_	p_diet_	P_treat_
Perimeter	ARC	183.45	185.42	193.45	214.65	4.39	1.00	0.04	0.32
	PVN	165.93	179.33	158.00	192.54	0.53	8.80	0.47	0.00
	VMN	179.42	173.51	182.91	197.72	2.89	0.08	0.09	0.77
	Hipp	169.53	172.68	177.12	200.36	4.87	2.31	0.03	0.13
	ILA	232.37	226.54	248.03	266.12	1.78	0.23	0.18	0.63
	NAc	184.34	169.19	161.25	177.67	0.23	0.00	0.63	0.97
Solidity	ARC	0.54	0.55	0.53	0.52	0.54	0.12	0.46	0.73
	PVN	0.52	0.51	0.52	0.50	0.40	1.08	0.53	0.30
	VMN	0.54	0.55	0.58	0.52	0.01	0.70	0.91	0.40
	Hipp	0.55	0.57	0.54	0.54	3.60	0.94	0.06	0.33
	ILA	0.50	0.49	0.53	0.52	3.90	1.10	0.05	0.29
	NAc	0.56	0.56	0.58	0.56	0.35	0.33	0.55	0.57
Circularity	ARC	0.24	0.25	0.23	0.22	1.69	0.02	0.19	0.88
	PVN	0.24	0.22	0.24	0.21	0.23	3.09	0.63	0.08
	VMN	0.25	0.25	0.26	0.22	1.06	0.75	0.30	0.39
	Hipp	0.25	0.27	0.24	0.23	4.88	0.82	0.03	0.36
	ILA	0.18	0.18	0.20	0.18	0.97	1.93	0.33	0.17
	NAc	0.21	0.21	0.24	0.22	1.36	0.48	0.24	0.49
Average.Size	ARC	499.12	543.63	541.56	633.33	6.51	4.54	0.01	0.03
	PVN	417.75	464.47	392.64	509.25	0.70	15.04	0.40	0.00
	VMN	489.43	464.13	525.57	542.28	5.56	0.12	0.02	0.73
	Hipp	470.90	524.93	493.57	597.94	2.10	6.46	0.15	0.01
	ILA	596.80	527.20	697.09	710.90	2.95	0.01	0.09	0.94
	NAc	408.28	390.54	381.55	429.07	0.06	0.18	0.81	0.67
C/A	ARC	19.29	27.65	27.29	42.89	4.09	3.06	0.04	0.08
	PVN	13.39	16.28	15.15	31.71	5.15	6.11	0.02	0.01
	VMN	7.50	12.86	8.80	20.38	3.52	5.82	0.06	0.02
	Hipp	22.47	23.78	26.51	35.98	5.53	2.43	0.02	0.12
	ILA	27.42	15.05	22.39	12.23	0.52	3.65	0.47	0.06
	NAc	3.61	3.02	5.25	6.08	25.34	0.07	0.00	0.79
%A	ARC	0.98	1.54	1.54	2.75	6.76	4.62	0.01	0.03
	PVN	0.57	0.81	0.61	1.67	4.82	8.10	0.03	0.00
	VMN	0.38	0.60	0.42	1.15	3.98	4.86	0.05	0.03
	Hipp	1.11	1.28	1.41	2.23	6.59	4.26	0.01	0.04
	ILA	1.72	0.92	1.65	0.99	0.06	2.17	0.81	0.14
	NAc	0.14	0.11	0.19	0.27	15.15	0.99	0.00	0.32

### HFHS diet and celastrol effects on blood plasma

**Fig. 6:**
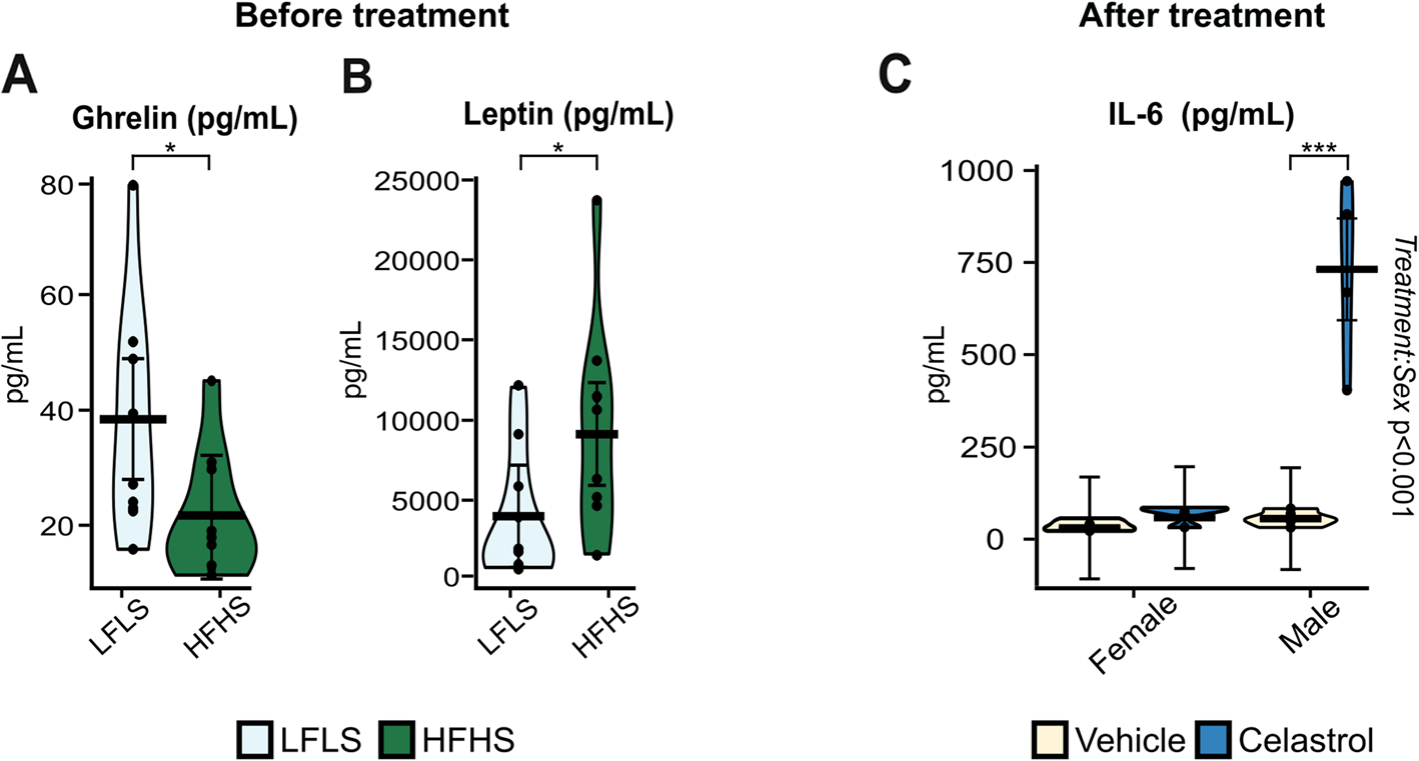
Hormonal changes. **A**: Ghrelin and **B**: Leptin violin plots are superposed to experimental concentrations (dots) and mean ± CI values of the linear model estimated by the *emmeans* function, of HFHS and LFLS mice before any treatment. Only these two hormones showed significant differences with diet. **C**: Values of IL-6 measured after treatment, in female and male vehicle or Celastrol-treated mice. No other hormone or cytokine show statistical differences between treatment. Panel A and B include values from both male and female mice (*p_adj_ < 0.05, ** p_adj_ < 0.01, *** p_adj_ < 0.001)

After 12 weeks of diet diversification, LFLS and HFHS mice that had reached an obese phenotype (41.5 ± 3.6 g HFHS Vs. 32.1 ± 3.2 g LFLS males and 27.1 ± 3.6 g HFHS Vs. 19.9 ± 0.6 LFLS females) were used for analysis of blood plasma. HFHS mice showed lower ghrelin and higher leptin levels, in comparison to LFLS group (df = 1, F_ghrelin_ = 5.7, p_ghrelin_ < 0.05, F_leptin_ = 5.8, p_leptin_ < 0.05) Fig. 6A). The rest of the hormones measured showed high variability between animals, and no other relevant effects between diet batches were found (Table 5). To evaluate the effects of celastrol on serum levels, we compared samples from HFHS mice treated with either celastrol or vehicle. The analysis revealed a significant interaction *Sex:Treatment* (F = 27.0, *p* < 0.001) on circulating values of IL-6, revealing that big changes were occurring in males (*post-hoc* Celastrol Vs Vehicle Males df = 12, t = 7.6, p_adj_ < 0.001) (Fig. 6B).

**Table 5:** Plasma values after either HFHS or LFLS diet consumption, and HFHS values after treatment with celastrol or vehicle

Concentration (pg/mL)/Diet and condition	HFHS		LFLS		HFHS-vehicle		HFHS-celastrol	
	Male	Females	Male	Females	Male	Females	Male	Females
Ghrelin	29.18 ± 10.98	15.12 ± 3.08	36.74 ± 25.23	40.98 ± 14.30	15.96 ± 4.17	31.43 ± 15.54	23.12 ± 13.74	21.47 ± 3.57
Glucagon	77.17 ± 74.45	31.45 ± 21.09	76.41 ± 22.32	89.47 ± 47.63	19.94 ± 6.23	57.96 ± 35.79	25.88 ± 7.40	18.03 ± 3.27
PYY	146.31 ± 32.51	101.01 ± 35.25	155.99 ± 50.01	128.50 ± 47.37	110.58 ± 90.32	85.14 ± 28.06	141.07 ± 63.01	122.80 ± 16.87

### Cerebral metabolic changes of celastrol by HRMAS

**Fig. 7:**
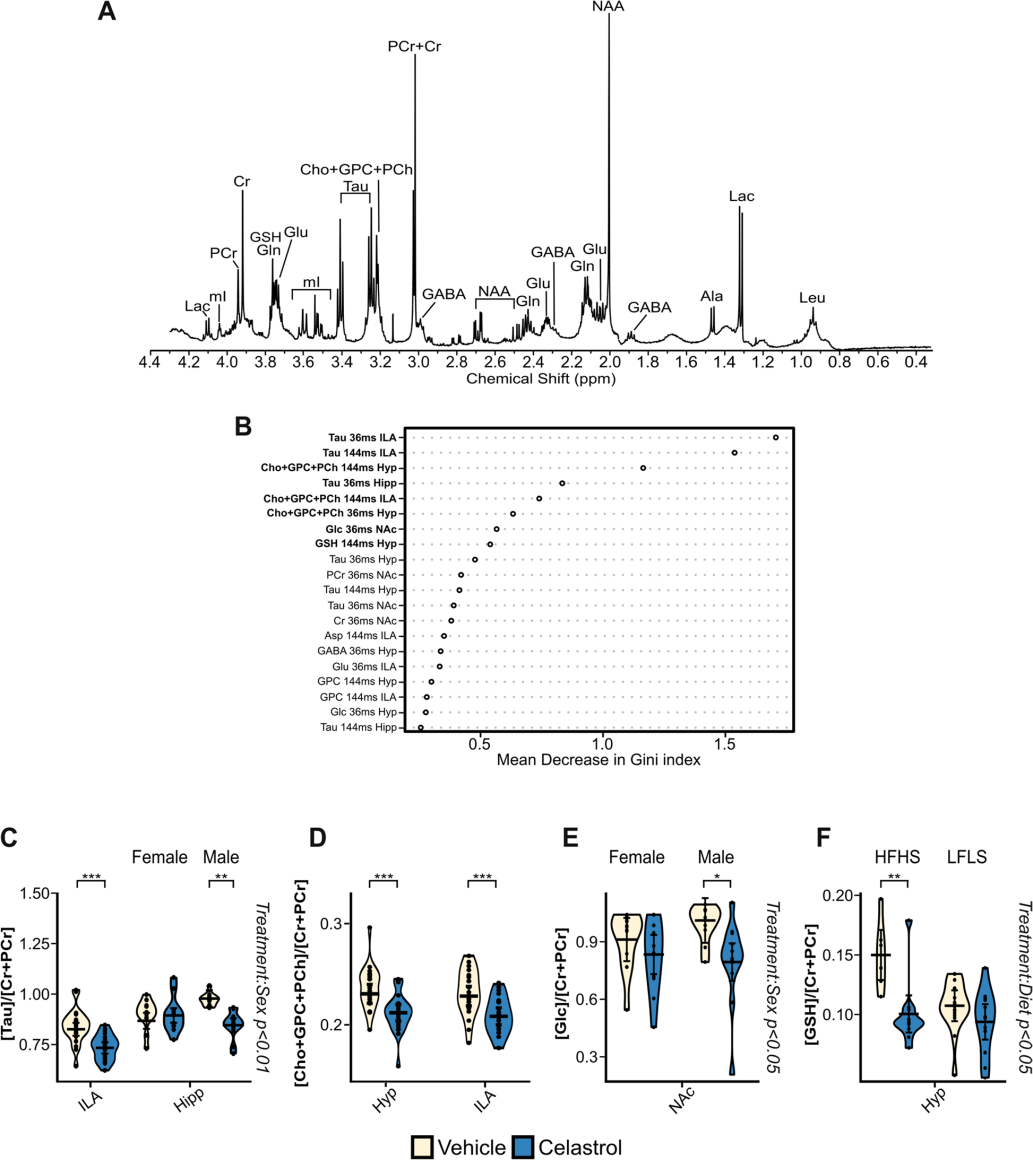
Ex-vivo HRMAS Spectroscopy Post-Treatment. **A**: Representative.^1^H HRMAS spectrum of ILA. **B**: Mean Decrease Gini values of the metabolites exhibiting the highest importance scores in the random forest analysis. **C-F**: Boxplots representing the concentrations of metabolites normalized to total creatine content (Cr + PCr), comparing the vehicle and celastrol groups. **C**: Tau. **D**: Cho + GPC + PCh. **E**: Glc, **F**: GSH. Panel C, D and F include values from both male and female mice since sex effect was not significant (*p_adj_ < 0.05, ** p_adj_ < 0.01, *** p_adj_ < 0.001)

HRMAS acquisition of the cerebral samples resulted in very good quality spectra in all regions and animals, with a signal-to-noise ratio ranging from 17 to 26 and full width at half maximum linewidths around 2.5 Hz (Fig. 7A). Subsequent fitting of the spectra to the metabolic database yielded a good adjustment, and LCModel provided the relative concentration of metabolites for each sample. Amongst the derived *stable important metabolites* delivered by the RF analysis, we found metabolites from all regions, with the sum of Cho + GPC + PCh in the Hyp, and Tau in the Hipp and ILA appearing in the 20 repetitions as amongst the top-10 metabolites having the higher GiniInx, and GSH in the Hyp, Cho + GPC + PCh in the ILA, Glc in NAc and Tau in the Hyp appearing in between 15 and 19 repetitions. A representative *mean decreases in Gini index* plot of metabolites is shown in Fig. 7B. The final RF was applied to a 70/30 training/testing partition, yielding a confusion matrix predicting 6/8 subjects to the celastrol group (2/6 incorrectly assigned to vehicle), and 4/4 from the vehicle group correctly allocated (0.83 accuracy).

ANOVA tests of the stable important metabolites yielded significant effects of either type, or interactions *type:diet*, *type:sex*, in all metabolites except on hypothalamic Tau (Table 6). Notably, Tau ratios to PCr + Cr were reduced in the celastrol-treated group compared to the vehicle group in both the ILA (Fig. 7C, Table 6). Similarly, Cho + GPC + PCh ratio to PCr + Cr was significantly elevated in the ILA and Hyp (both TE) (Fig. 7D and Table 6). Additionally, the celastrol-treated male mice showed decreased Glc ratios than the vehicle-administered male animals (Fig. 7E). HFHS-celastrol group exhibited GSH/PCr + Cr lower than the HFHS-vehicle animals, with values after treatment reaching similar levels of LFLS (Fig. 7F).

**Table 6:** Main effects and interactions of type of treatment, sex and diet, on stable important metabolites TE, comparing the vehicle and celastrol groups. C: Tau. D: Cho + GPC + PCh. E: Glc, F: GSH. (*padj < 0.05, ** padj < 0.01, *** padj < 0.001)

Effect/Metabolite	Cho + GPC + PCh Hyp		Tau ILA		Tau Hipp	GSH Hyp	Cho + GPC + PCh ILA	Glc NAc
	**TE = 144**	**TE = 36**	**TE = 144**	**TE = 36**	**TE = 36**	**TE = 144**	**TE = 144**	**TE = 36**
Type	F = 13.7, *p* < 0.001	F = 5.3, *p* < 0.05	F = 23.0, *p* < 0.001				F = 13.6, *p* < 0.001	
Type:Sex					F = 8.6, *p* < 0.01			F = 4.1, *p* < 0.05
*Post-hoc Celastrol Vs Vehicle males*					*df* = *37, t* = *−5, p* < *0.001*			*df* = *37, t* = *−2.9, p* < *0.05*
Type:Diet				F = 4.2, *p* < 0.05		F = 4.9, *p* < 0.05		
*Post-hoc Celastrol Vs Vehicle*				df = 37, t = −4.8, *p* < 0.001 (LFLS)		df = 37, t = −3.8, *p* = 0.001 (HFHS)		

## Discussion

In this work, we have described the effects of celastrol as an anti-obesity and anti-inflammatory agent on an animal model of diet-induced obesity. First, and prior to any treatment, we characterized obesity development by comparing BW, food consumption, in vivo MRI markers of neuroinflammation and systemic blood hormonal changes. Results indicate that the obese phenotype is characterized by an inflammatory state in the brain, which can be quantified in vivo by increased FA and reduced RD. Next, we administered acute treatments with either celastrol or its vehicle (DMSO) and observed, first, generalized decreases in food intake but celastrol-specific BW decreases. Thereafter, we could quantify that the obesity-induced alterations were modified by celastrol, including decreases on the in vivo FA values, particularly in the Hyp, paralleled by reduction of microglial and astrocytic perimeters, number and area, as well as augmented cellular circularity and solidity, revealing anti-inflammatory mechanisms of the tested compound. Additionally, the cerebral metabolic changes induced by celastrol suggest the regularization of osmolytes and of cellular proliferation markers, and systemic blood changes that reveal major changes on an anti-inflammatory cytokine.

### HFHS diet increases FA regionally and celastrol reverts changes

Among MRI methods, DTI has been instrumental in detecting microstructural changes in both grey and white matter across various neurodegenerative and pathological conditions, in humans as well as in animal models. The biological interpretation of changes in diffusion coefficients requires, however, several considerations, including the tissue composition of the region investigated. Indeed, alterations in white or grey matter induce distinct diffusion patterns, and, vice versa, a comparable variation of a diffusion coefficient may arise from diverse biological responses. For instance, as white matter is composed mainly of neuronal bundles, which favor diffusion along its parallel direction, an integrity loss of the fibers would yield decreased FA values, as it has been reported in the corpus callosum of subjects with obesity (Daoust et al. 2021). At the same time, in grey matter, gliosis –a state with increased cellularity and larger and irregular cellular bodies- has been related to increased FA (Guadilla et. al 2021; Lizarbe et al. 2020b). In subcortical areas, where grey matter is also predominant, another study found decreased MD, AD and FA with body mass index in young patients with obesity, but increased FA in older patients (Tweedale et al. 2024). In their work, authors interpreted the decreased diffusivity coefficients as potentially reflecting gliosis and proposed to link the higher FA to an anisotropic inflammatory process, as it was positively associated with C-reactive protein values. In our study, we report that animals fed with a HFHS diet show increased FA and decreased RD in grey matter regions, significantly in the Hyp and Hipp, which agrees well with increases in cellularity and a gliosis-associated anisotropic change of cellular shape, and, thus, inflammation, in line with the previous studies. Notably, our MRI in vivo results are supported by immunofluorescence images showing morphological changes consistent with microgliosis and astrocytosis on HFHS brains. Moreover, such cellular changes were assessed not only visually, but also as statistically relevant increased astrocytic and microglial perimeters and lower circularity and solidity. Such geometrical characteristics compare well with a diffusion in the extracellular space that is restricted by the anisotropic perimetral growth of the cellular bodies composing the Hyp and Hipp.

After the anti-obesity treatment, we could find, in the Hyp, that differences between FA of HFHS or LFLS mice where only maintained in male vehicle-administered animals; in other words, that animals treated with celastrol showed no-longer-increased FA values in the Hyp. Besides, in this region, after treatment, RD was no longer different between diet groups, but no specific treatment-effect could be robustly statistically reported. In the Hipp, FA remained elevated in HFHS after 3 days of administration, as compared to LFLS, independent of the type of treatment. Results in the Hyp are supported by the immunofluorescence images, where we found significantly reduced perimeters, average sizes and percentage of occupation, and increased solidity after treatment, comparing well with an increase of FA, thus with water molecules diffusion in a less restricted extracellular space. Thus, results are consistent with an anti-inflammatory action of celastrol, detected in the Hyp, that can be quantified in vivo by an FA reduction and supported by immunofluorescence images.

### Microglia reacts first

Quantification of immunofluorescence images from IBA1 and GFAP markers showed that HFHS consumption induced very remarkable effects on morphological descriptors, revealing an extended effect of HFHS consumption on brain cells. In both cell types, caloric diet resulted in augmented perimeters, increased average sizes, C/A and %A, but reduced solidities and circularity. Changes were consistent across all areas, with less prominent changes on astrocytes in the ILA and NAc regions, as can be seen in Figs. 4–5. After treatment, HFHS animals administered with celastrol depicted a robust change in microglial cells in the Hyp and Hipp, as quantified by decreased perimeters, recovery in solidity (in the VMN), diminished average sizes and cell counts or %A. Notably, while geometrical descriptors changed more remarkably in microglia, changes in number and %A were more significant on astrocytes. ILA and NAc showed a sparse response to treatment. Notably, our PCA + clustering analysis identified clearly HFHS vehicle groups, amongst the four experimental groups, in the hypothalamic nuclei and hippocampus, as the cluster with higher perimeters or %of occupied area, but lower solidity and circularly, paralleling the results from statistical tests of the variables. In the NAc and ILA, data could be clustered into a HFHS group, not specifically HFHS-vehicle group, suggesting that diet was still the main effect amongst the 4 experimental groups, independent of treatment. Interestingly, in such analysis we could detect how perimeter and %A or average size were typically aligned in one principal component, while solidity and circularity governed the perpendicular component. Our reported microglial and astrocytic changes are on agreement with previous results of our group (Lizarbe et al. 2019) and others (García-Cáceres et al. 2019b), and have been consistently linked to a the diet-induced inflammatory process (Valdearcos et al. 2017b). Indeed, neuroinflammation is characterized by cellular proliferation and by an enhanced proportion of type M1 microglia, which have amoeboid shapes, and thus bigger cellular bodies that yield higher % of occupied area values and perimeters, than the non-pro-inflammatory ramified M2-type (Tam and Ma 2014). Moreover, this compares well with our reported increased reduced circularity and solidity—solidity is calculated as “Area/Convex area”, and *Convex area* represents the smallest convex shape that fully contains the particle, thus lower solidity = more irregular/indented processes or contours-. Celastrol-induced reversal of the microgliosis and astrocytosis is in line with the response to other anti-obesity treatments, which withdraw the pathological changes of microglial activation (Berkseth et al. 2014; Marinho et al. 2026; Rong et al. 2025). Particularly, the reduced % of area occupied by cells, reduced perimeter and increased solidity are on agreement with a switch from M1 -amoeboid- to M2 -ramified- microglial types, similar to findings after other pharmacological interventions (Wang et al. 2024), and seem to happen more consistently in microglia. Indeed, even though hypothalamic astrocytes are known to become reactive in DIO, most studies find that microglial activation is earlier and more robustly quantifiable than astrocytosis (Jais and Brüning 2017), and that microglial depletion or inhibition reduces subsequent astrogliosis, supporting the idea that astrocyte activation is at least partly downstream of microglial signaling (André et al. 2016). On the other hand, our results indicating that the number of cells decreased regionally after treatment may suggest that celastrol induced tissue reorganization and normalization, which has been reported in other types of insults. For example, studies assessing proliferation of microglia after stroke demonstrated that microglial cells tended to migrate to the lesion focus after an insult, adopting hypertrophic shapes, while that during recovery tend to disaggregate and recover more ramified and random shapes (Kikhia et al. 2025). Thus, the reduction of Iba1 + microglial and GFAP + astrocytes number detected after celastrol treatment could reflect both a reduction in activated subpopulations and a spatial redistribution with a phenotype switch to a less inflamed state, that is more difficult to detect with our counting strategies, rather than a global decrease in total cell number. Finally, the fact that changes were much more robustly quantifiable in the Hyp and Hipp reinforce the hypothesis of both areas being are primary responders of high-fat diet consumption, and treatment (Plantera et al. 2025).

### Effects of celastrol on brain metabolism

The effects of celastrol in cerebral metabolism were assessed by HRMAS, by comparing the neurochemical profile from celastrol-treated or vehicle-administered brain region samples. A random forest analysis revealed that the sum of the choline-containing compounds, Cho + GPC + PCh, and Tau were the metabolites that best classified the animals depending on their treatment, in the Hyp, ILA and Hipp, to a lesser extent, with the two metabolites being reduced in celastrol-treated mice.

Choline-containing compounds are known to be MRS markers of membrane turnover and cellular proliferation (Duarte et al. 2012). In the context of obesity, increased Cho concentrations are thought to be linked to chronic low-grade neuroinflammation and glial activation, consistent with existence of inflammatory signaling within the central nervous system (Vuković et al. 2026). The fact that Cho compounds are within the metabolites that best classify between treated and non-treated animals, and that their value is significantly lower in the celastrol group, agrees well with a decreased inflammatory state of the treated mice, as compared to non-treated. On the other hand, previous works on DIO have quantified increases of Tau in the Hyp, Hipp and cortex, and such rise has been proposed to represent a compensatory neuroprotective response to metabolic stress (Duarte et al. 2012; Lizarbe et al. 2018). Moreover, Tau supplementation is known to reduce body weight on HFD animals (Figueroa et al. 2016), via anti-inflammatory effects (Ahmed et al. 2024) and it has been shown to accumulate in the Hipp of DIO animals acting as a counteracting beneficial response to metabolic stress (Garcia-Serrano et al. 2023). Thus, the lower values reported here in treated animals are consistent with a celastrol-induced anti-inflammatory response that does no further need such metabolic stress-induced elevation of Tau levels. Other than Tau and the Cho compounds, GSH and Glc in certain brain regions were also amongst the top-10 metabolites with higher classification indexes. GSH is the main small‑molecule antioxidant in brain cells, and impairment of its function is linked as the result of neurological diseases, or during aging (Iskusnykh et al. 2022). Astrocytes contain substantially higher GSH than neurons, and changes in cerebral GSH in some human diseases are thought to be, at least in part, the consequence of alterations of astrocytic GSH, and/or of changes in the GSH metabolism of astrocytes (Dringen and Arend 2025). Indeed, during inflammation, astrocytes experience increased oxidative and inflammatory load, which typically drives upregulation of antioxidant systems, including the GSH pathway, to protect both themselves and neighboring neurons. In this sense, our results showing higher GSH levels in the hypothalamus un HFHS mice treated with vehicle, as compared to HFHS celastrol animals, which reach similar levels than LFLS mice, are on agreement with an obesity-induced GSH increase that can be reverted with celastrol. Glc, on the other hand, as measured in the NAc, appeared also as a stable important metabolite distinguishing between classes, depicting significantly lower values in treated male mice, as compared to non-treated. A decrease in Glc values after celastrol administration fits well with a switch from an obesity-associated hypermetabolic brain towards a more normalized glucose handling, and is on agreement with other anti-obesity treatments (Sewaybricker and Schur 2021).

Overall, the HRMAS measurements and analysis agree well with celastrol-induced cerebral metabolic functional rearrangements that reduce the inflammatory and metabolic stress associated with obesity.

### Systemic effects of celastrol

Serum analysis of blood hormones from obese and non-obese mice showed a *diet* effect on the concentrations of ghrelin and leptin (*p* < 0.05) that matched the expected hypothesis -decreased ghrelin during HFHS (Briggs et al. 2010), increased leptin-(Frederich et al. 1995). In HSHS mice treated with celastrol, IL-6 retained the *p* < 0.05 threshold, with obese male animals treated much larger values than vehicle-only, and no other effects could be reported.. IL-6 is a cytokine that plays a pivotal role in inflammatory responses, with diverse effects on regulating the immune response and metabolism via different signaling pathways, and it is known to particularly play an anti-inflammatory role in DIO related inflammatory diseases (Yang et al. 2025). For example, several cytokines, including IL-6, have been shown to reduce food intake when acting in the brain (Timper et al. 2017), and blockade of hypothalamic inflammatory signaling can increase susceptibility to DIO (Mishra et al. 2019; Wallenius et al. 2002). Additionally, GLP-1 suppression of food intake and BW is known to be mediated by Il-1 and IL-6 (Shirazi et al. 2013). Our results, together with the fact that IL-1 receptor is required for the effects of celastrol in DIO mice (Feng et al. 2019), support a model in which celastrol engages specific cytokine pathways, and we believe the increase in circulating IL-6 observed may reflect that it potentially mediates celastrol’s action. Notably, these effects could only be reported in male animals. Interestingly, this agrees well with males showing larger BW decreases with celastrol than females, and with the Hyp FA recovery on this specific group. Future studies, however, should consider potentially increasing sample sizes, as well as controlling feeding conditions prior to the blood extraction, to increase the statistical power of these tests and confirm the sexual-differences reported in the IL-6 measurements, together with testing other inflammatory cytokines such as IL-1 and TNFα.

## Conclusions

By using a wide range of methodological assessments, we have covered the physiological, systemic and central effects of celastrol administration to DIO and lean animals, and can conclude that it serves as an anti-obesity drug via anti-inflammatory mechanisms in the hypothalamus that can be detected in vivo by DTI, and ex vivo by histological, hormonal and cerebral metabolic changes. We believe that our results may prompt further interest in using celastrol as an anti-inflammatory agent with functional cerebral metabolic changes, during obesity and other pathologies involving inflammation, and that MRI methods could be used as in vivo biomarkers.

### Limitations

Our study investigates the anti-inflammatory and anti-obesity effects of celastrol during an acute administration. Interestingly, with only three doses, very high BW decreases were achieved, and indicators of both central and systemic anti-inflammatory and metabolic changes were reported. Future studies should assess a longer administration period, like treatments on human population, potentially using lower dosages, and determine the corresponding effects. Moreover, further research would be needed to understand the extent to which an acute administration of celastrol improves not only the inflammatory profile, but that genuinely improves glucose homeostasis and insulin sensitivity, by assessing corresponding glucose and insulin tolerance tests, and providing an integrative view of the treatment-associated changes in energy metabolism.

In our in vivo MRI study, even DTI reported consistent changes with *diet* and *treatment*, effect sizes of the corresponding parameters were lower than those observed in the ex vivo images by immunofluorescence of Iba1 and GFAP. Moreover, DTI could only quantify treatment response in the Hyp, while immunofluorescence images of Iba1, the microglial marker, detected changes both in the Hyp and Hipp. From one hand, and in order to provide more specificity regarding the compartmentalization of cellular changes, we suspect that the implementation of microstructural multicompartment tissue biophysical models (Garcia-Hernandez et al. 2022) could likely detect stronger effects on the corresponding diffusion parameters, and future studies should address this with more advanced acquisition strategies and biophysical models. On the other hand, however, this may also be reflecting stronger effects on the Hyp than in the Hipp, which is consistent with previous reports, which propose hypothalamic microglia as first responders for energy balance, while hippocampal microglia mediate longer-term cognitive consequences of metabolic stress (Plantera et al. 2025).

In this study, we could not detect any changes on MTR values, neither with the *diet* nor with *treatment* effects, although previous studies did (Campillo et al. 2022). What the mentioned study reported, however, were MTR values that increased in a within-subject manner during obesity development, changes that lean animals did not exhibit, but no direct comparison between obese and non-obese group was assessed. In this sense, in the present work, potential between-subjected variability may have precluded the finding of relevant differences between groups.

Another limitation of our study involves the high variability of the serum hormone results, which may have precluded the finding of some expected relevant effects, widely described in obesity. For example, values of the proinflammatory cytokine TNFα, which are known to increase during obesity (Makki et al. 2013), showed, in our study, high variability within animal groups, and no robust trends could be found. Indeed, many of the hormones studied here are dependent on feeding conditions, circadian rhythms and metabolic context (Lages et al. 2026), in general. Future studies aiming at disentangling the plasma inflammatory profile of obesity and anti-obesity treatment could improve such assessment by standardizing the measurements and performing blood extracting after a fasting period. On the other hand, potential cell culture experiments with microglia treated with celastrol could help unravelling the mechanistic by which microglia are deactivated, such as resolution of ER stress (Liu et al. 2015).

Finally, we would like to remark that, in this work, we assessed the cerebral effects of celastrol in pair-fed animal groups, but not in BW loss-matched controls. Celastrol or its vehicle (DMSO) were administered to 4 animal groups, including female and male animals, obese (HFHS-fed) and lean (LFLS-administered) mice (thus a total of 8 groups). During i.p. administration, all males decreased intake independently of the diet administered and treatment received, while all female groups decreased intake, except the LFLS-vehicle cohort. BW, on the contrary, decreased only in celastrol-administered animals, and increased (or was stable) in all vehicle-administered mice. On these grounds, we believe that the anti-inflammatory and metabolic rearrangements reported here are associated with celastrol treatment, and not to changes in intake. Moreover, the specific effect of BW values on the MRI variables was statistically tested, and no significant dependance could be reported, reinforcing the hypothesis of the treatment-associated changes. Further studies could compare brain changes induced by celastrol to other BW-loss approaches, including mediation and causal analysis between BW and the rest of the variables, to further understand such relationship.

## Supplementary Information

Supplementary Material 1.

Supplementary Material 2.

## Data Availability

The datasets generated and/or analysed during the current study are not publicly available due the need for a formal data sharing agreement but are available from the corresponding author on reasonable request.

## Abbreviations

AD: Axial diffusivity
Ala: Alanine
ANOVA: Analysis of variance
ARC: Arcuate nucleus
ASCM: Adaptive soft coefficient matching
Asp: Aspartate
Av: Number of averages
b: Diffusion weighting factor
BW: Body weight
Cho: Choline
Cr: Creatine
CSF: Cerebrospinal fluid
CV: Coefficient of variation
DIO: Diet-induced obesity
DMSO: Dimethyl sulfoxide
DTI: Diffusion tensor imaging
dMRI: Diffusion magnetic resonance imaging
ELISA: Enzyme-linked immunosorbent assay
ER: Endoplasmic reticulum
FA: Fractional anisotropy
FDR: False discovery rate
FOV: Field of view
GABA: Gamma-aminobutyric acid
GFAP: Glial fibrillary acidic protein
GLP-1: Glucagon-like peptide-1
Glc: Glucose
Gln: Glutamine
Glu: Glutamate
Gly: Glycine
GPC: Glycerylphosphorylcholine
GSH: Glutathione
HF: High-fat
HFD: High-fat diet
HFHS: High-fat high-sugar
Hipp: Hippocampus
HRMAS: High-resolution magic angle spinning
^1^H HRMAS: Proton high-resolution magic angle spinning
Hyp: Hypothalamus
IBA1: Ionized calcium-binding adaptor molecule 1
i.p.: Intraperitoneal
IL-6: Interleukin 6
ILA: Infralimbic area
Lac: Lactate
Leu: Leucine
LFLS: Low-fat low-sugar
Lip: Lipids
lme: Linear mixed-effects model
MC: Mesocorticolimbic complex
MD: Mean diffusivity
mI: Myo-inositol
MM: Macromolecules
MRI: Magnetic resonance imaging
MRS: Magnetic resonance spectroscopy
MTR: Magnetization transfer ratio
MTI: Magnetization transfer imaging
NAAG: N-acetylaspartylglutamate
NAc: Nucleus accumbens
NAA: N-acetyl-aspartate
OCT: Optimal cutting temperature compound
OOB: Out-of-bag
PBS: Phosphate-buffered saline
PCh: Phosphocholine
PCr: Phosphocreatine
PFA: Paraformaldehyde
Phe: Phenylalanine
PVN: Paraventricular nucleus
PYY: Peptide YY
RD: Radial diffusivity
RC: Reward centers
RF: Random forest
ROI: Region of interest
SD: Standard deviation
SFAs: Saturated fatty acids
Tau: Taurine
TE: Echo time
TNFα: Tumor necrosis factor alpha
TR: Repetition time
VMN: Ventromedial nucleus
