# Supplementary figures and images for: Acute anti-obesity treatment with celastrol reduces body weight, cerebral inflammation and metabolic imbalances in mice
Source: Mol Med. 2026 Jul 1;32:111. doi: 10.1186/s10020-026-01530-4 (PMC13366973; doi:10.1186/s10020-026-01530-4)

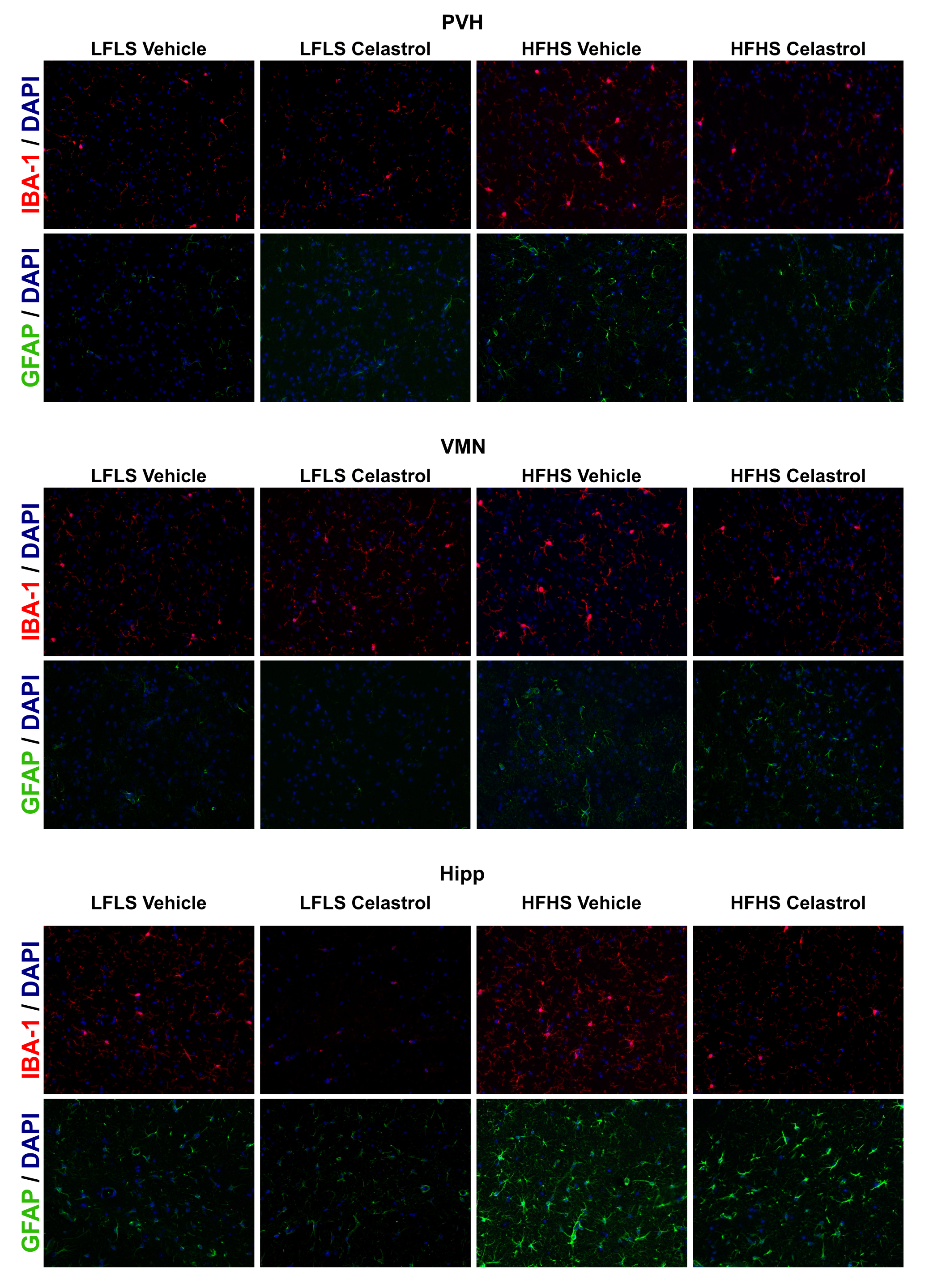


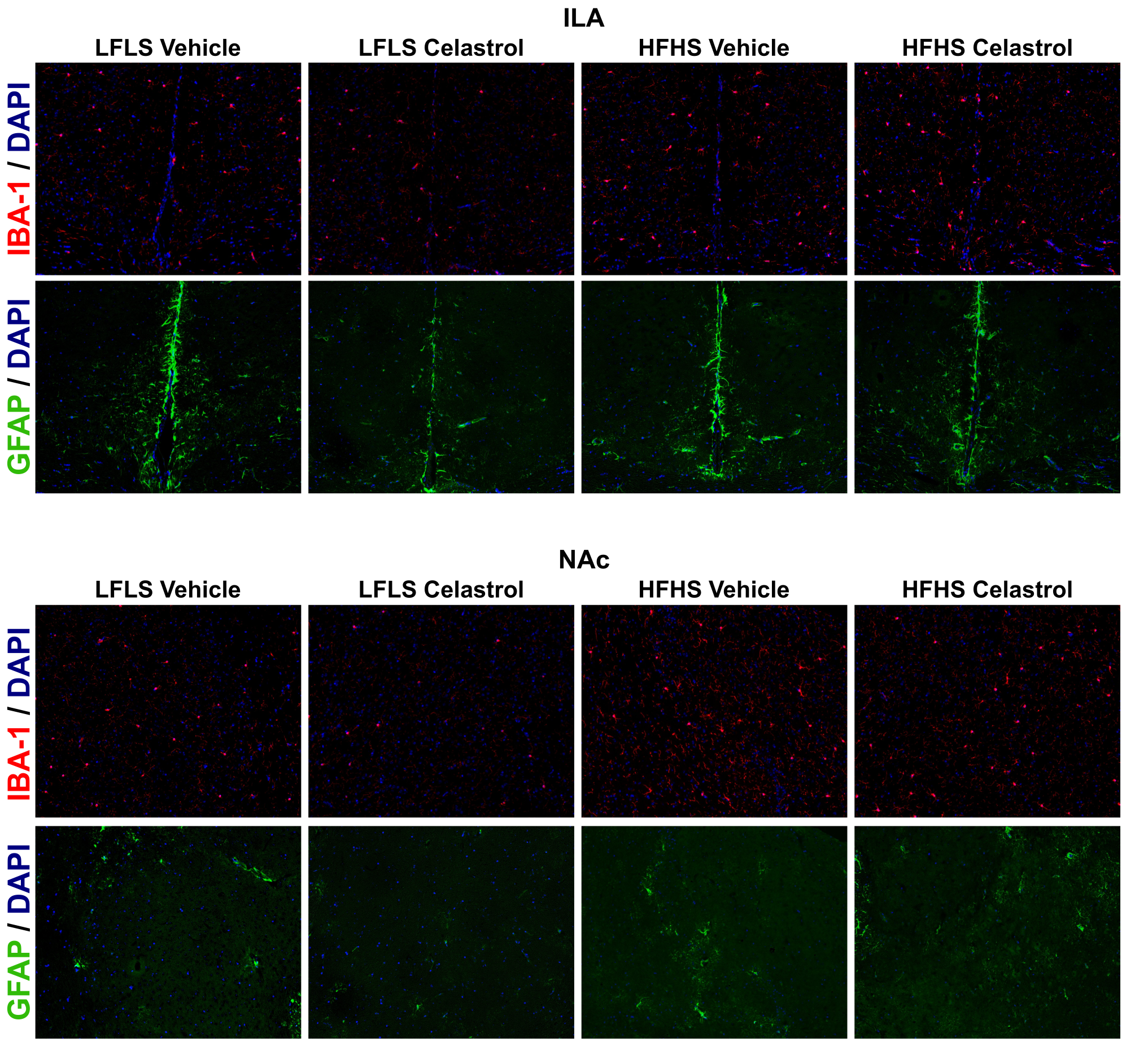

Supplement: Supplementary file 1 — Supplementary Material 1. [file 10020_2026_1530_MOESM1_ESM.docx]
