# Supplementary material for: Acute anti-obesity treatment with celastrol reduces body weight, cerebral inflammation and metabolic imbalances in mice
Source: Mol Med. 2026 Jul 1;32:111. doi: 10.1186/s10020-026-01530-4 (PMC13366973; doi:10.1186/s10020-026-01530-4)
Supplement: Supplementary file 2 — Supplementary Material 2. [file 10020_2026_1530_MOESM2_ESM.docx]

**Supplementary material PCA**

1. **PCA + clustering analysis**

*Introduction and methods*

Data from IF images was subjected to a PCA + clustering analysis to explore the potential existence of common morphological patterns of microglia and astrocytes on the different animal groups and associate them with specific phenotypes. PCA is an unsupervised method that aims to identify possible interrelationships between subsets of a specific data set, while preserving as much information as possible (Ringnér 2008). The objective of PCA is to find new variables, which are ordered by descending variance. The first component comprises a linear combination of all spatial variables weighed to capture the most relevant part of the original sample's variance, while the second component is orthogonal to the first and explains the maximum possible residual variance. This is repeated as many times as necessary until the total variance is completely explained. In this study, the number of retained principal components was chosen to explain ≥ 80% of the variance. Next, by clustering individual values n the PCA space, and using k-means, we aimed to find cell types with common characteristics, with a particular interest in potentially distinguishing the HFHS vehicle group (the “non-responder” group).

To build the PCA, we used the continuous variables measured in Image J: "Average.Size", "%Occupied.Area","Perimeter" ,"Circularity", "Solidity” and ","Count.by.area”, for astrocytes and microglia, separately. Specifically, each region analyzed (ARC, VMN, PVN, Hipp, NAc and ILA) was divided into different IF image quadrants (ARC 6-9 quadrants, VMN 6 quadrants, PVH 6 quadrants, Hipp 12, ILA 3 , NAc 12), and average values of the abovementioned variables calculated. Next, we used those values for the PCA+ clustering analysis. PCA was performed using the *prcomp* function of the stats R package (R Core Team 2025) in R, for each region independently. The corresponding eigenvalues and variance explained of the PCA was obtained, loadings of the variables on PCA were observed, as well as potential natural group separation in the PCA1/PCA2 space. Next, clustering was performed using the first two PCA using *kmeans* function of the stats package (R Core Team 2025), with 3 or 4 clusters, depending on the region, till the HFHS-vehicle cluster was identified, if possible. Proportion tables containing the % of points of the cluster from each diet or treatment are provided. reported. Finally, the PCA and corresponding variables driving such clusters were reported.

*Results*

**ARC microglia**

In the ARC, two components explained >85% of the variance (**Table S1**). PCA loadings and contributions showed high contribution of perimeter and %of occupied area, among others, and PCA1 separated quite good the treated vs non treated groups. (**Figure S2**).

|  | **eigenvalue** | **variance.percent** | **cumulative.variance.percent** |
| --- | --- | --- | --- |
| Dim.1 | 3.62366401 | 60.3944002 | 60.39440 |
| Dim.2 | 1.50857347 | 25.1428912 | 85.53729 |
| Dim.3 | 0.78335206 | 13.0558676 | 98.59316 |
| **Table S1.** PCA eigenvalues and variance explained on ARC microglia | | | |

| **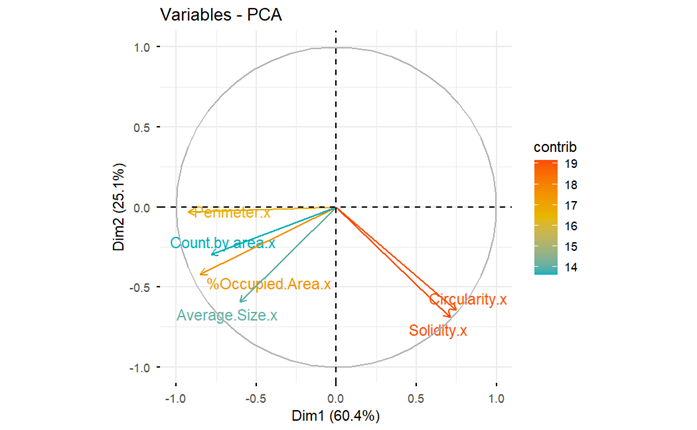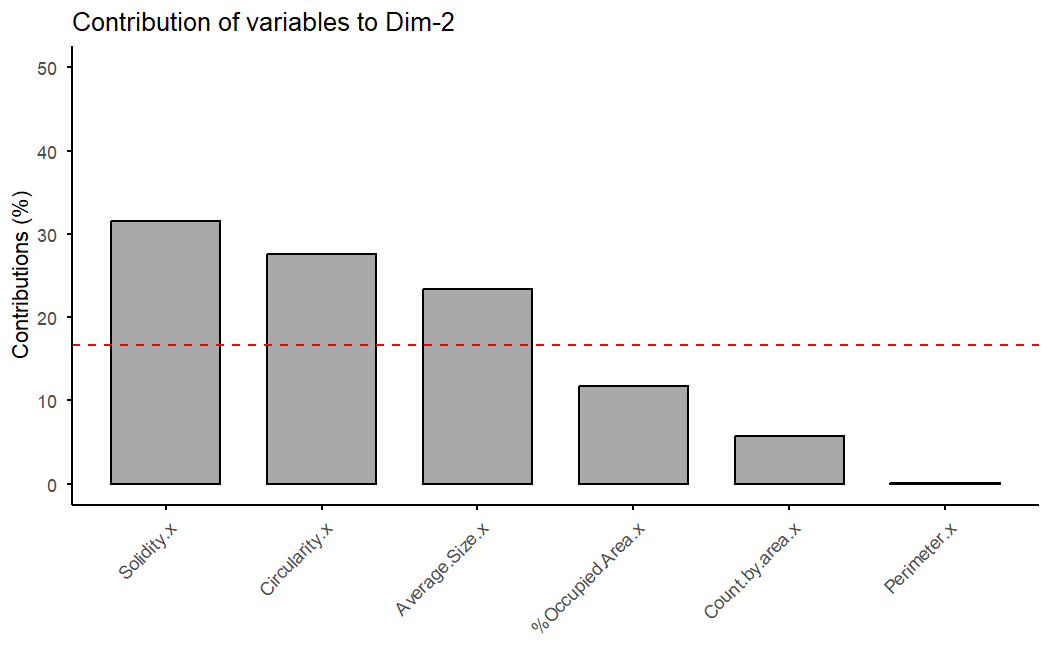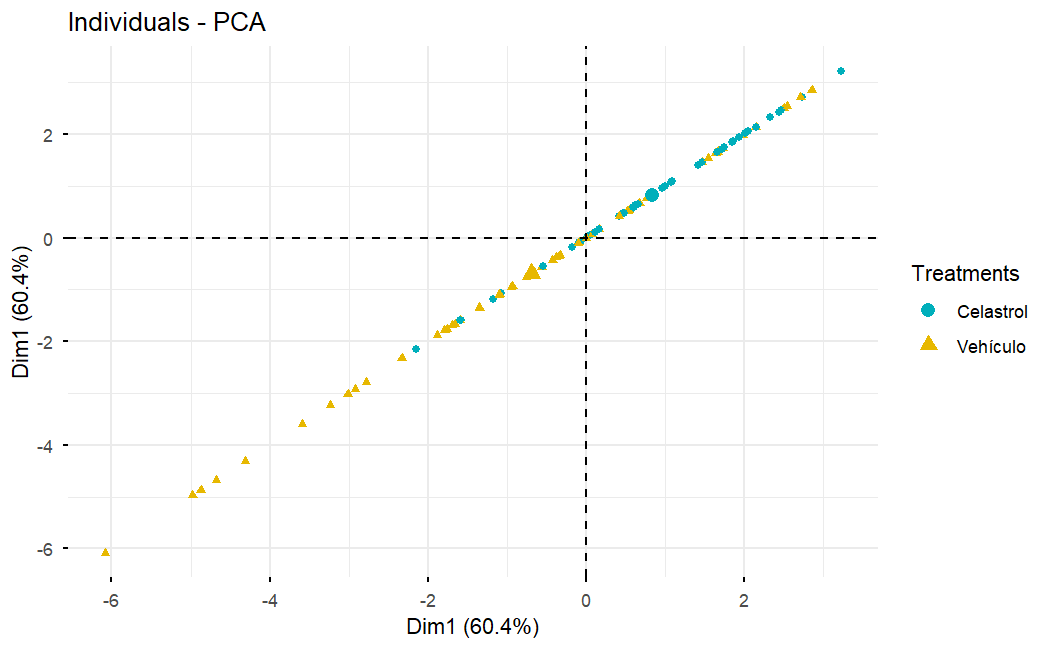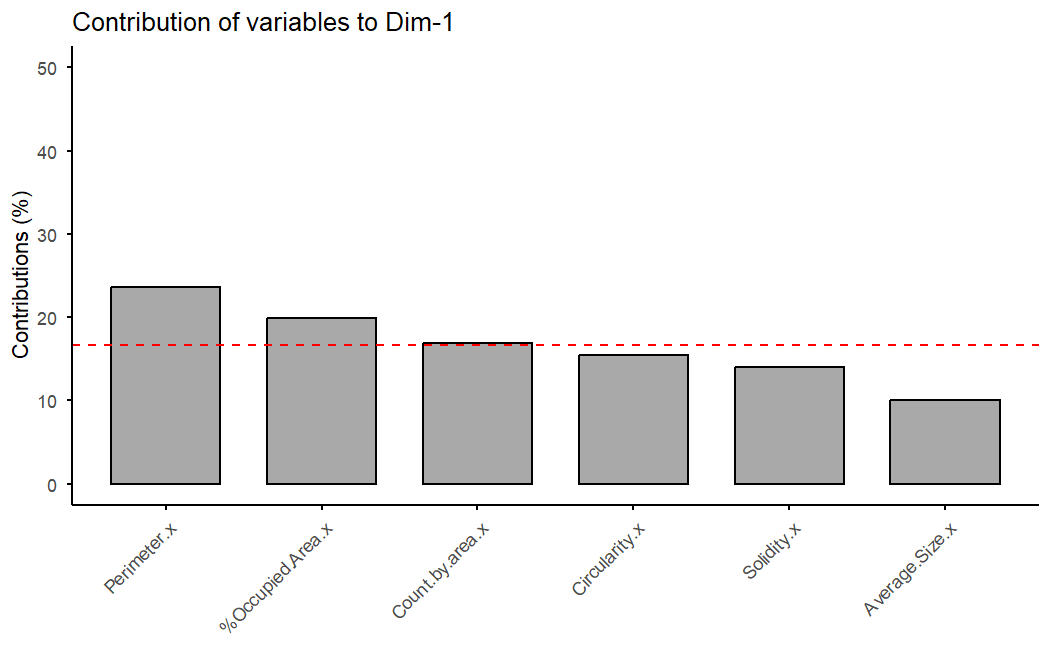** |
| --- |
| **Figure S1. PCA on ARC microglia** |

Mean values per group and tests on the variables composing the PCA were assessed with a lme, including the potential interaction between diet and treatment, and mouse as a random term, and wald tests performed (**Table S2**). Interaction was not significant in any of the variables assessed, and diet and treatment affected significantly some of the variables (Table S2).

| **Variable** | **means_CTRL_cel** | **means_HFHS_cel** | **means_CTRL_Veh** | **means_HFHS_Veh** | **p_diet** | **p_treatment** |
| --- | --- | --- | --- | --- | --- | --- |
| **Average.Size.x** | 452.28 | 433.90 | 477.17 | **557.495** | 0.282 | **0.050** |
| perc_Occupied_Area_x | 0.7745 | 0.880 | 0.950 | **1.8383** | 0.184 | 0.085 |
| **Perimeter.x** | 120.30 | 125.56 | 130.72 | **146.071** | 0.092 | **0.026** |
| Circularity.x | 0.43 | 0.38 | 0.402 | 0.3699 | **0.0100** | 0.143 |
| Solidity.x | 0.70 | 0.66 | 0.68 | 0.6547 | **0.0436** | 0.413 |
| Count.by.area.x | 16.93 | 20.728 | 19.72 | 31.811 | 0.171 | 0.123 |
| **Table S2.** Mean values and tests of microglial descriptors in the ARC | | | | | | |

Clustering in the microglial ARC yielded a cluster composed of 100% vehicle and 92% HFHS celastrol (**Figure S2**). This group is characterized by the lowest PCA1. This translates into highest perimeter and %of occupied area.

| 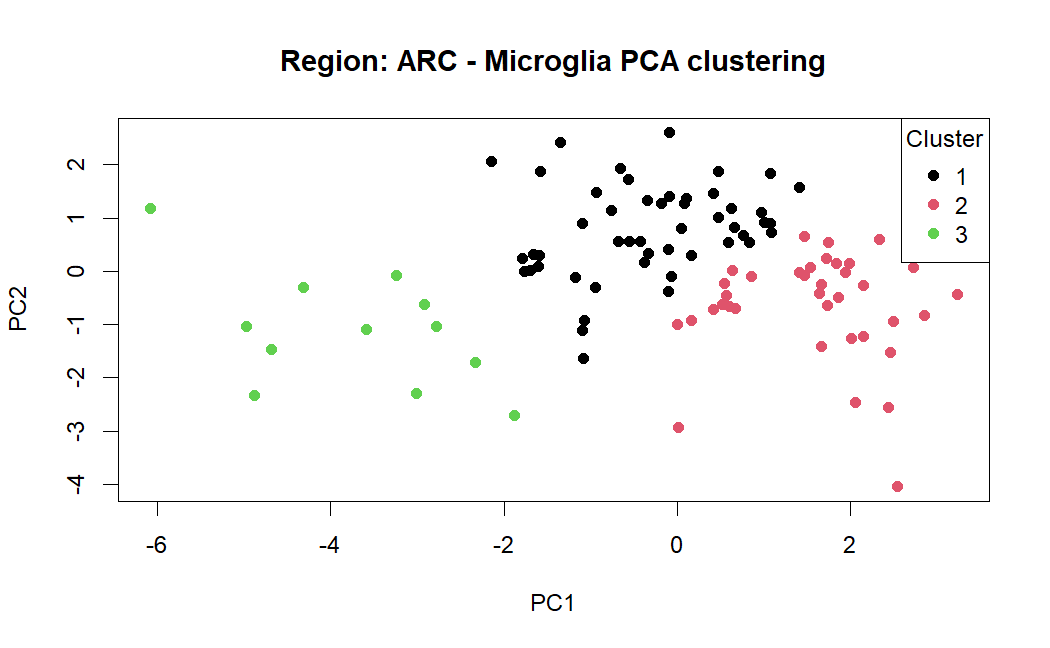 | clust Celastrol Vehicle  1 0.4897959 0.5102041  2 0.5526316 0.4473684  **3 0.0000000 1.0000000**  clust CTRL HFHS  1 0.44897959 0.55102041  2 0.73684211 0.26315789  **3 0.08333333 0.91666667** |
| --- | --- |
| **Figure S2.** PCA k-means clustering of microglial descriptors in the ARC and proportion tables of each experimental group (right) | |

**PVN microglia**

A similar PCA + clustering analysis is presented for the PVN, where the first two components explained 80% of the variance (**Table S3**).

|  | **eigenvalue** | **variance.percent** | **cumulative.variance.percent** |
| --- | --- | --- | --- |
| Dim.1 | 3.15752055 | 52.6253425 | 52.62534 |
| Dim.2 | 1.66715666 | 27.7859443 | 80.41129 |
| Dim.3 | 1.09374831 | 18.2291384 | 98.64043 |
| **Table S3. PCA eigenvalues and variance explained on PVN microglia** | | | |

PCA loadings and variable contribution are presented in **figure S3**. Separation in the PCA1/PCA2 space is not that clear.

| **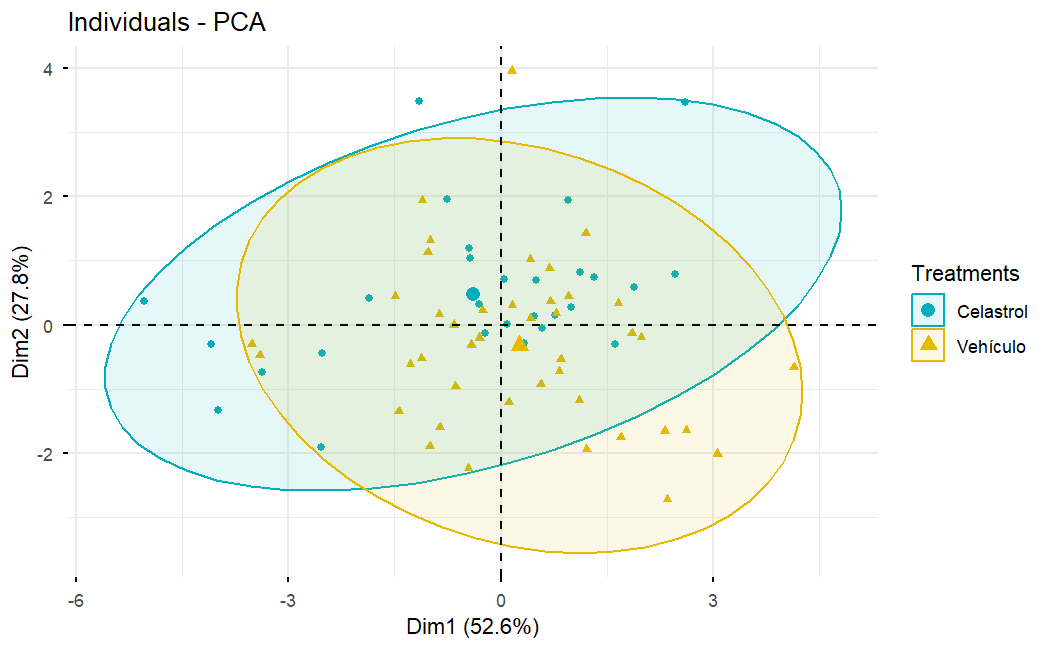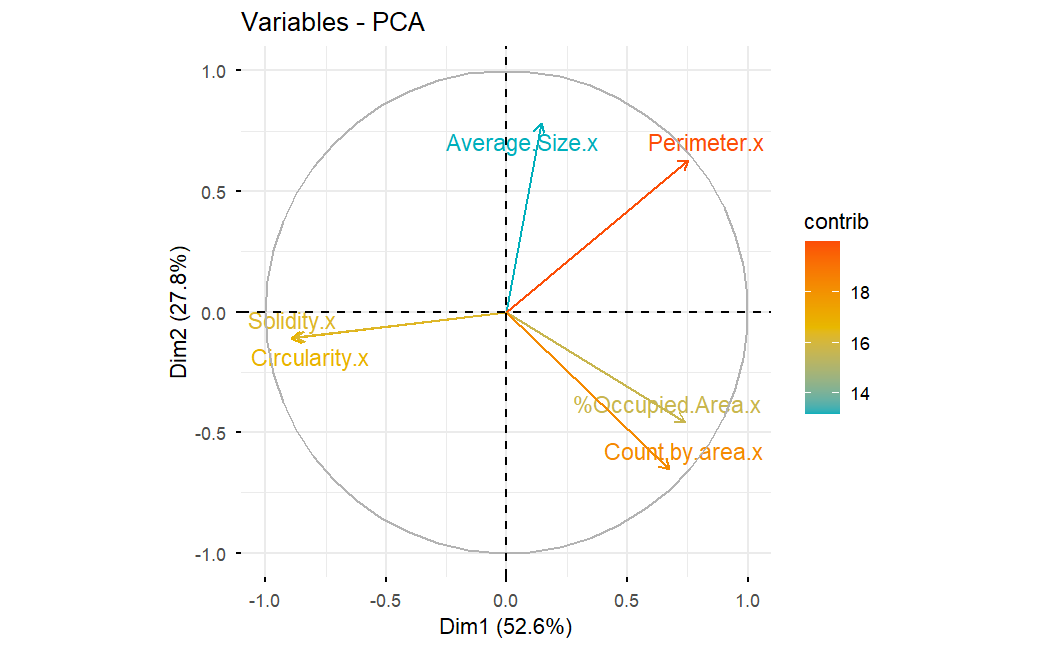**  **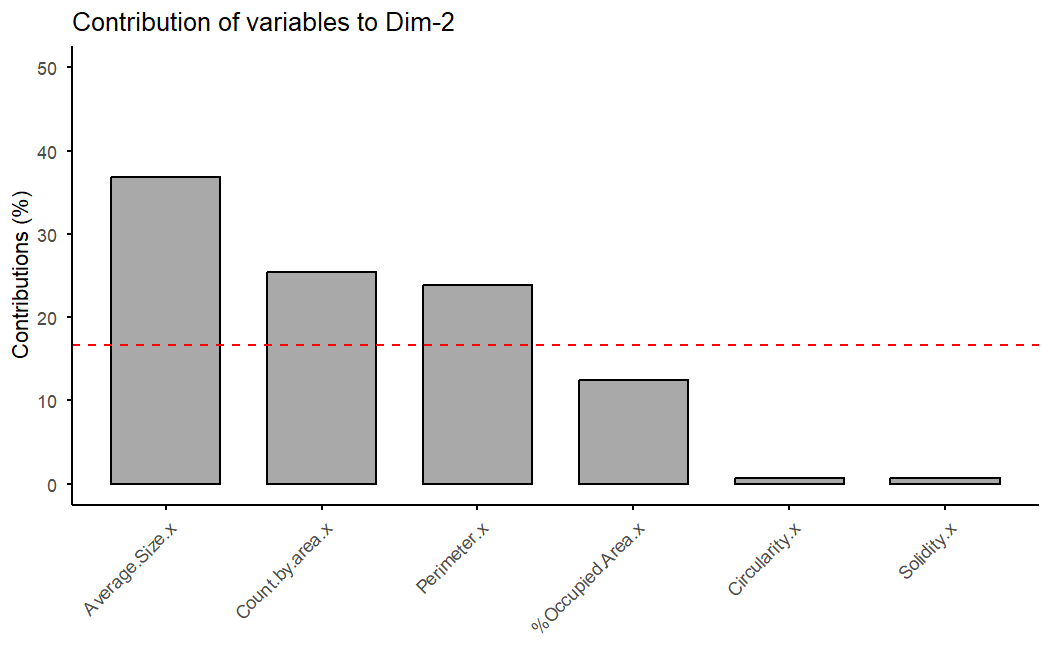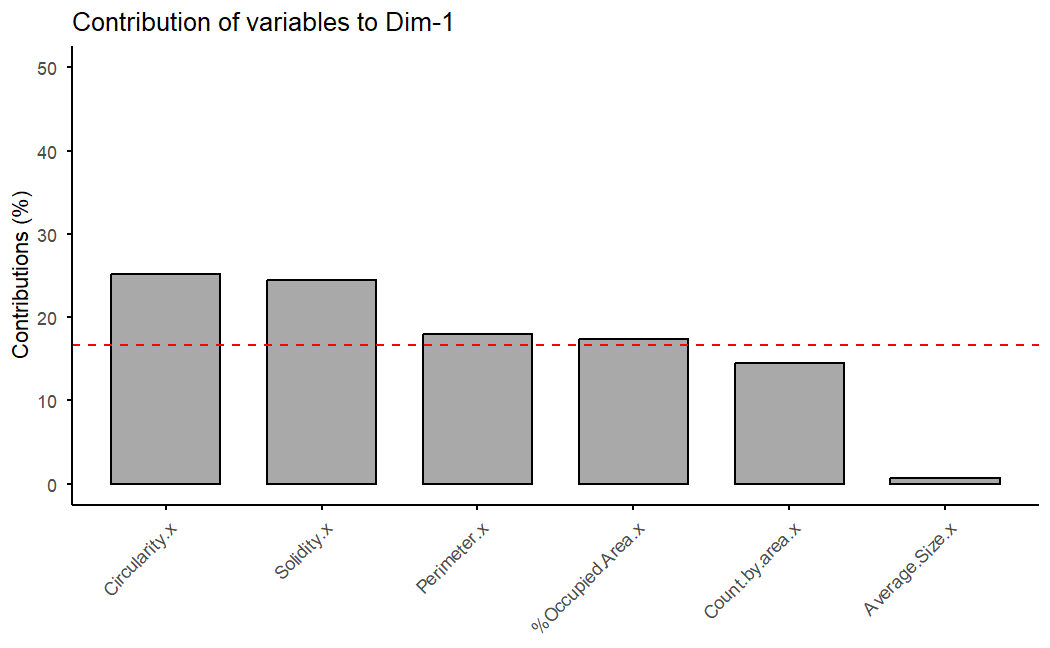** |
| --- |
| **Figure S3. Microglia PCA loadings on PVN** |

And corresponding mean group values and tests on variables (**Table S4**)

| **Variables** | **CTRL_cel** | **HFHS_cel** | **CTRL_veh** | **HFHS_veh** | **P_diet** | **P_treat** |
| --- | --- | --- | --- | --- | --- | --- |
| Average.Size.x | 498.42 | 477.50 | 438.08 | 502.78 | 0.439 | 0.815 |
| **perc_Occupied_Area_x** | **0.76** | **0.58** | **0.81** | **1.14** | 0.524 | **0.047** |
| Perimeter.x | 148.11 | 139.24 | 134.36 | 145.76 | 0.764 | 0.959 |
| Circularity.x | 0.35 | 0.35 | 0.35 | 0.33 | 0.638 | 0.659 |
| Solidity.x | 0.62 | 0.63 | 0.62 | 0.61 | 0.727 | 0.720 |
| **Count.by.area.x** | **14.91** | **12.81** | **18.58** | **23.61** | 0.557 | **0.043** |
| **Table S4.** Mean values and tests of microglial descriptors in the PVN | | | | | | |

| 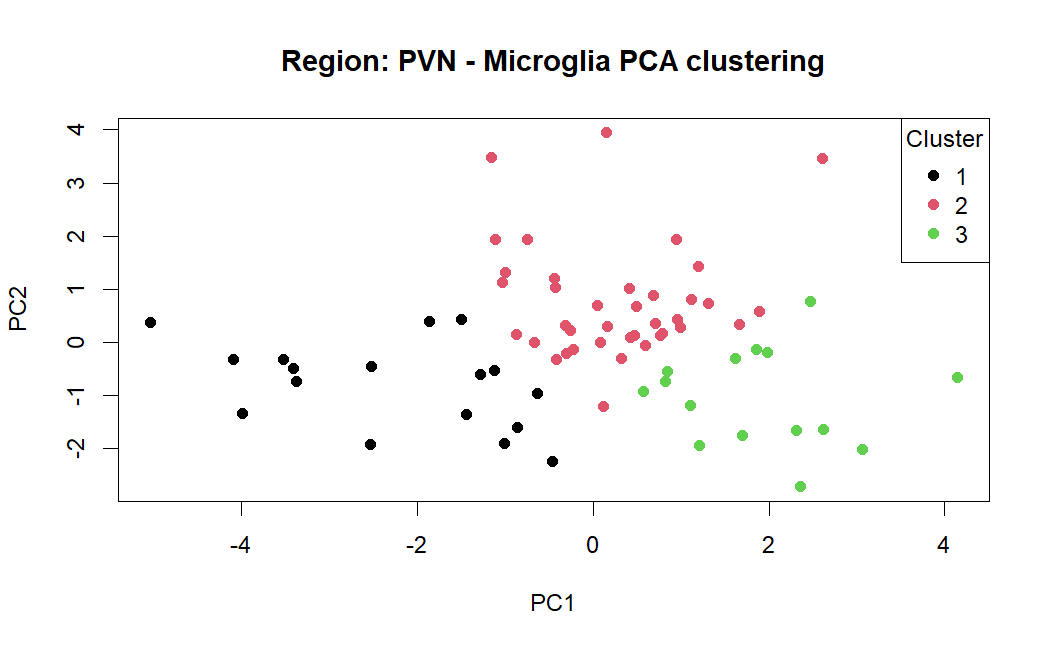 | Proportion tables  clust Celastrol Vehicle  1 0.4117647 0.5882353  2 0.5000000 0.5000000  **3 0.1333333 0.8666667**    clust CTRL HFHS  1 0.6470588 0.3529412  2 0.4210526 0.5789474  **3 0.3333333 0.6666667** |
| --- | --- |
| **Figure S5.** Clustering on PVN microglia and proportion tables of each experimental group (right) | |

Cluster 3, 87% vehicle and 67% HFHS, characterized by high PCA1 with negative PCA2, which translates in variables as those with small circularity and solidity but high perimeter and %occupied area.

**VMN microglia**

A similar analysis of PCA eigenvalues, component loads, contribution to explained varaince and group separation in the PCA space for the VMN microglia is presented from figures S6-7 and tables S6-**7.**

|  | **eigenvalue** | **variance.percent** | **cumulative.variance.percent** |
| --- | --- | --- | --- |
| Dim.1 | 3.23975052 | 53.9958420 | 53.99584 |
| Dim.2 | 1.49283591 | 24.8805985 | 78.87644 |
| Dim.3 | 1.19675344 | 19.9458907 | 98.82233 |
| **Table S6.** PCA eigenvalues and variance explained on microglia VMN | | | |
| \| **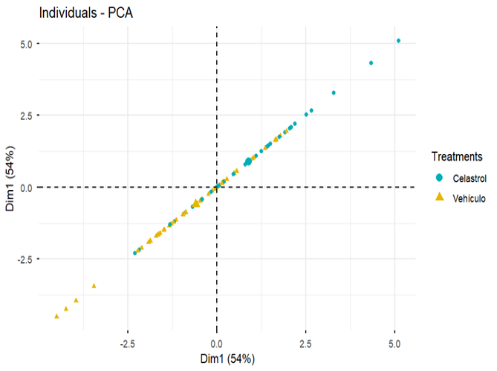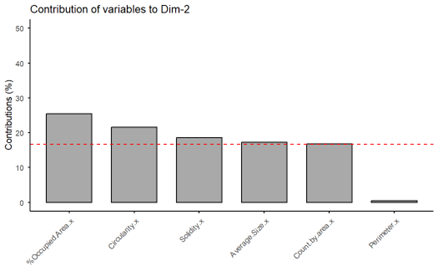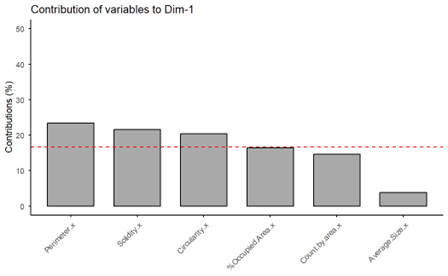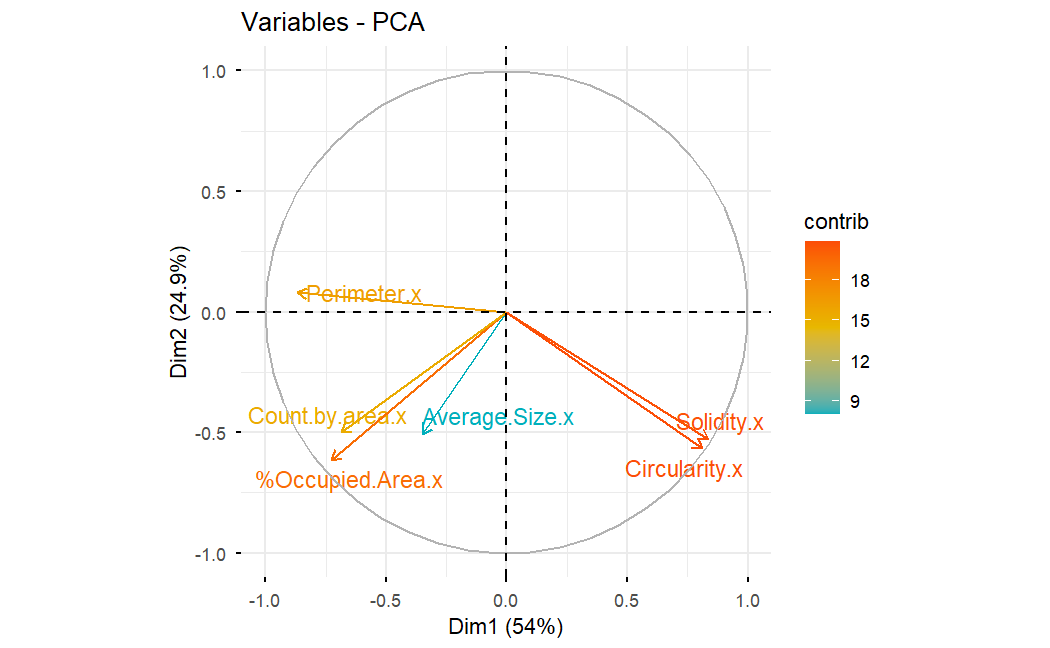** \| \| --- \| \| **Figure S6. PCA on microlgia VMN** \| | | | |

| **Variables** | **CTRL_cel** | **HFHS_cel** | **CTRL_veh** | **HFHS_veh** | **P_diet** | **P_treat** |
| --- | --- | --- | --- | --- | --- | --- |
| Average.Size.x | 446.07 | 408.79 | 464.46 | **502.33** | 0.788 | 0.085 |
| Circularity.x | 0.39 | 0.39 | 0.35 | 0.34 | 0.774 | 0.070 |
| Count.by.area.x | 14.67 | 11.86 | 16.77 | 23.67 | 0.507 | 0.090 |
| perc_Occupied_Area_x | 0.65 | 0.48 | 0.76 | **1.20** | 0.409 | 0.062 |
| **Perimeter.x** | 129.03 | 122.25 | 137.82 | **145.62** | 0.633 | **0.013** |
| Solidity.x | 0.65 | 0.67 | 0.62 | 0.61 | 0.896 | 0.054 |
| Table S7. Mean values and tests on the variables from microglia VMN | | | | | | |

| 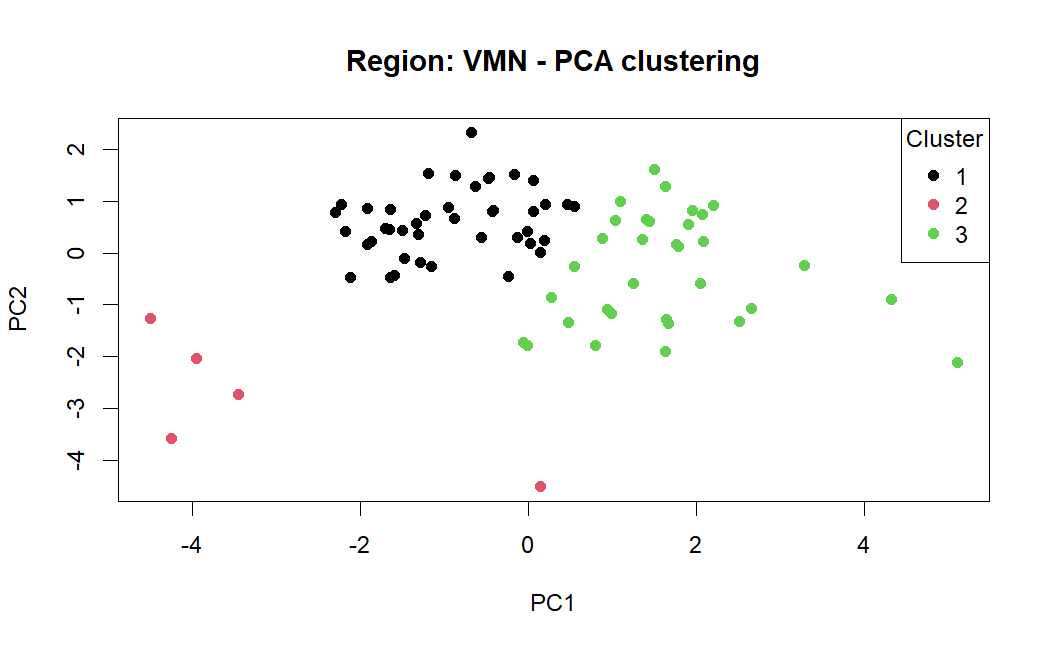 | **Proportion tables**    clust Celastrol Vehicle  1 0.3095238 0.6904762  **2 0.0000000 1.0000000**  3 0.5757576 0.4242424    clust CTRL HFHS  1 0.6190476 0.3809524  **2 0.0000000 1.0000000**  3 0.6060606 0.3939394 |
| --- | --- |
| **Figure S7**. PCA k-means clustering in the VMN micgrolia and proportion tables of each experimental group (right) | |

HFHS vehicle cellular cluster in the VMN is characterized by the low (negative) PCA1 and low negative PCA2, which translates into high perimeter, counts and %occupied area but low solidity

**HIPPOCAMPUS microglia**

An analogous analysis of PCA eigenvalues, component loads, contribution to explained variance and group separation in the PCA space for the hippocampal microglia is presented from figures S8-9 and tables S8-10.

|  | **eigenvalue** | **variance.percent** | **cumulative.variance.percent** | |
| --- | --- | --- | --- | --- |
| Dim.1 | 3.75880186 | 62.6466976 | 62.64670 | |
| Dim.2 | 1.16868755 | 19.4781258 | 82.12482 | |
| Dim.3 | 1.00194329 | 16.6990548 | 98.82388 | |
| **Table S8**. PCA eigenvalues and variance explained in the hippocampal microglia | | | | |
| 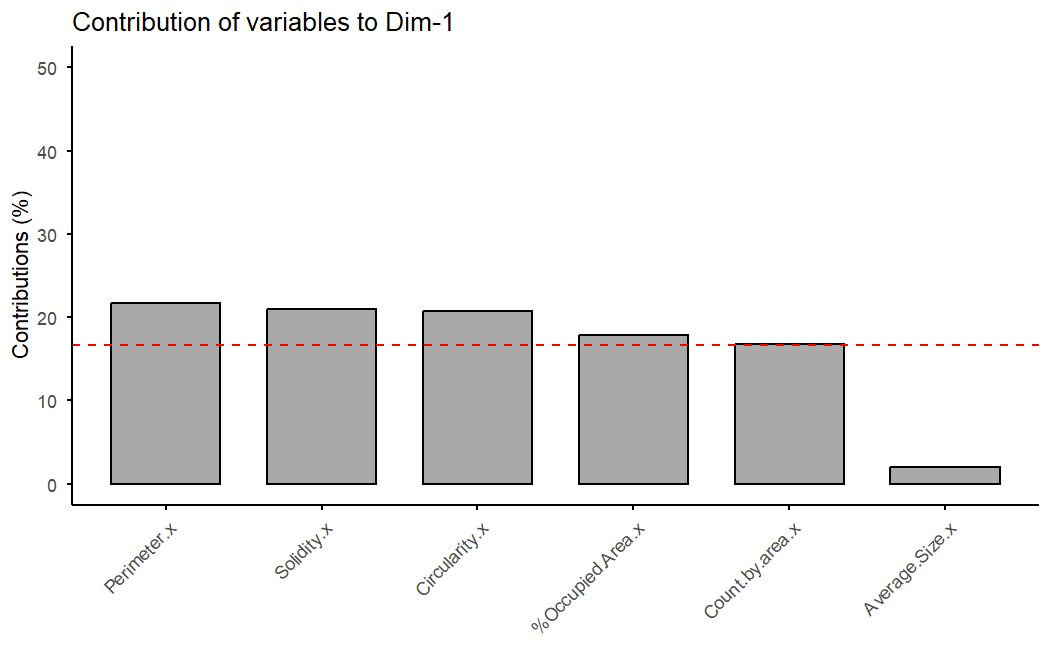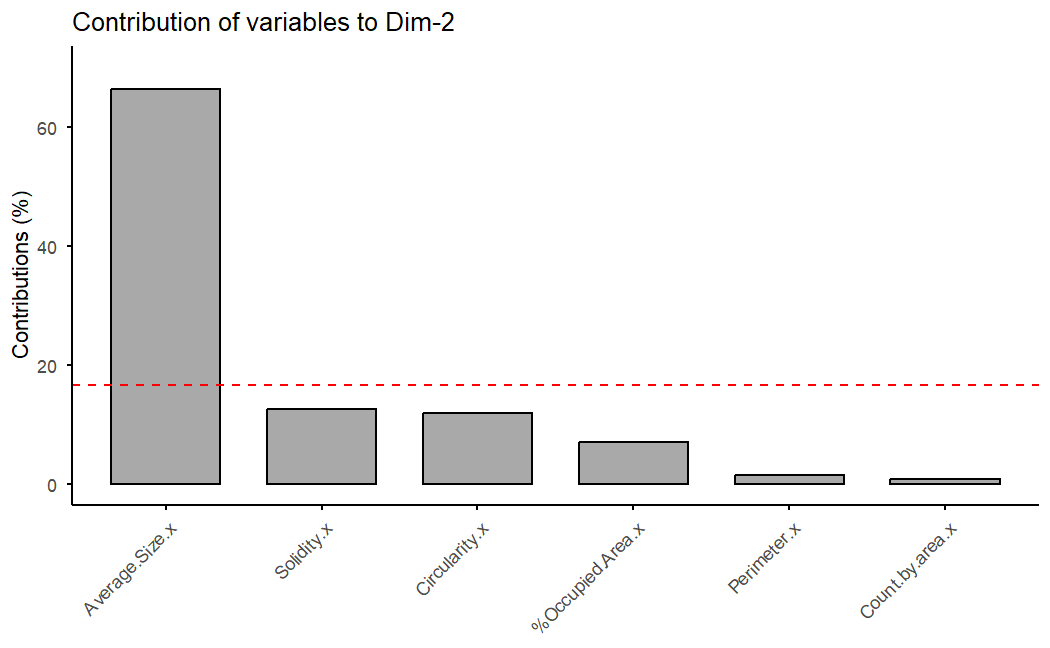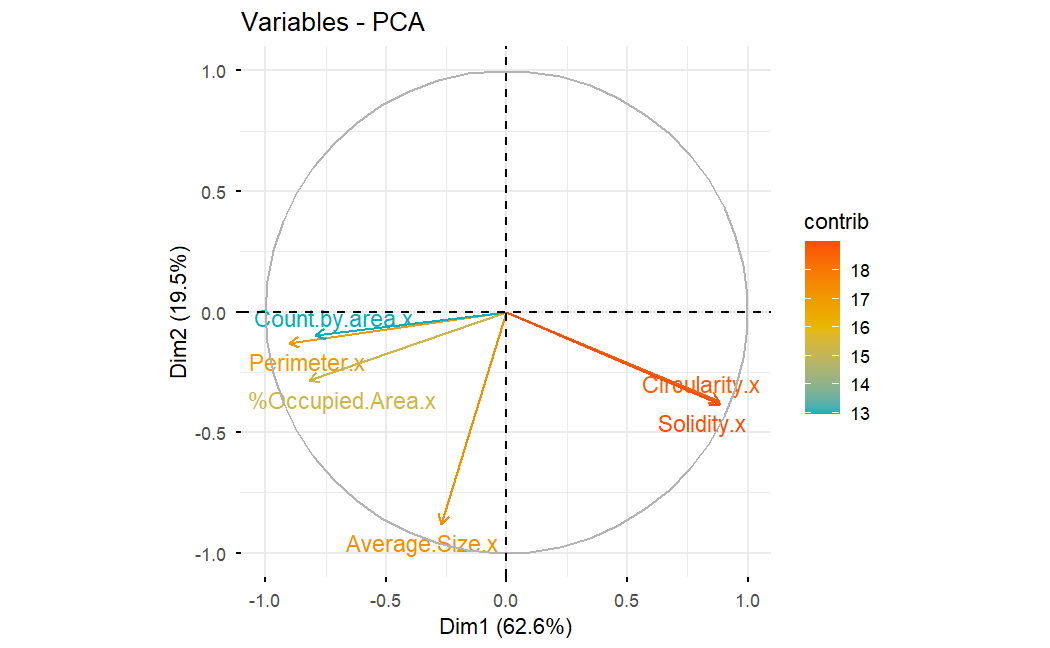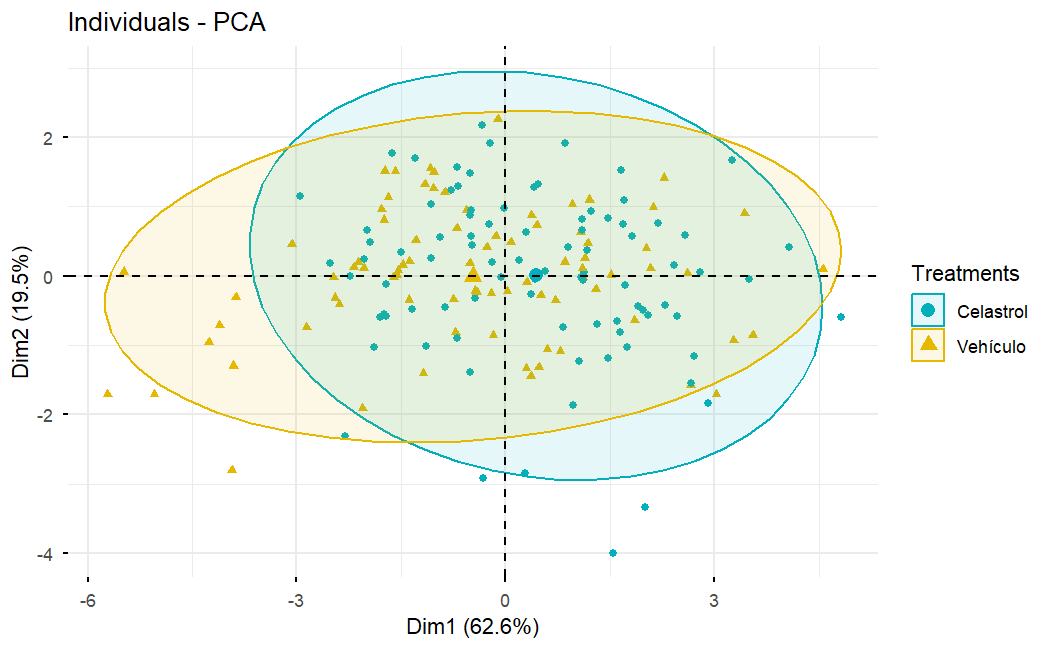 | | | |  |
| **Figure S8.** PCA loadings and variable contribution on hippocampal micgrolia descriptors | | | |  |

| **Variables** | **CTRL_cel** | **HFHS_cel** | **CTRL_veh** | **HFHS_veh** | **P_diet** | **P_treat** | P_interact |
| --- | --- | --- | --- | --- | --- | --- | --- |
| Average.Size.x | 480.96 | 488.30 | 480.51 | **491.81** | 0.716 | 0.990 | 0.91 |
| Circularity.x | 0.36 | 0.38 | 0.38 | 0.31 | 0.418 | 0.274 | **0.01** |
| Count.by.area.x | 17.66 | 18.53 | 16.61 | 33.18 | 0.868 | 0.731 | **0.00** |
| perc_Occupied_Area_x | 0.86 | 0.90 | 0.80 | 1.63 | 0.869 | 0.731 | **0.01** |
| Perimeter.x | 141.32 | 137.85 | 138.46 | **155.67** | 0.492 | 0.516 | **0.02** |
| Solidity.x | 0.63 | 0.65 | 0.65 | **0.58** | 0.315 | 0.267 | **0.0** |
| **Table S9.** Hippocampus average values and tests of microglial descriptors  Interaction was significant in many variables, and post-hoc tests were performed (**Table s10**) | | | | | | | |

| Diet | Contrast | Estimate | SE | Df | t.ratio | p.value | Variable | p.adj |
| --- | --- | --- | --- | --- | --- | --- | --- | --- |
| CTRL | Celastrol - Vehicle | -0.03 | 0.03 | 12.48 | -1.09 | 0.296 | Circularity.x | 0.296 |
| HFHS | Celastrol - Vehicle | 0.07 | 0.03 | 11.29 | 2.63 | 0.023 | Circularity.x | 0.046 |
| CTRL | Celastrol - Vehicle | 1.40 | 4.07 | 12.49 | 0.34 | 0.737 | Count.by.area.x | 0.737 |
| HFHS | Celastrol - Vehicle | -14.78 | 3.93 | 11.25 | -3.76 | 0.003 | Count.by.area.x | 0.006 |
| CTRL | Celastrol - Vehicle | 0.08 | 0.23 | 12.43 | 0.34 | 0.737 | perc_Occupied_Area_x | 0.737 |
| HFHS | Celastrol - Vehicle | -0.74 | 0.22 | 11.40 | -3.35 | 0.006 | perc_Occupied_Area_x | 0.012 |
| CTRL | Celastrol - Vehicle | 4.63 | 7.14 | 12.43 | 0.65 | 0.528 | Perimeter.x | 0.528 |
| HFHS | Celastrol - Vehicle | -17.80 | 6.96 | 11.42 | -2.56 | 0.026 | Perimeter.x | 0.052 |
| CTRL | Celastrol - Vehicle | -0.03 | 0.03 | 12.46 | -1.11 | 0.288 | Solidity.x | 0.288 |
| HFHS | Celastrol - Vehicle | 0.06 | 0.02 | 11.34 | 2.60 | 0.024 | Solidity.x | 0.04 |
| Table S10. Hippocampus post-hoc tests of microglial descriptors | | | | | | | | |

| 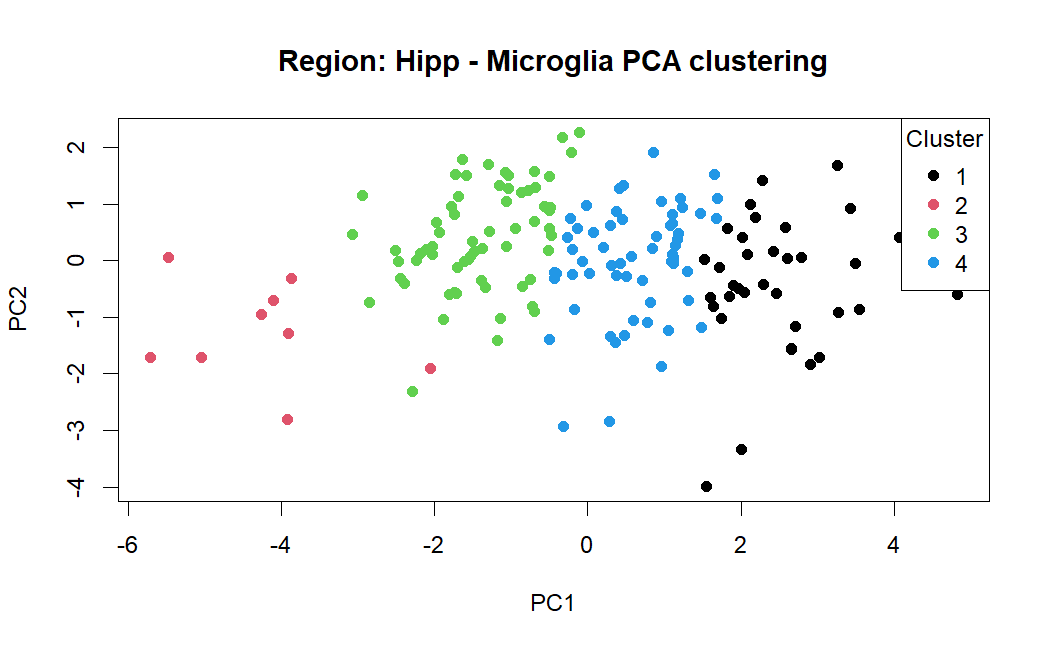 | clust Celastrol Vehicle  1 0.6388889 0.3611111  **2 0.0000000 1.0000000**  3 0.5000000 0.5000000  4 0.5500000 0.4500000    clust CTRL HFHS  1 0.6111111 0.3888889  **2 0.0000000 1.0000000**  3 0.4375000 0.5625000  4 0.4833333 0.5166667 |
| --- | --- |
| **Figure S9**. Clustering of hippocampal microglial descriptors (left) and proportion tables of experimental groups | |

**HFHS vehicle cluster** characterized by very negative PC1 and negative PCA2, which translates into high perimeter with low solidity and low circularity with high average size

**ILA microglia**

|  | **eigenvalue** | **variance.percent** | **cumulative.variance.percent** |
| --- | --- | --- | --- |
| Dim.1 | 4.256669710 | 70.94449516 | 70.94450 |
| Dim.2 | 1.038410830 | 17.30684717 | 88.25134 |
| Dim.3 | 0.628083545 | 10.46805909 | 98.71940 |
| **Table S11**. PCA eigenvalues and variance explained on microglial ILA | | | |

| 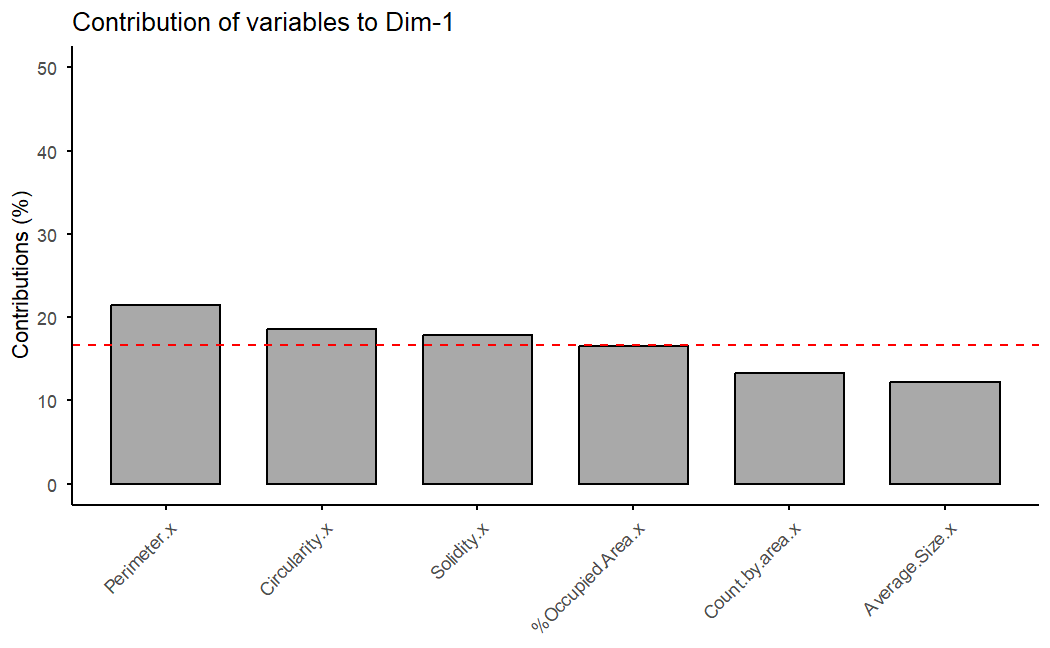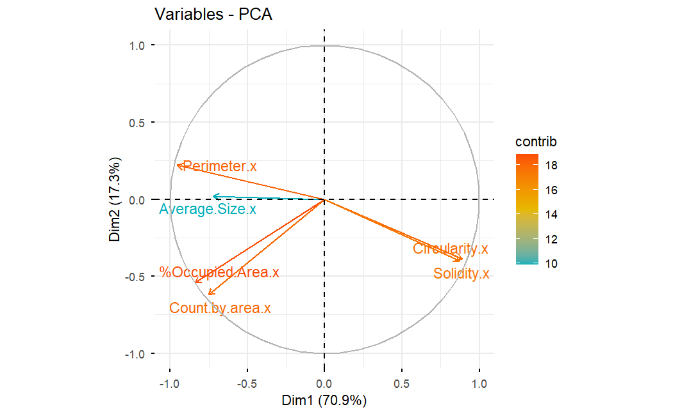 |
| --- |
| 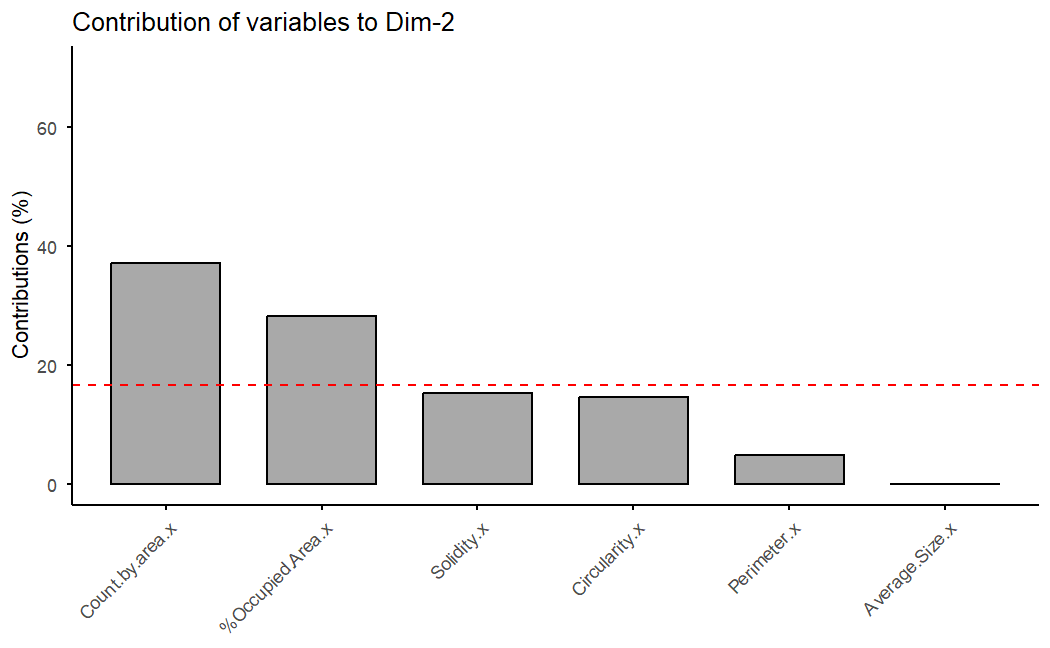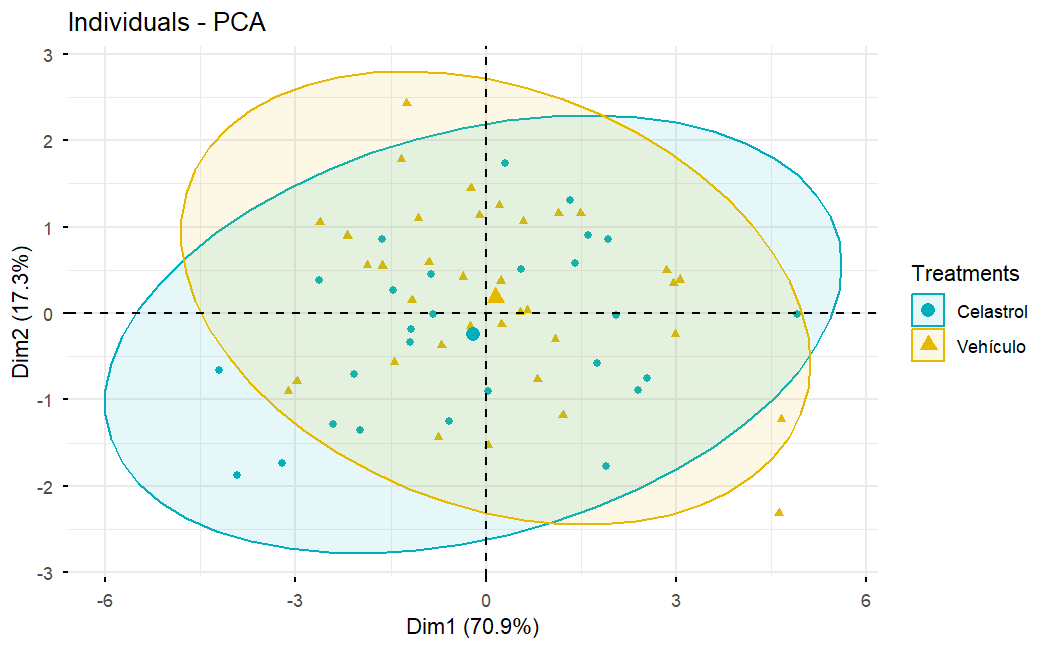 |
| **Figure S10**. PCA loadings and variable contributions on microglial ILA |

| **variable** | **means_CTRL_cel** | **means_HFHS_cel** | **means_CTRL_Veh** | **means_HFHS_Veh** | **p_diet** | **p_treatment** |
| --- | --- | --- | --- | --- | --- | --- |

| Average.Size.x | 301.49 | 326.93 | 306.51 | 326.62 | 0.118 | 0.814 |
| --- | --- | --- | --- | --- | --- | --- |
| Circularity.x | 0.34 | 0.31 | 0.36 | 0.29 | 0.002 | 0.789 |
| Count.by.area.x | 16.39 | **24.61** | 15.15 | **20.92** | 0.000 | 0.203 |
| perc_Occupied_Area_x | 0.51 | 0.83 | 0.46 | 0.69 | 0.000 | 0.230 |
| Perimeter.x | 116.05 | **128.24** | 110.99 | **131.15** | 0.003 | 0.885 |
| Solidity.x | 0.62 | **0.59** | 0.64 | **0.57** | 0.002 | 0.993 |
| **Table S12.** Mean values and tests of microglial ILA descriptors | | | | | | |

Only strong diet effects (no treatment effect)

| 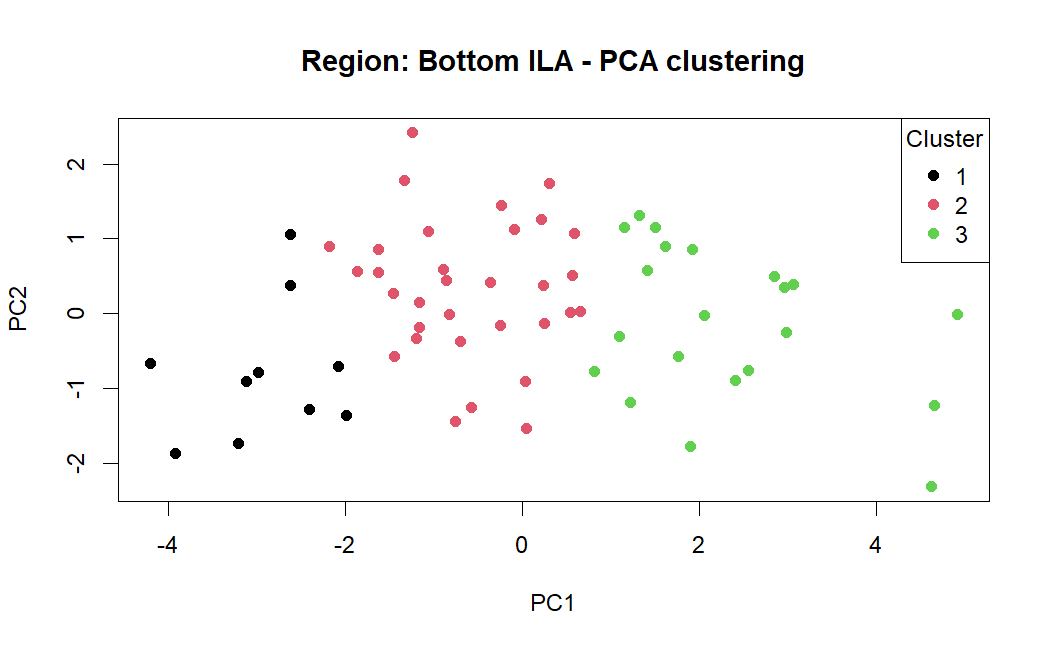 | | clust Celastrol Vehicle  1 0.7000000 0.3000000  2 0.3125000 0.6875000  3 0.4761905 0.5238095    clust CTRL HFHS  **1 0.1000000 0.9000000**  2 0.4375000 0.5625000  3 0.7142857 0.2857143 |
| --- | --- | --- |
| **Figure S11**. PCA k-means clustering on ILA microglial descriptors and proportion tables of each cluster in terms of experimental conditions (right) | |  |

Cluster 1, 90% HFHS, with no strict separation cleastrol/vehicle. HFHS characterized by very negative PCA and negative PCA2, which translates into high perimeter but low solidity and circularity and high counts by area

**NAC microglia**

|  | **eigenvalue** | **variance.percent** | **cumulative.variance.percent** |
| --- | --- | --- | --- |
| Dim.1 | 4.65885344 | 77.6475574 | 77.64756 |
| Dim.2 | 0.94765360 | 15.7942266 | 93.44178 |
| **Table S12**. PCA eigenvalues and variance explained on NAc microglia | | | |

| 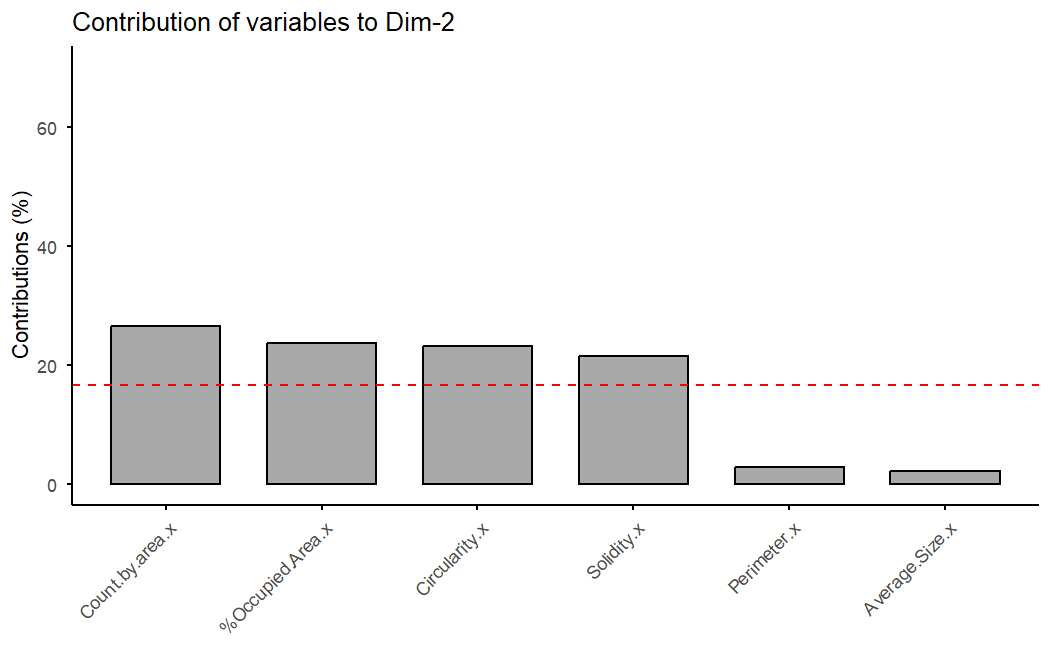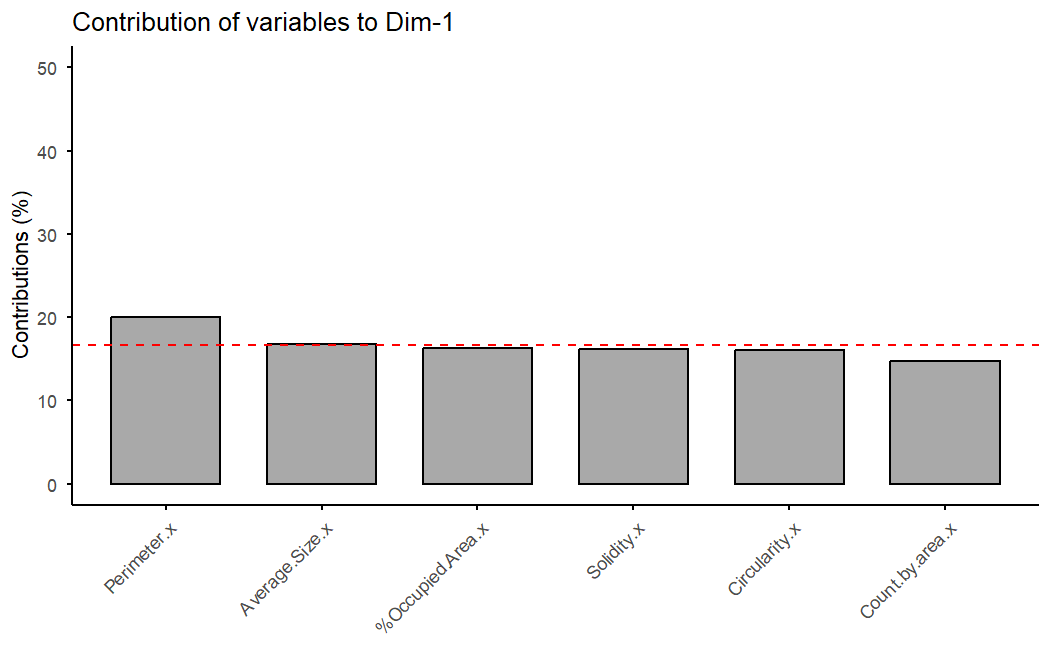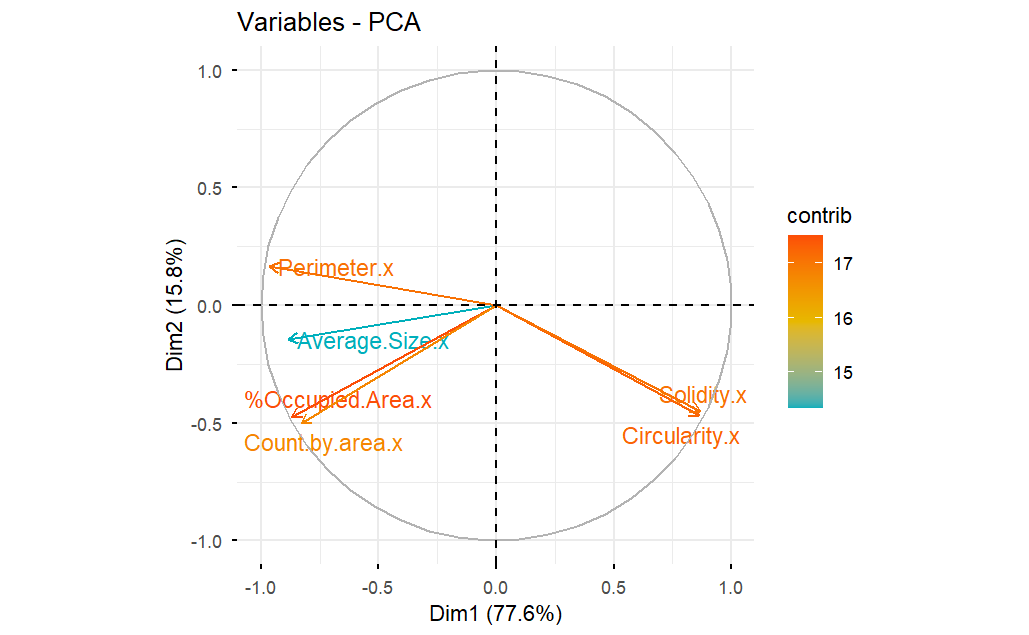 |
| --- |
| **Figure S12**. PCA loadings and variable contribution for NAc microglia |


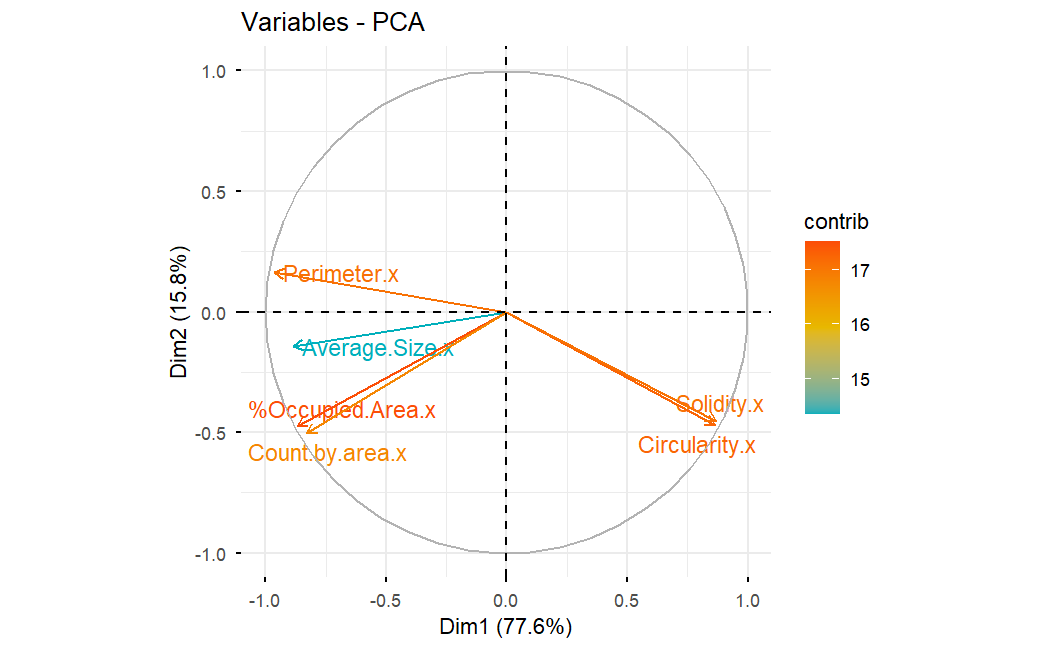


| **Variables** | **CTRL_cel** | **HFHS_cel** | **CTRL_veh** | **HFHS_veh** | **P_diet** | **P_treat** |
| --- | --- | --- | --- | --- | --- | --- |
| Average.Size.x | 271.21 | **310.48** | 277.81 | **330.40** | 0.000 | 0.446 |
| Circularity.x | 0.33 | 0.28 | 0.31 | 0.26 | 0.001 | 0.297 |
| Count.by.area.x | 12.24 | 17.58 | 10.54 | 22.41 | 0.000 | 0.722 |
| perc_Occupied_Area_x | 0.34 | 0.57 | 0.30 | 0.76 | 0.000 | 0.582 |
| Perimeter.x | 111.05 | **132.77** | 116.15 | **141.12** | 0.000 | 0.384 |
| Solidity.x | 0.60 | 0.55 | 0.59 | 0.54 | 0.002 | 0.370 |
| **Table S13**. Mean values and tests of microglial descriptors in the NAc | | | | | | |


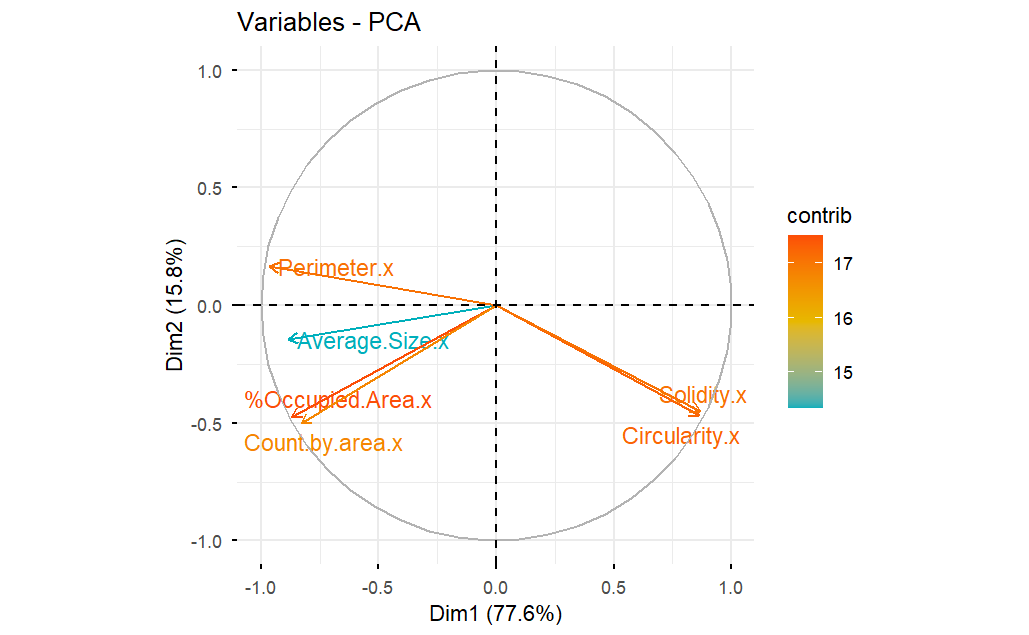


| 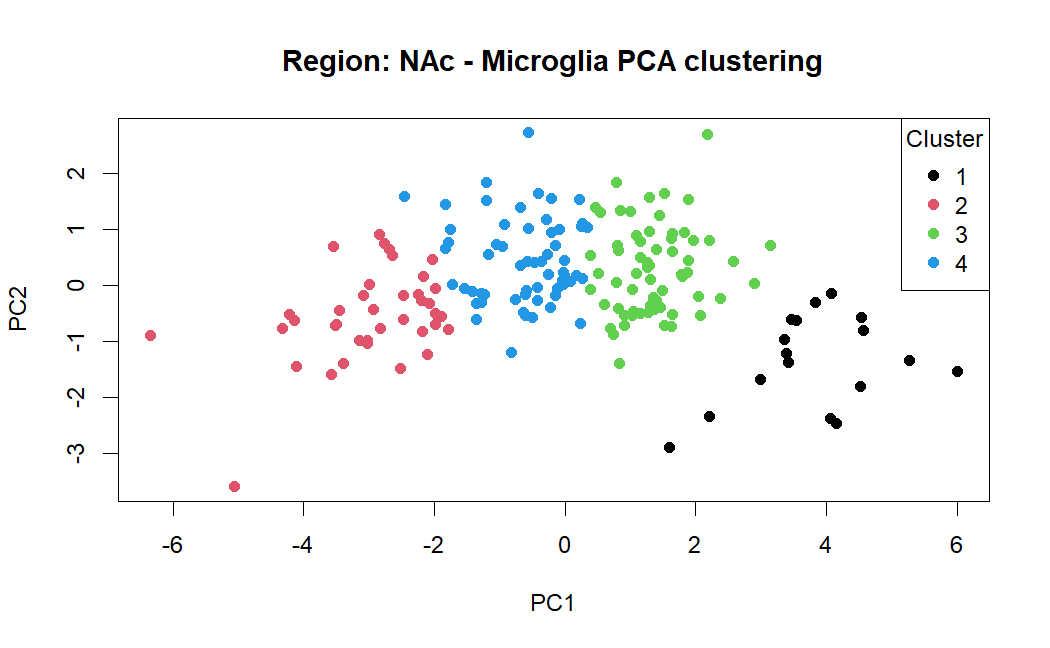 | 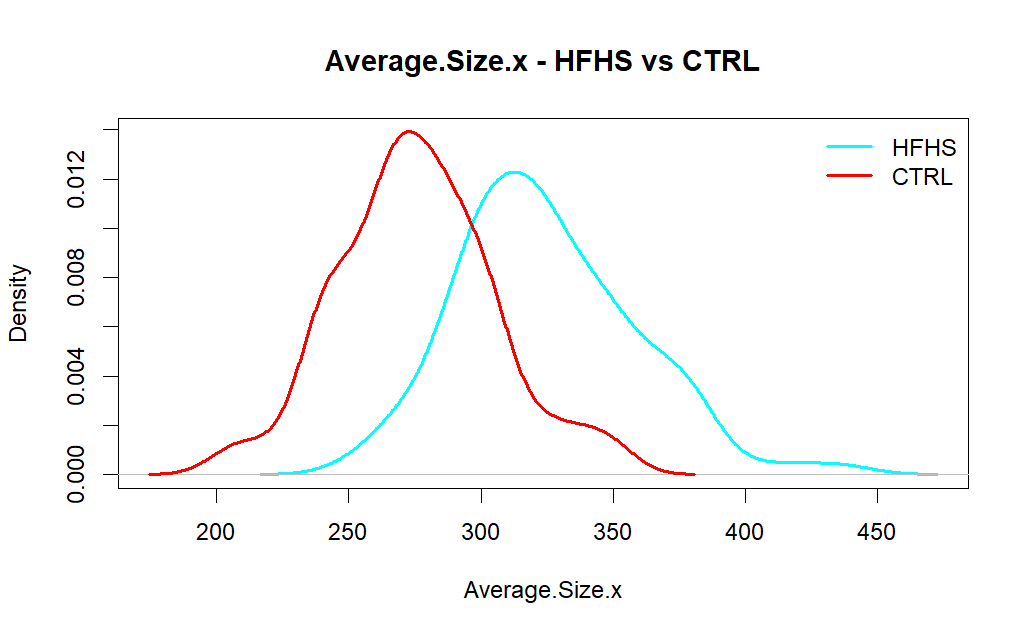clust Celastrol Vehicle  1 0.6470588 0.3529412  2 0.3684211 0.6315789  3 0.5396825 0.4603175  4 0.5166667 0.4833333    clust CTRL HFHS  1 0.88235294 0.11764706  **2 0.07894737 0.92105263**  3 0.73015873 0.26984127  4 0.40000000 0.60000000 |
| --- | --- |
| **Figure S13**. PCA k-means clustering of microglial descriptors in the NAc and corresponding proportion tables in terms of experimental conditions | |

**Cluster 2 92% HFHS,** characterized by the lowest values of PCA1, which translates into high perimeter and high average size with low solidity and circularity

**ARC ASTROCYTES**

|  | **eigenvalue** | **variance.percent** | **cumulative.variance.percent** |
| --- | --- | --- | --- |
| Dim.1 | 3.62483615 | 60.4139359 | 60.41394 |
| Dim.2 | 1.43307408 | 23.8845679 | 84.29850 |
| **Table S14**. PCA eigenvalues and variance explained on ARC astrocytes | | | |

| **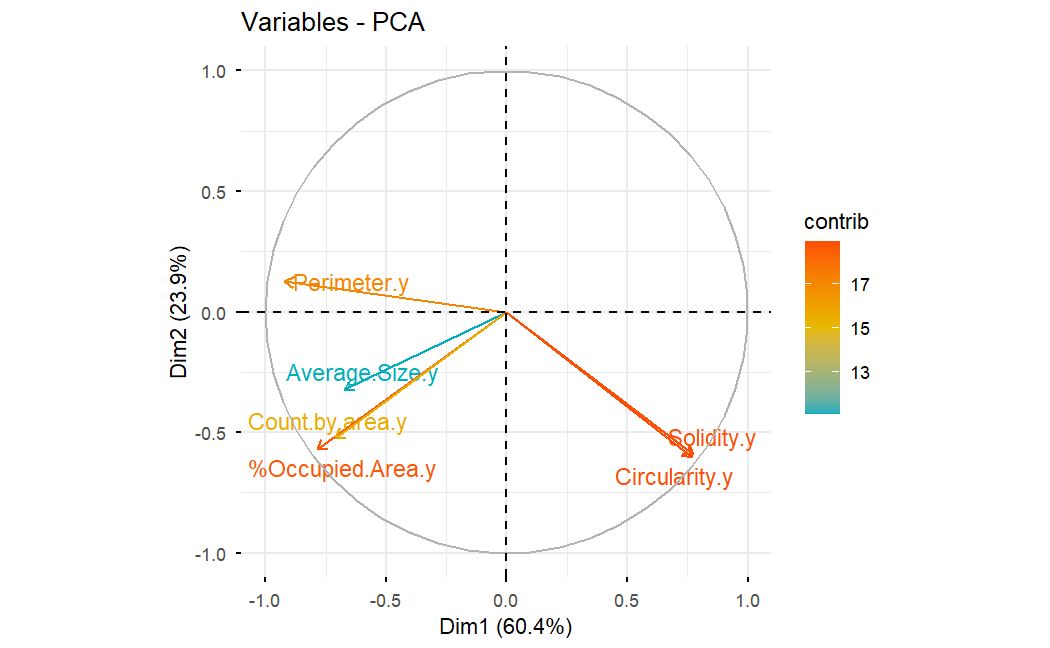**  **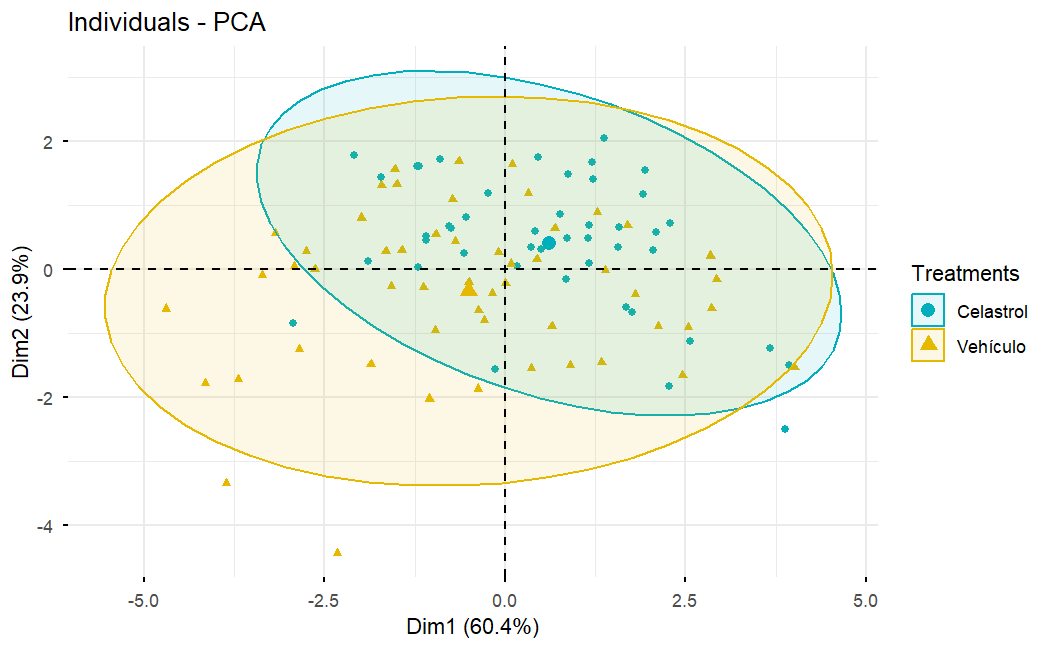**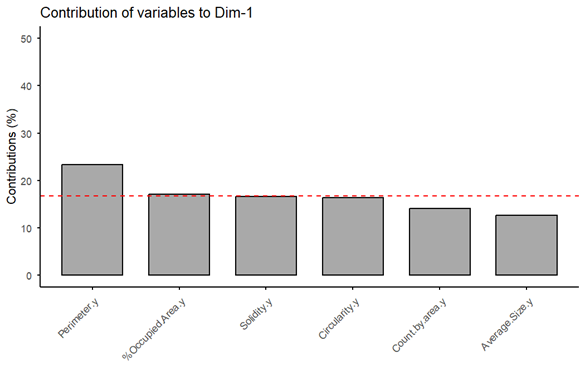 |
| --- |
| **Figure S14**. PCA and variables contribution on ARC astrocytes |

| **Variable** | **CTRL_cel** | **HFHS_cel** | **CTRL_veh** | **HFHS_veh** | **P_diet** | **P_treatment** |
| --- | --- | --- | --- | --- | --- | --- |
| Average.Size.y | 499.12 | 541.56 | 543.63 | 633.33 | 0.011 | **0.033** |
| perc_Occupied_Area_y | 0.98 | 1.54 | 1.54 | **2.75** | 0.009 | **0.032** |
| Perimeter.y | 183.45 | 193.45 | 185.42 | **214.65** | 0.036 | 0.317 |
| Circularity.y | 0.24 | 0.23 | 0.25 | 0.22 | 0.193 | 0.879 |
| Solidity.y | 0.54 | 0.53 | 0.55 | 0.52 | 0.463 | 0.727 |
| Count.by.area.y | 19.29 | 27.29 | 27.65 | 42.89 | 0.043 | 0.080 |
| **Table S15**. Mean values and tests of astrocytic ARC descriptors | | | | | | |

| **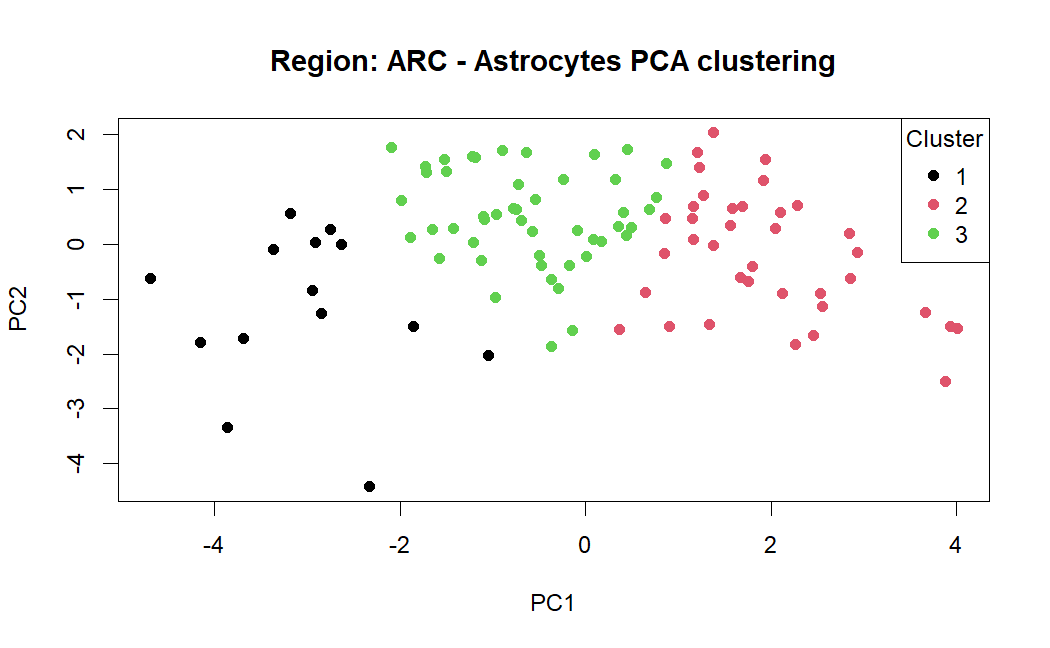** | clust Celastrol Vehicle  **1 0.07142857 0.92857143**  2 0.59459459 0.40540541  3 0.45833333 0.54166667    clust CTRL HFHS  **1 0.1428571 0.8571429**  2 0.7027027 0.2972973  3 0.4791667 0.5208333 |
| --- | --- |
| **Figure S15.** PCA k-means clustering on ARC astrocytic descriptors (left) and proportion tables of each cluster in terms of experimental conditions (right) | |

**HFHS vehicle group** characterized by very negative PCA1 and PCA2, which, in terms of contributing variables, translates into mainly high perimeter and %occupied area, but relatively lower solidity and circularity.

**PVN ASTROCYTES**

|  | **eigenvalue**  <dbl> | **variance.percent**  <dbl> | **cumulative.variance.percent**  <dbl> |
| --- | --- | --- | --- |
| Dim.1 | 3.647219865 | 60.7869978 | 60.78700 |
| Dim.2 | 1.700673282 | 28.3445547 | 89.13155 |
| **Table S16**. PCA eigenvaluesand variance explained on PVN astrocytes | | | |

| 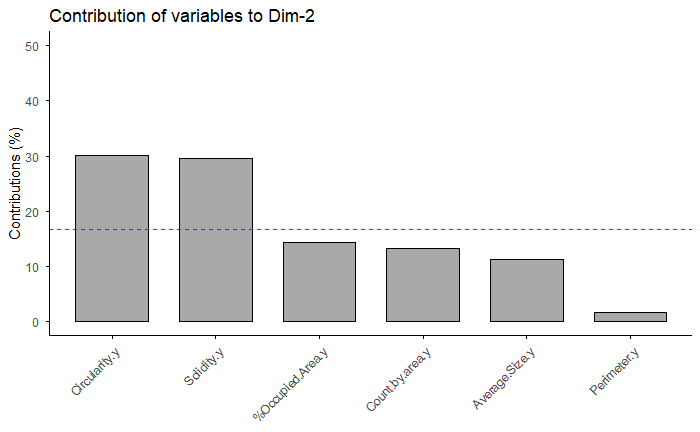**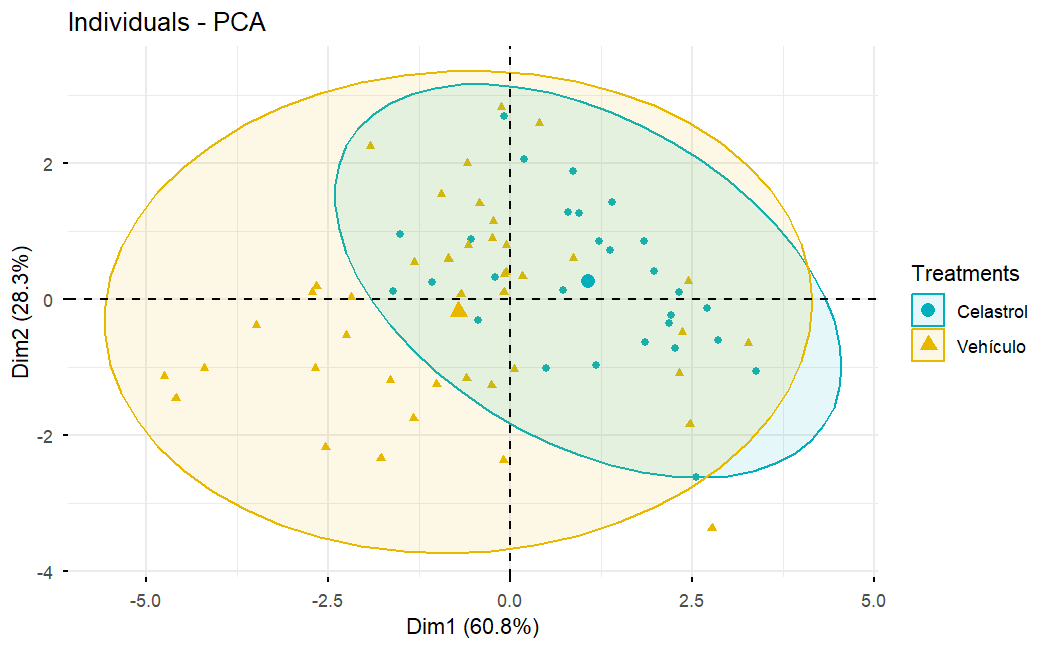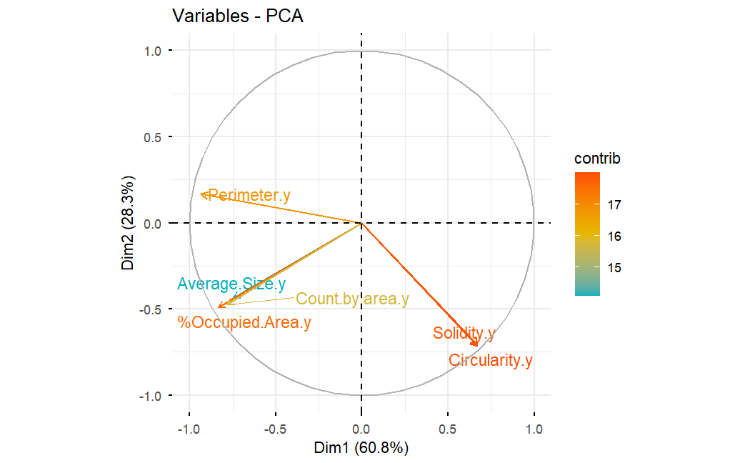**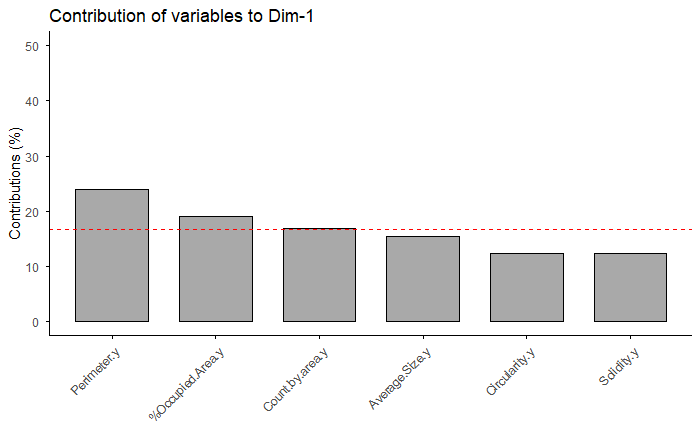 |
| --- |
| **Figure S16.** Variable contribution on PCA for PVN astrocytes |

| Variable | means_CTRL_cel | means_HFHS_cel | means_CTRL_Veh | means_HFHS_Veh | p_diet | p_treatment |
| --- | --- | --- | --- | --- | --- | --- |
| Average.Size.y | 417.75 | 392.64 | 464.47 | **509.25** | 0.402 | **0.000** |
| perc_Occupied_Area_y | 0.57 | 0.61 | 0.81 | **1.67** | 0.028 | **0.004** |
| Perimeter.y | 165.93 | 158.00 | 179.33 | 192.54 | 0.468 | 0.003 |
| Circularity.y | 0.24 | 0.24 | 0.22 | 0.21 | 0.634 | 0.079 |
| Solidity.y | 0.52 | 0.52 | 0.51 | 0.50 | 0.528 | 0.299 |
| Count.by.area.y | 13.39 | 15.15 | 16.28 | 31.71 | 0.023 | 0.013 |
| **Table S17**. Mean values and tests on astrocytic descriptors in the PVN | | | | | | |

| **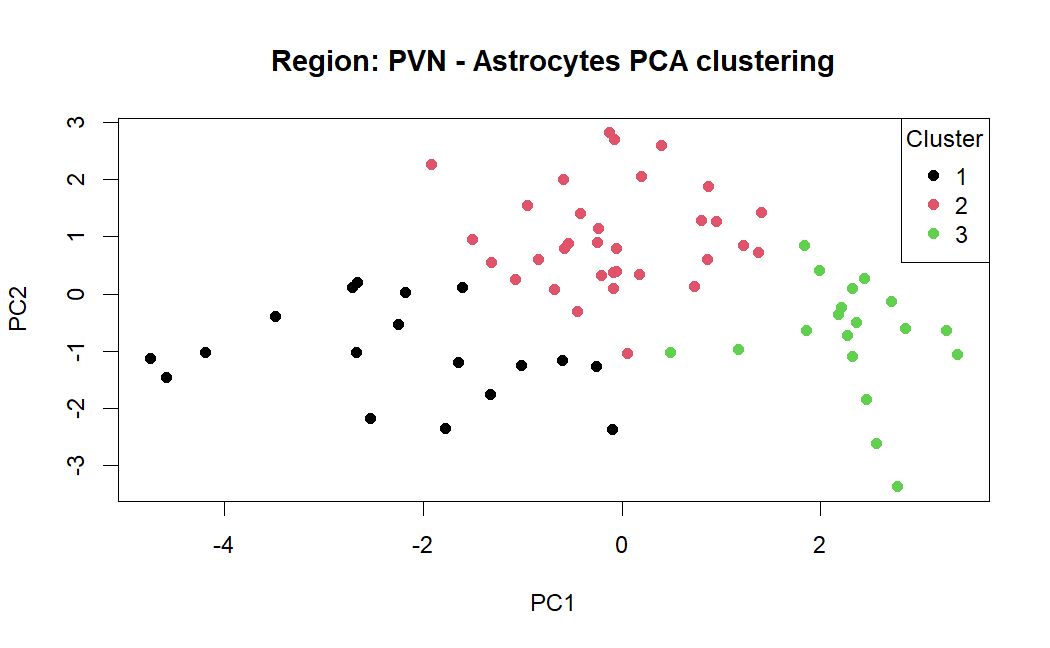** | clust Celastrol Vehicle  **1 0.05555556 0.94444444**  2 0.42424242 0.57575758  3 0.68421053 0.31578947    clust CTRL HFHS  **1 0.1111111 0.8888889**  2 0.5454545 0.4545455  3 0.6315789 0.3684211 |
| --- | --- |
| **Figure S17.** PCA k-means clustering on PVN astrocytic descriptors (left) and proportion tables of each cluster in terms of experimental conditions (right) | |

**HFHS vehicle group** characterized by very negative PCA with negative PCA2, which translates into mainly high average size and %occupied area, but relatively small solidity and circularity.

**VMN ASTROCYTES**

|  | **eigenvalue** | **variance.percent** | **cumulative.variance.percent** |
| --- | --- | --- | --- |
| Dim.1 | 3.17600944 | 52.9334907 | 52.93349 |
| Dim.2 | 1.64112405 | 27.3520675 | 80.28556 |
| **Table S18**. PCA eigenvalues and variance explained on VMN astrocytes | | | |

| **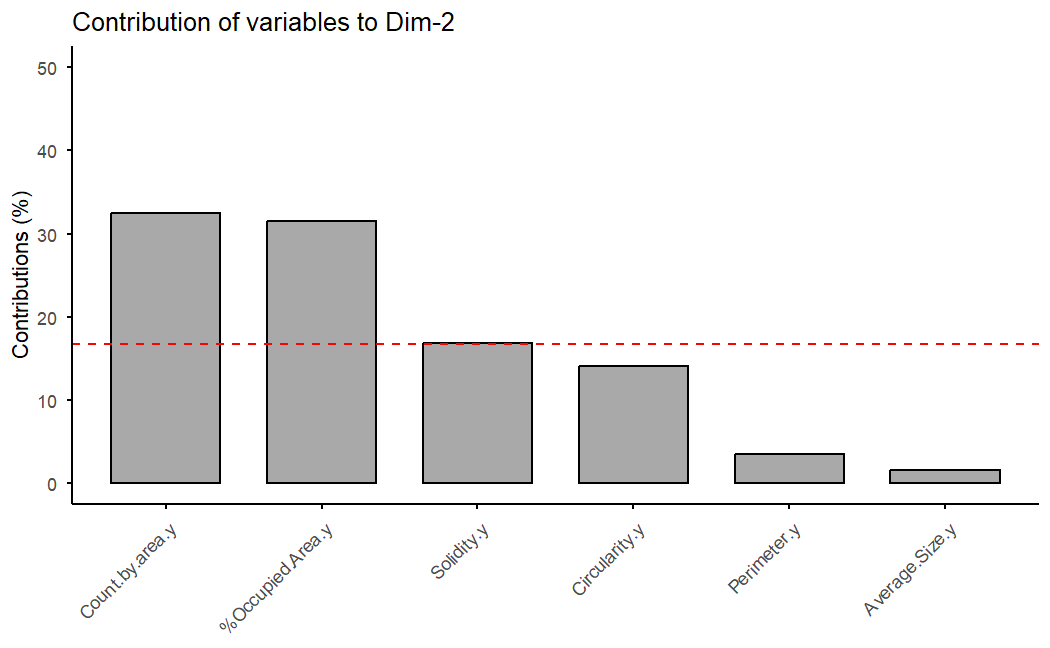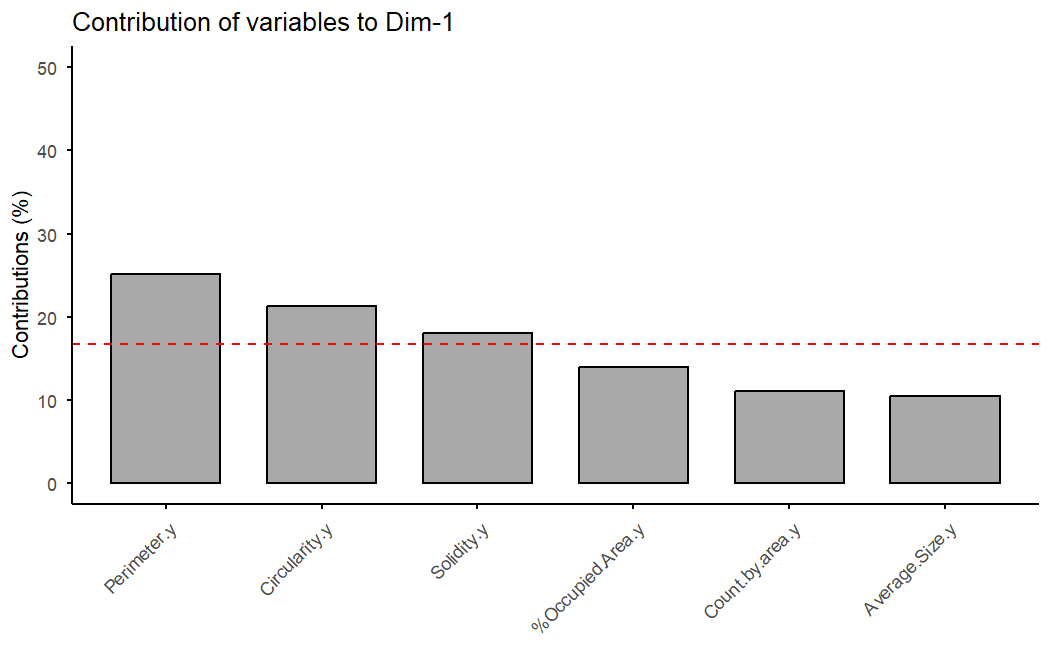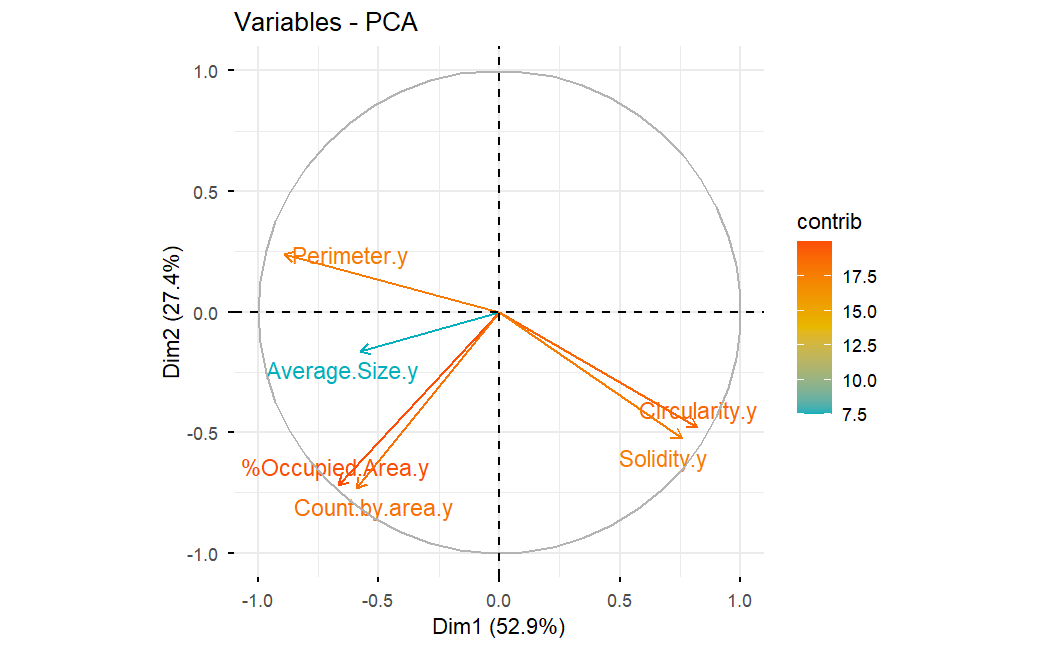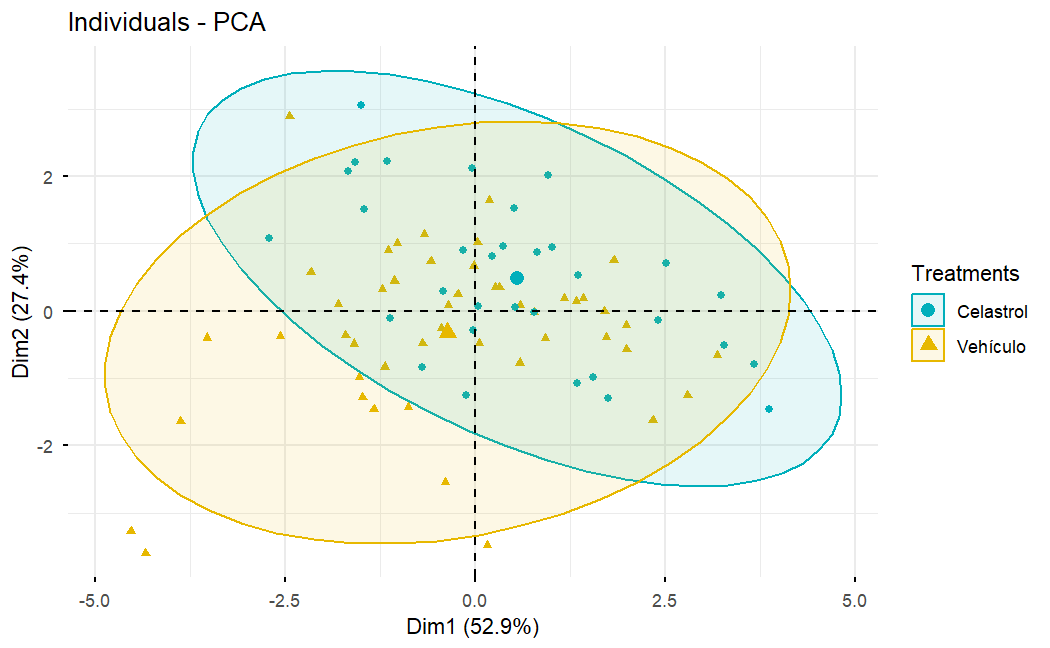** |
| --- |
| **Figure S18.** Variable contribution to PCA on VMN astrocytes**.** |

| **Variable** | **CTRL_cel** | **HFHS_cel** | **CTRL_veh** | **HFHS_veh** | **P_diet** | **P_treatment** |
| --- | --- | --- | --- | --- | --- | --- |
| Average.Size.y | 489.43 | 525.57 | 464.13 | 542.28 | 0.018 | 0.729 |
| perc_Occupied_Area_y | 0.38 | 0.42 | 0.60 | **1.15** | 0.046 | **0.027** |
| Perimeter.y | 179.42 | 182.91 | 173.51 | 197.72 | 0.089 | 0.773 |
| Circularity.y | 0.25 | 0.26 | 0.25 | 0.22 | 0.303 | 0.386 |
| Solidity.y | 0.54 | 0.58 | 0.55 | 0.52 | 0.907 | 0.403 |
| Count.by.area.y | 7.50 | 8.80 | 12.86 | **20.38** | 0.061 | **0.016** |
| **Table S19.** Mean values and tests on astrocytic descriptors in the VMN | | | | | | |

| **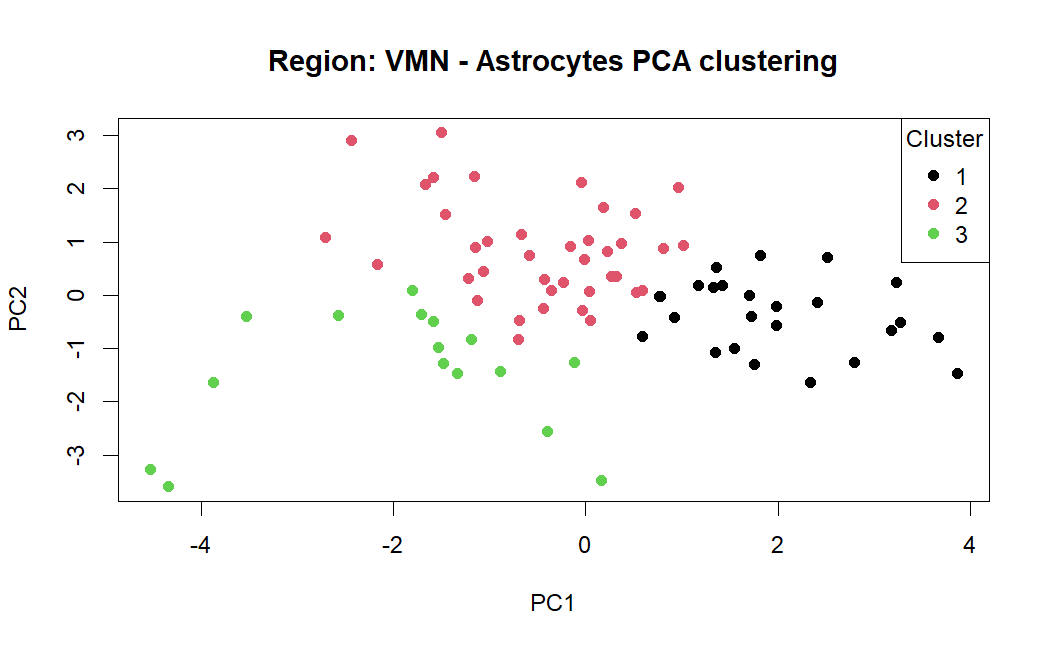** | clust Celastrol Vehicle  1 0.4400000 0.5600000  2 0.5128205 0.4871795  **3 0.0625000 0.9375000**    clust CTRL HFHS  1 0.6800000 0.3200000  2 0.6666667 0.3333333  **3 0.1875000 0.8125000** |
| --- | --- |
| **Figure S19.** PCA k-means clustering on VMN astrocytic descriptors (left) and proportion tables of each cluster in terms of experimental conditions (right) | |

**HFHS vehicle group** characterized by very negative PCA1 and PCA2, which, in terms of contributing variables, translates into mainly high perimeter with low circularity with high counts by area and %occupied area

**HIPPOCAMPUS ASTROCYTES**

|  | **eigenvalue** | **variance.percent** | **cumulative.variance.percent** |
| --- | --- | --- | --- |
| Dim.1 | 3.71009575 | 61.8349292 | 61.83493 |
| Dim.2 | 1.64575879 | 27.4293131 | 89.26424 |
| **Table S20**. PCA eigenvalues and variance explained on hippocampal astrocytes | | | |

| **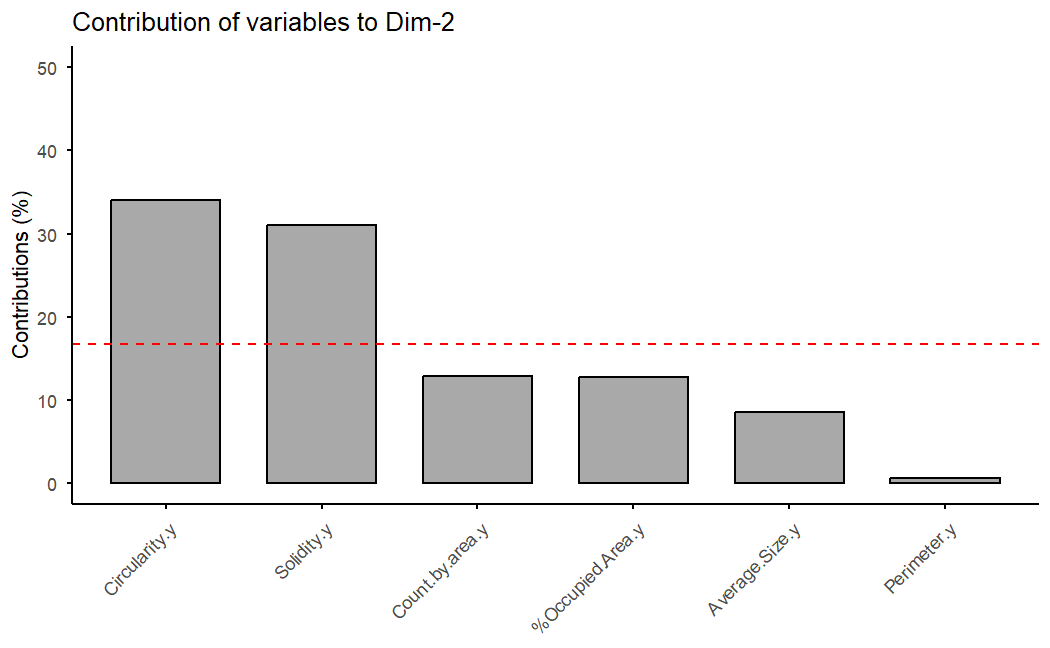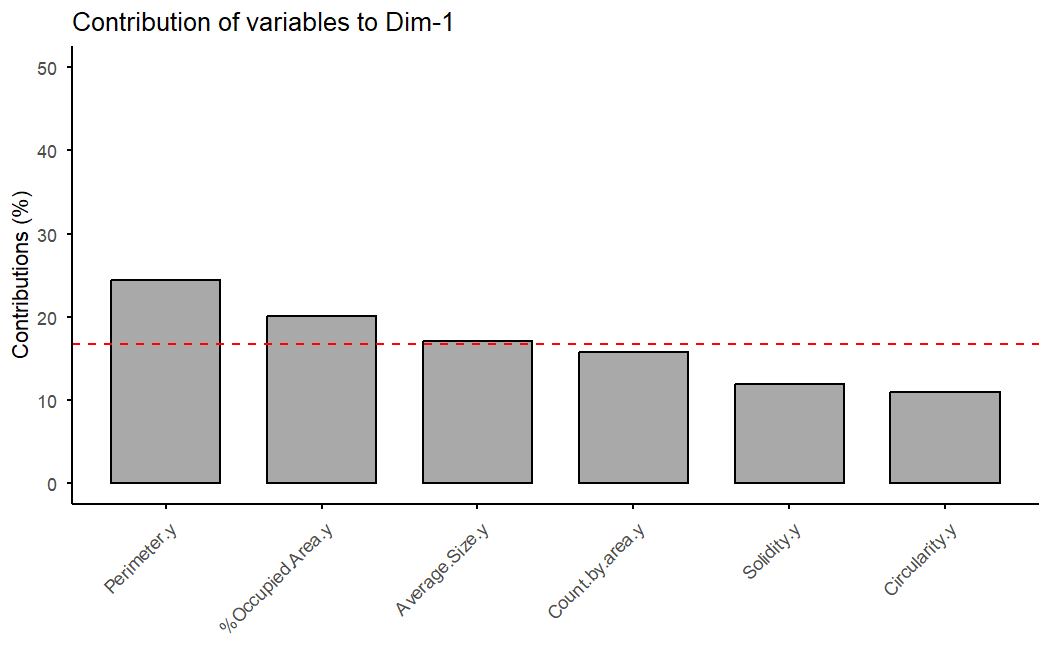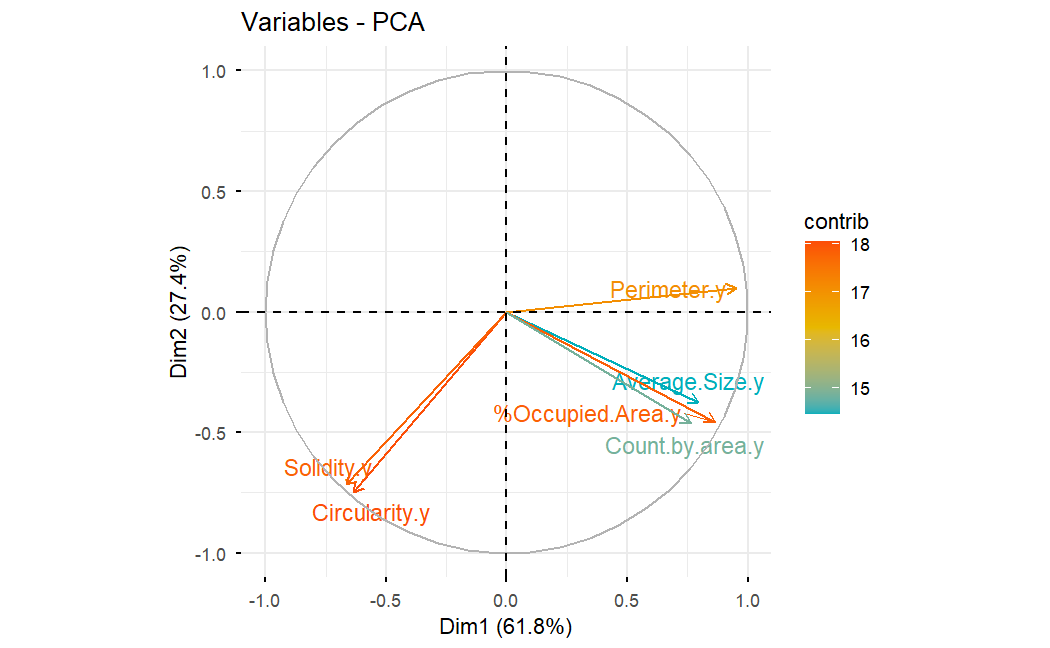** |
| --- |
| **Figure 20** Variable contribution to PCA on hippocampal astrocytes**.** |

| **Variable** | **CTRL_cel** | **HFHS_cel** | **CTRL_veh** | **HFHS_veh** | **P_diet** | **P_treatment** |
| --- | --- | --- | --- | --- | --- | --- |
| Average.Size.y | 470.90 | 493.57 | 524.93 | **597.94** | 0.147 | 0.011 |
| Circularity.y | 0.25 | 0.24 | 0.27 | 0.23 | 0.027 | 0.364 |
| Count.by.area.y | 22.47 | 26.51 | 23.78 | 35.98 | 0.019 | 0.119 |
| perc_Occupied_Area_y | 1.11 | 1.41 | 1.28 | **2.23** | 0.010 | 0.039 |
| Perimeter.y | 169.53 | 177.12 | 172.68 | 200.36 | 0.027 | 0.129 |
| Solidity.y | 0.55 | 0.54 | 0.57 | 0.54 | 0.058 | 0.331 |
| **Table S21.** Mean values and tests on astrocytic descriptors in the HIPP | | | | | | |

| **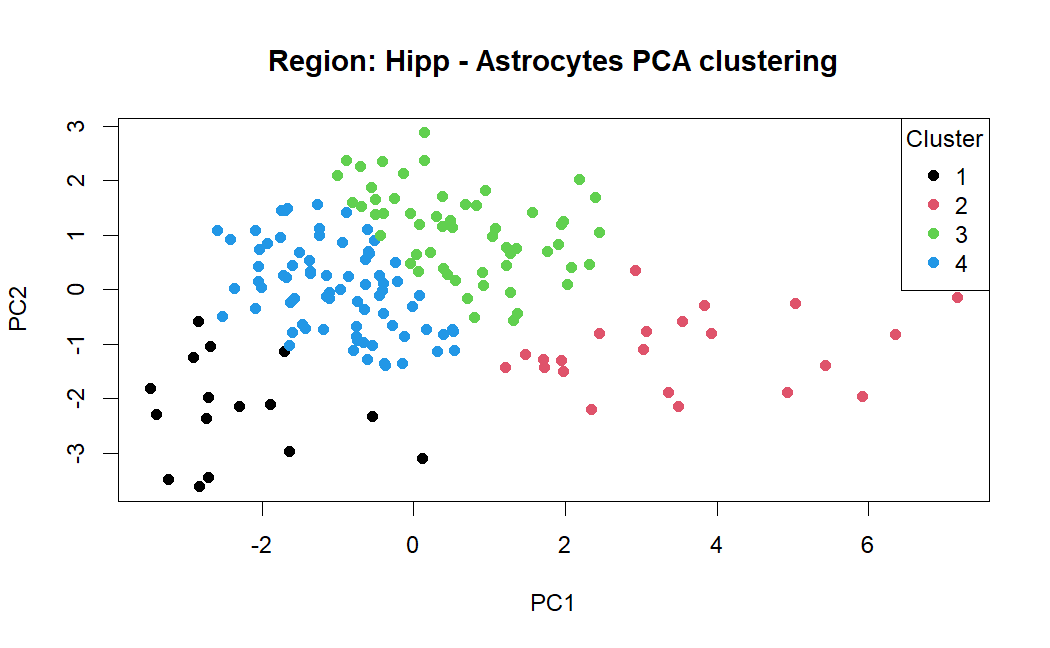** | clust Celastrol Vehicle  1 0.3750000 0.6250000  **2 0.2272727 0.7727273**  3 0.5714286 0.4285714  4 0.6000000 0.4000000    clust CTRL HFHS  1 0.6875000 0.3125000  **2 0.1363636 0.8636364**  3 0.4285714 0.5714286  4 0.5466667 0.4533333 |
| --- | --- |
| **Figure S21.** PCA k-means clustering on HIPP astrocytic descriptors (left) and proportion tables of each cluster in terms of experimental conditions (right) | |

HFHS vehicle group (86% HFHS 78% vehicle) with high PCA1, which translates into high perimeter, %occupied area and size.

**NAC ASTROCYTES**

|  | **eigenvalue** | **variance.percent** | **cumulative.variance.percent** |
| --- | --- | --- | --- |
| Dim.1 | 2.85659757 | 47.6099596 | 47.60996 |
| Dim.2 | 1.82908445 | 30.4847408 | 78.09470 |
| Dim.3 | 1.02974765 | 17.1624609 | 95.25716 |
| **Table S22**. PCA eigenvalues and variance explained on NAc astrocytes | | | |

| **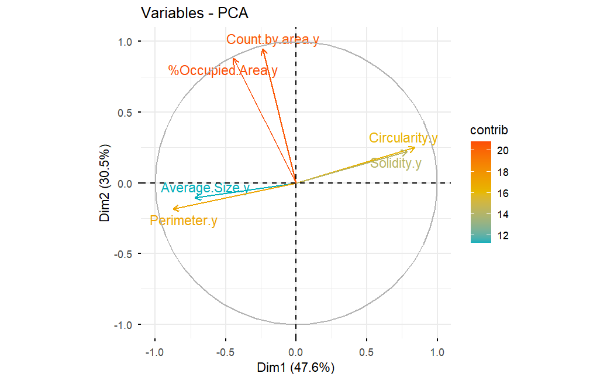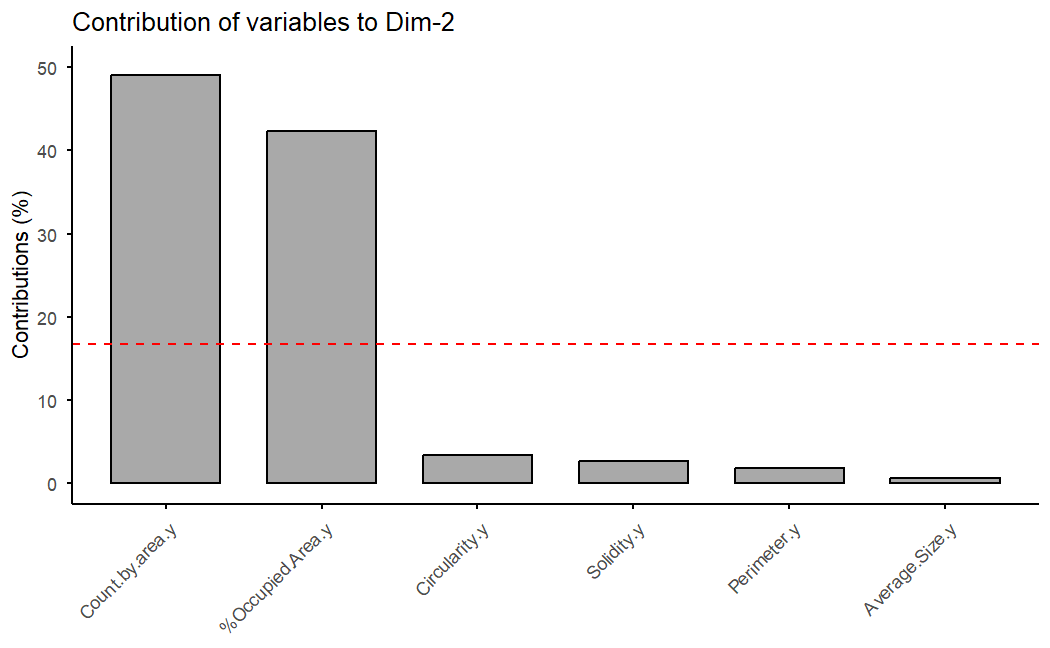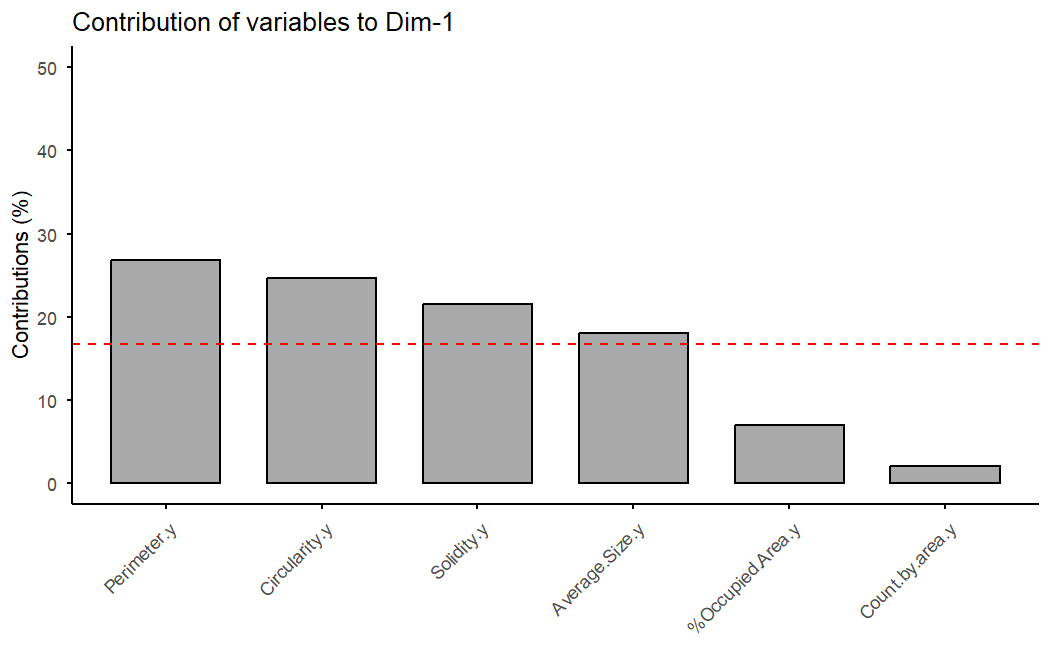** |
| --- |
| **Figure S22** Variable contribution to PCA on NAc astrocytes |

| **Variable** | **CTRL_cel** | **HFHS_cel** | **CTRL_veh** | **HFHS_veh** | **P_diet** | **P_treatment** |
| --- | --- | --- | --- | --- | --- | --- |
| **Average.Size.y** | 355.74 | 366.80 | 399.03 | 396.26 | 0.814 | 0.671 |
| **perc_Occupied_Area_y** | 0.12 | 0.16 | 0.08 | 0.22 | 0.000 | 0.321 |
| **Perimeter.y** | 164.32 | 159.42 | 178.24 | 169.12 | 0.629 | 0.968 |
| **Circularity.y** | 0.20 | 0.24 | 0.19 | 0.22 | 0.243 | 0.486 |
| **Solidity.y** | 0.55 | 0.57 | 0.54 | 0.56 | 0.551 | 0.567 |
| **Count.by.area.y** | 3.38 | 4.44 | 1.97 | 5.47 | 0.000 | 0.786 |
| **Table S23.** Mean values and tests on astrocytic descriptors in the NAc | | | | | | |

NO treatment effects reported in the NAc astrocytes

| **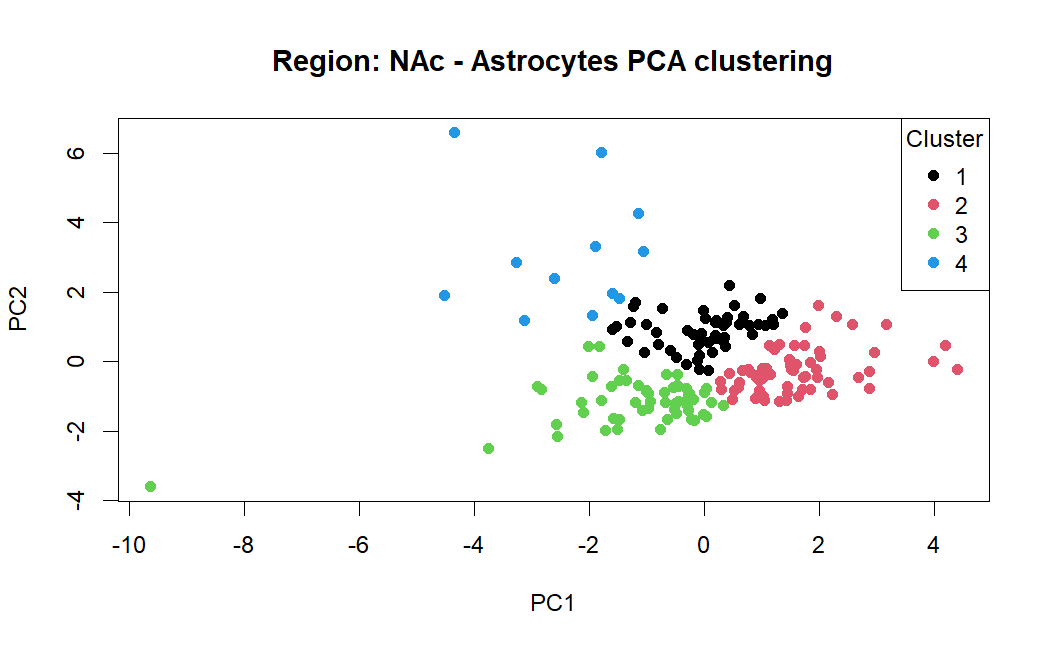** | clust Celastrol Vehicle  1 0.5400000 0.4600000  2 0.5593220 0.4406780  3 0.4736842 0.5263158  4 0.2500000 0.7500000    clust CTRL HFHS  1 0.3000000 0.7000000  2 0.5932203 0.4067797  3 0.6315789 0.3684211  **4 0.1666667 0.8333333** |
| --- | --- |
| **Figure S23.** PCA k-means clustering on NAc astrocytic descriptors (left) and proportion tables of each cluster in terms of experimental conditions (right) | |

HFHS vehicle group (80% HFHS 75% vehicle), high PCA2. High counts by area and %occupied area,

**ILA ASTROCYTES**

|  | **eigenvalue** | **variance.percent** | **cumulative.variance.percent** |
| --- | --- | --- | --- |
| Dim.1 | 2.87805359 | 47.9675599 | 47.96756 |
| Dim.2 | 1.89296259 | 31.5493765 | 79.51694 |
| Dim.3 | 1.06515674 | 17.7526124 | 97.26955 |
| **Table S24**. PCA eigenvalues and variance explained on ILA astrocytes | | | |

| **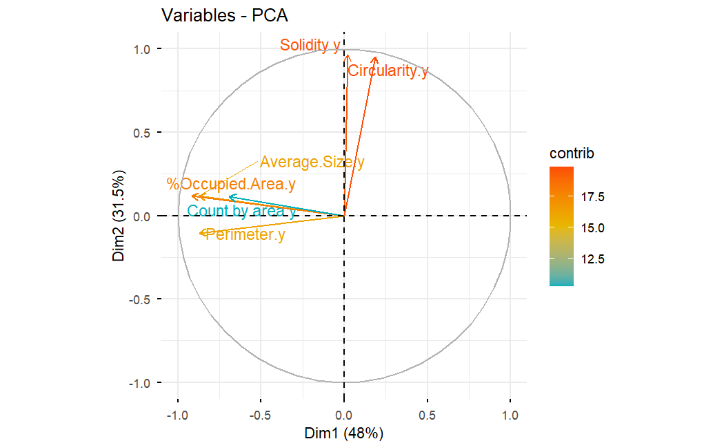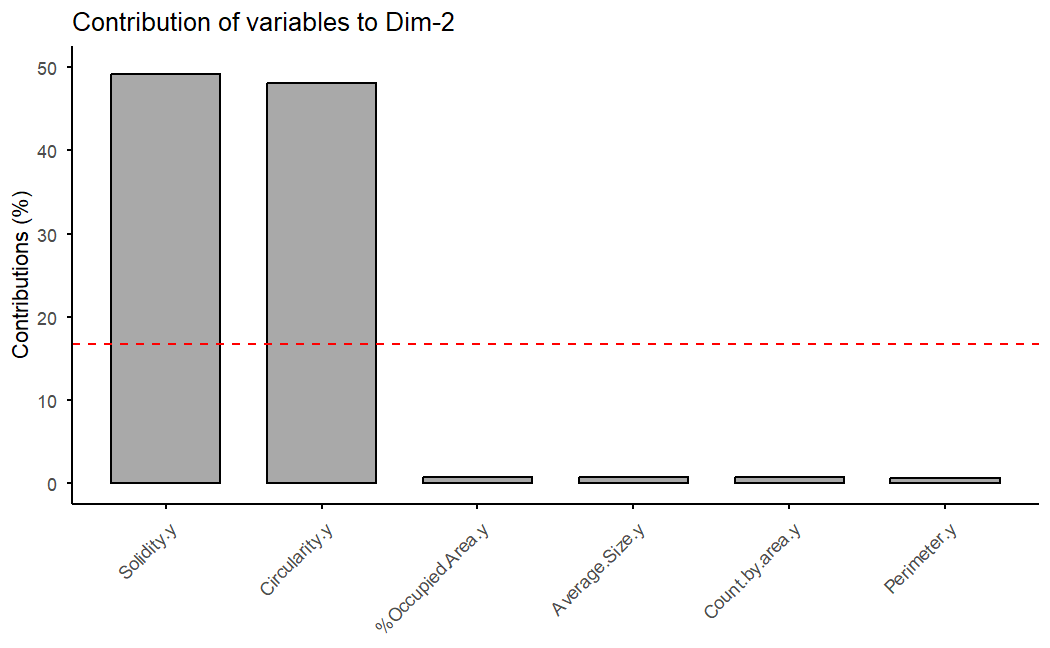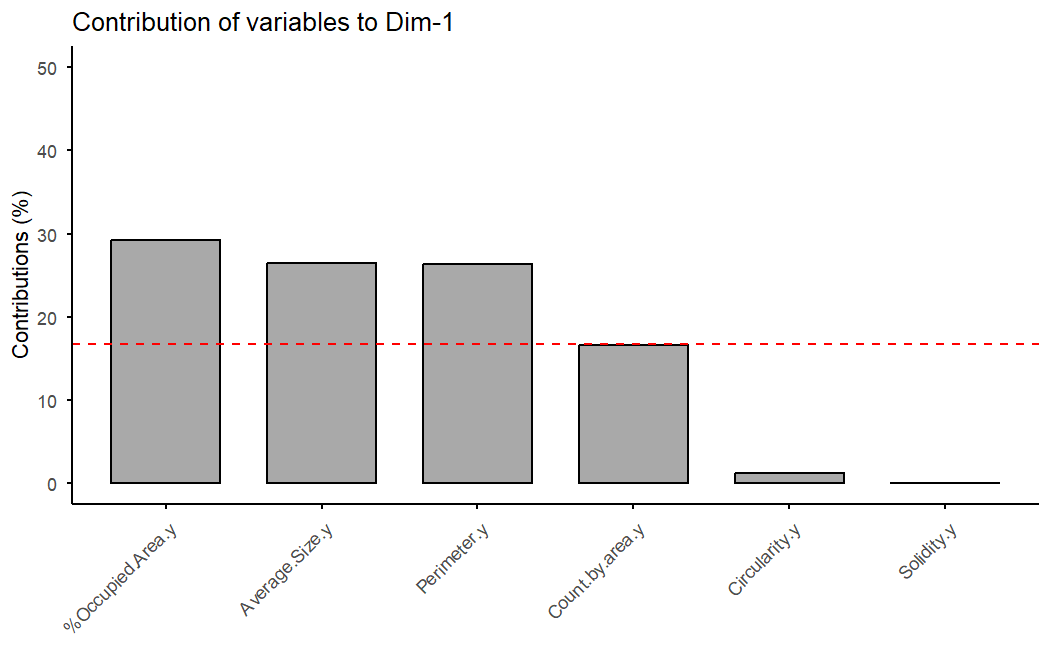** |
| --- |
| **Figure S24** Variable contribution to PCA on ILA astrocytes |

| **Variable** | **CTRL_cel** | **HFHS_cel** | **CTRL_veh** | **HFHS_veh** | **P_diet** | **P_treatment** |
| --- | --- | --- | --- | --- | --- | --- |
| Average.Size.y | 596.80 | 697.09 | 527.20 | 710.90 | 0.086 | 0.940 |
| perc_Occupied_Area_y | 1.72 | 1.65 | 0.92 | 0.99 | 0.808 | 0.140 |
| Perimeter.y | 232.37 | 248.03 | 226.54 | 266.12 | 0.182 | 0.629 |
| Circularity.y | 0.18 | 0.20 | 0.18 | 0.18 | 0.325 | 0.165 |
| Solidity.y | 0.50 | 0.53 | 0.49 | 0.52 | 0.048 | 0.294 |
| Count.by.area.y | 27.42 | 22.39 | 15.05 | 12.23 | 0.471 | 0.056 |
| **Table S25.** Mean values and tests on astrocytic descriptors in the ILA | | | | | | |

No treatment effects on astrocytes in the ILA region

| 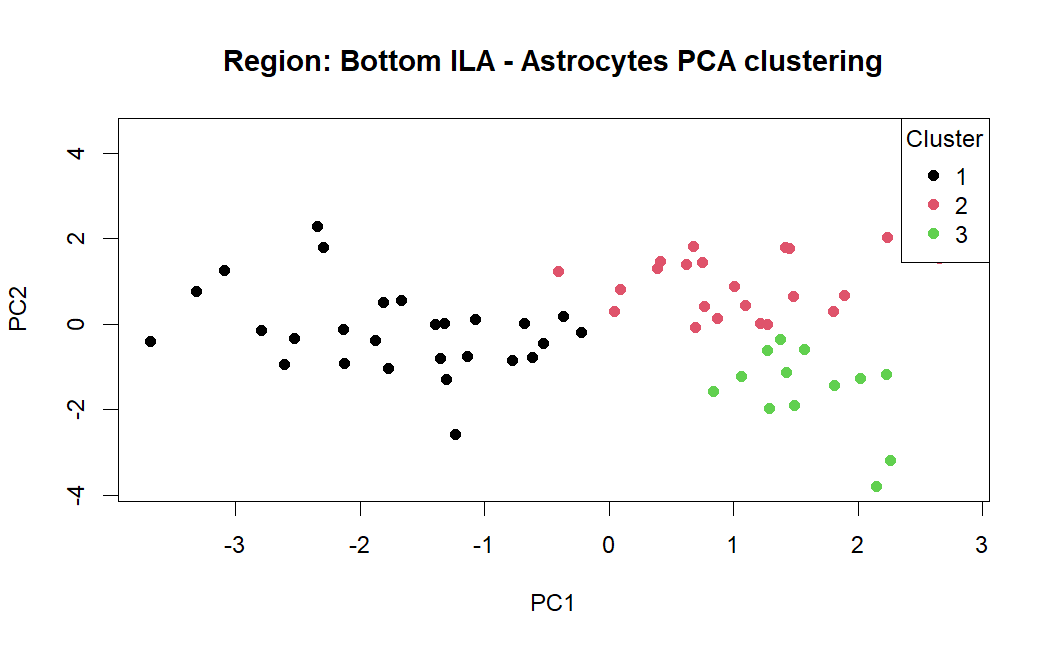 | clust Celastrol Vehicle  1 0.5185185 0.4814815  2 0.4782609 0.5217391  3 0.1538462 0.8461538    clust CTRL HFHS  1 0.4444444 0.5555556  2 0.4782609 0.5217391  3 0.5384615 0.4615385 |
| --- | --- |
| **Figure S25.** PCA k-means clustering on ILA astrocytic descriptors (left) and proportion tables of each cluster in terms of experimental conditions (right) | |

|  | **Microglia** | **Astrocytes** |
| --- | --- | --- |
| ARC | High perimeter and %occupied area | High perimeter and %occupied area |
| PVN | (87%vehicle 67% HFHS) with small circularity and solidity but high perimeter and %occupied area. | high average size and %occupied area, but relatively small solidity and circularity |
| VMN | high perimeter, counts and %occupied area but low solidity | high perimeter with low circularity with high counts by area and %occupied area |
| Hipp | high perimeter with low solidity and low circularity with high average size | (80% HFHS 77% vehicle) high perimeter, %occupied area and size |
| NAc | No cluster | group (80% HFHS 75% vehicle), high PCA2. High counts by area and %occupied area. |

No clear HFHS vehicle group (neither with 3 nor with 4 clusters)

**SUMMARY OF HFHS clusters**

**Table S26. HFHS vehicle cluster group** characteristics, as compared to the rest

|  | **Microglia** | **Astrocytes** |
| --- | --- | --- |
| ILA | into high perimeter but low solidity and circularity and high counts by area | No cluster |
| NAc | high perimeter and high average size with low solidity and circularity | group (80% HFHS 75% vehicle), high PCA2. High counts by area and %occupied area. |

**Table S27. HFHS cluster**

**REFERENCES**

R Core Team. R: A Language and Environment for Statistical Computing. R Foundation for Statistical Computing. Found. Stat. Comput. Vienna Austria [Internet]. 2025; Available from: https://www.r-project.org/

Ringnér M. What is principal component analysis? Nat. Biotechnol. [Internet]. 2008 Mar [cited 2025 Jan 30];26(3):303–4. Available from: https://www.nature.com/articles/nbt0308-303
